# Impact of financial inclusion in low‐ and middle‐income countries: A systematic review of reviews

**DOI:** 10.4073/csr.2019.2

**Published:** 2019-07-23

**Authors:** Maren Duvendack, Philip Mader

**Affiliations:** ^1^ School of International Development University of East Anglia Norwich NR4 7TJ UK; ^2^ Institute of Development Studies University of Sussex Brighton East Sussex BN19RE UK

## 1 PLAIN LANGUAGE SUMMARY

### 1.1 Financial inclusion interventions have very small and inconsistent impacts

A wide range of financial inclusion programmes seek to increase poor people's access to financial services to enhance the welfare of poor and low‐income households in low‐ and middle‐income countries. The impacts of financial inclusion interventions are small and variable. Although some services have some positive effects for some people, overall financial inclusion may be no better than comparable alternatives, such as graduation or livelihoods interventions.

### 1.2 What is this review about?

Financial inclusion programmes seek to increase access to financial services such as credit, savings, insurance and money transfers and so allow poor and low‐income households in low‐ and middle‐income countries to enhance their welfare, grasp opportunities, mitigate shocks, and ultimately escape poverty. This systematic review of reviews assesses the evidence on economic, social, behavioural and gender‐related outcomes from financial inclusion.

**What is the aim of this review?**

This systematic review of reviews systematically collects and appraises all of the existing meta‐studies—that is systematic reviews and meta‐analyses—of the impact of financial inclusion. The authors first analyse the strength of the methods used in those meta‐studies, then synthesize the findings from those that are of a sufficient quality, and finally, report the implications for policy, programming, practice and further research arising from the evidence. Eleven studies are included in the analysis.

### 1.3 What are the main findings of this review?

#### 1.3.1 What studies are included?

This review includes studies that synthesise the findings of other studies (meta‐studies) regarding the impacts of a range of financial inclusion interventions on economic, social, gender and behavioural outcomes. A total of 32 such meta‐studies were identified, of which 11 were of sufficient methodological quality to be included in the final analysis. The review examined meta‐studies from 2010 onwards that spanned the globe in terms of geographical coverage.

Impacts are more likely to be positive than negative, but the effects vary, are often mixed, and appear not to be transformative in scope or scale, as they largely occur in the early stages of the causal chain of effects. Overall, the effects of financial services on core economic poverty indicators such as incomes, assets or spending, and on health status and other social outcomes, are small and inconsistent. Moreover, there is no evidence for meaningful behaviour‐change outcomes leading to further positive effects.

The effects of financial services on women's empowerment appear to be generally positive, but they depend upon programme features which are often only peripheral or unrelated to the financial service itself (such as education about rights), cultural and geographical context, and what aspects of empowerment are considered.

Accessing savings opportunities appears to have small but much more consistently positive effects for poor people, and bears fewer downside risks for clients than credit. A large number of the meta‐studies included in the final analysis voiced concerns about the low quality of the primary evidence base that formed the basis of their syntheses. This raises concerns about the reliability of the overall findings of meta‐studies.

### 1.4 What do the findings of this review mean?

This systematic review of reviews draws on the largest‐ever evidence base on financial inclusion impacts. The weak effects found warn against unrealistic hype for financial inclusion, as previously happened for microcredit. There are substantial evidence gaps, notably studies of sufficient duration to measure higher‐level impacts which take time to materialise, and for specific outcomes such as debt levels or indebtedness patterns and the link to macroeconomic development.

This study is the first review of reviews published by the Campbell Collaboration. Some important limitations were encountered working at this level of systematisation. It is recommended that authors of primary studies and meta‐studies engage more critically with study quality and ensure better, more detailed reporting of their concepts, data and methods. More methods guidance and clearer reporting standards for the social science and international development context would be helpful.

### 1.5 How up‐to‐date is this review?

The review authors searched for studies in November 2017, updating elements of the searches in January 2018. This Campbell Systematic Review was published in January 2019.

## 2 EXECUTIVE SUMMARY/ABSTRACT

### 2.1 Background

Financial inclusion is presently one of the most widely recognised areas of activity in international development. Financial inclusion initiatives have built upon donors’ experience with microfinance, but have displaced and superseded microfinance interventions in recent years with a more encompassing agenda of financial services for poverty alleviation and development. With financial inclusion, policymakers and donors hope that access to financial services (including credit, savings, insurance and money transfers) provided by a variety of financial service providers, of which microfinance institutions (MFIs) are a subset, will allow poor and low‐income households in low‐ and middle‐income countries to enhance their welfare, grasp opportunities, mitigate shocks, and ultimately escape poverty. Another hope is that increased access to financial services will advance macroeconomic development, which is also expected to benefit poor/low‐income households. More recently, some donors have suggested behavioural changes (such as household spending decisions) to be desired outcomes of access to financial services, as well. Unlike most previous systematic reviews, which focused on microfinance interventions (or subsets thereof), we explicitly adopt a broader scope to review any available systematic review and or meta‐analysis evidence on financial inclusion as a whole field.

Systematic reviews and meta‐analyses (in short: meta‐studies) have sought to clarify the impacts from financial inclusion on poor people in low‐ and middle‐income countries, based on an array of different underlying studies which include quantitative and qualitative work based on long‐term and short‐term data. The bulk of these meta‐studies have focused on microfinance, and many specifically on microcredit. The very different quality and approaches of these meta‐studies, and of the studies underlying them, however, pose a major challenge for policymakers, programme managers and practitioners in assessing the benefits and drawbacks of finance‐based approaches to poverty alleviation. Increasingly there is confusion about the impacts, and a risk of “cherry picking” among different findings. Further, many meta‐studies are not taking into account what is missing from their primary studies, which would affect an understanding of the evidence, for example when not analysing or reporting gendered impacts. More recently, primary studies have also sought to understand the impacts of financial inclusion initiatives more broadly, but the systematic review evidence has not yet progressed as far as for microfinance.

### 2.2 Objectives

The objective of this systematic review of reviews is to systematically collect and appraise the existing meta‐studies of financial inclusion impacts, analyse the strength of the methods used, synthesise the findings from those meta‐studies, and report implications for policy, programming, practice and further research.

Systematic reviews of reviews are undertaken in other sectors for which evidence is widely available, but they are nonexistent in international development. This systematic review of reviews thus provides the opportunity to develop and pilot an evidence synthesis approach in a sector where there is a large body of evidence of variable quality, but a systematic appraisal and synthesis of the body of systematic reviews and meta‐analyses is lacking.

This study critically engages with approaches to systematic reviews of reviews with a view to further developing systematic review of review methods, and it aims to answer the following questions to gain better clarity about financial inclusion impacts:

- Impacts
  ○ What is known from existing meta‐studies about the (social, economic and behavioural) poverty impacts of different types of inclusive financial services (e.g., credit, savings, insurance and money transfers), regardless of provider, on poor and low‐income people in low‐ and middle‐income countries? This includes poverty impacts through macroeconomic development, to the extent that it results from financial inclusion.
  ○ What is known from existing meta‐studies about the gendered impacts of different types of financial inclusion activity (e.g., credit, savings, insurance and money transfers)—in other words, what does the evidence tell us about how gendered participation affects interventions’ effects, and about whether or not (and in what ways) financial services empower women in low‐ and middle‐income countries?
  ○ What is known from existing meta‐studies about the reasons for financial services uptake, or other participant views about the financial services on offer?
- Methodology
  ○ Including using a gender and equity lens, what methods and standards have meta‐studies used to draw conclusions from the studies they reviewed?
  ○ What difference does the choice of methods and standards make to the results?
  ○ How could the methods and standards be improved in order to draw more robust and reliable conclusions via meta‐studies?

### 2.3 Search methods

We adopt a multipronged search strategy that explored seven bibliographic databases to identify published literature, plus a wide range of institutional websites for published and unpublished literature, and back‐referencing from recent systematic reviews to ensure additional sources were identified. In addition, a snowballing approach was adopted and an advisory board plus leading authors working on financial inclusion topics were consulted to ensure that no key studies were missed. We also ran citation searches on included systematic reviews and meta‐analyses in Google Scholar, Scopus and Web of Science to identify more recent systematic reviews or meta‐analyses not retrieved in the database searches. No restrictions were placed on the language of papers but all searches were limited to 2010 onwards.

### 2.4 Selection criteria

We adopted the following selection criteria to establish study inclusion or exclusion:

#### 2.4.1 Types of reviews

We include studies that self‐identify as systematic reviews and or meta‐analyses of the impacts of financial inclusion (including, but not limited to, microfinance). These, in turn, focused on synthesising quantitative, qualitative and or mixed methods evidence.

#### 2.4.2 Types of participants

Our population is the population of participants in inclusive finance activities in low‐ and middle‐income countries.

#### 2.4.3 Types of interventions

We include meta‐studies that address at least one or more types of intervention for financial inclusion. The key is that the intervention must be fundamentally a financial service directed at poor and low‐income people. In most cases, we find the interventions are one or more subcategories of microfinance: credit, savings, insurance, leasing, and/or money transfers. However, our search strategy explicitly targets the broader range of inclusive finance activities, including mobile monies, mobile payments systems, index insurance, or savings promotion.

#### 2.4.4 Types of outcome measures

Meta‐studies capturing a wide range of poverty indicators (including income, assets, expenditure, personal networks, gender/empowerment, well‐being, health, etc.) are included.

All meta‐studies were screened by two research assistants independently, with the two review authors independently reviewing each meta‐study marked for inclusion. Full texts were obtained and screened when a decision could not be made; an arbitration procedure was in place in case of disagreements.

### 2.5 Data collection and analysis

A total of 32 meta‐studies were identified after completing the screening process. However, only 11 of these were assessed to be of sufficient methodological quality to be included in the final analysis. We note that a large number of these meta‐studies voiced concerns about the low quality of the primary evidence base that formed the basis of their syntheses, which in turn raises concerns about the reliability of the overall findings presented at the review level. Combining a wide range of low quality studies into systematic reviews to aggregate their findings is risky.

A coding tool was developed to extract data from the included meta‐studies on the following areas of interest:

1. Context
2. Type of intervention
3. Type of review, design, and methods used
4. Outcome measures
5. Quality assessment
6. Study results and findings.

Data were extracted at the meta‐study level. However, for meta‐studies classified as high‐ and medium‐confidence, when necessary, we also extracted information at the primary study level.

The synthesis of results was guided by a theory‐based mixed methods synthesis approach with a focus on a narrative synthesis that incorporates quantitative elements as appropriate.

### 2.6 Results

Five out of the 11 (medium‐ and high‐confidence) meta‐studies that we reviewed drew largely positive conclusions about the relationship between financial services access and changes for poor people, and the other six drew largely mixed, neutral, or unclear conclusions. The detailed review of the evidence base uncovered a nuanced picture, reflecting large variations across the effects of different interventions and for different people in different contexts. Findings across the reviews were heterogeneous and often inconsistent, both within and across reviews, and many reviews did not find evidence of expected or presumed impacts.

The present high‐level evidence does not suggest that financial inclusion initiatives have transformative effects. On average, financial services may not even have a meaningful net positive effect on poor or low‐income users, although some services have some positive effects for some people. Overall, we find:

- The impacts are more likely to be positive than negative, but the effects vary, are often mixed, and appear not to be transformative in scope or scale, as they largely occur in the early stages of the causal chain.
- The effects of financial services on core economic poverty indicators such as incomes, assets or spending are small and inconsistent.
- The effects of financial services on women's empowerment appear to be generally positive, but they depend upon programme features (which are often only peripheral or unrelated to the financial service itself, for instance exposure to women's rights), context, and what aspects of empowerment are considered, and their assessment is confounded by a difficulty of consistently conceptualising and measuring empowerment.
- The effects of credit and other financial services on health status and other social outcomes appear to be small or nonexistent.
- There is no evidence for meaningful behaviour‐change outcomes leading to further positive effects.
- Accessing savings opportunities appears to have small but much more consistently positive effects for poor people, and bears fewer downside risks for clients than credit.

Many of the primary studies that were included in the meta‐studies we analysed in depth had medium or even high risk of bias, due to their study design, poor reporting of methodology, and other causes. As some of the meta‐studies highlighted, it is mainly the higher risk of bias studies that drive most of the positive impact estimates. Our findings thus broadly confirm the “stainless steel” law of evidence that, the more rigorous and lower risk of bias studies become, the less likely they are to find effects. This applies to both our reviews and to the underlying primary evidence that they have reviewed. Given that the reviews we classified as being of lower methodological quality were more likely to report positive effects, we must treat their positive findings with caution.

In summary, almost all effect sizes we find are quite small and hardly indicative of transformative changes from financial inclusion, and are found dominantly on lower‐order or intermediate outcomes. Many effects are strongly heterogeneous, both across studies and over time, places, populations, gender, ethnicity and between interventions; this suggests them to be unreliable and/or context‐dependent. Positive findings tend not to repeat from one context, intervention type or study to another, and at least as many findings are mixed or inconclusive as are positive. As a result, the positive results found for financial inclusion are fragile, and need to be treated with caution. An exception appears to be with regard to savings, where both immediate outcomes and wider poverty measures are affected in a positive, but relatively small, way; however, we base this mainly on the findings of one high confidence meta‐analysis (Steinert et al., 2018). There is no savings “revolution” going on, but savings at least appear to do some good and no harm.

### 2.7 Authors’ conclusions

We have taken the evolution of the financial inclusion impact literature toward a natural conclusion, with a higher level of evidence systematisation, to provide an overview of what has become an increasingly perplexing array of meta‐studies that each offer partial overviews. By reviewing these reviews, we have drawn on what is likely the largest‐ever evidence base on financial inclusion impacts, and have uncovered strengths, gaps and weaknesses of the existing high‐level evidence. We hope that we have reduced the amount of confusion and uncertainty arising from the many different meta‐studies on financial inclusion published in recent years, not least thanks to our systematic assessment of the variations in quality within that field.

The (perhaps boring) truth that seems to emerge about financial inclusion is that it is not changing the world. On average, financial services may not even have a meaningful net positive effect on poor or low‐income users, although some services have some positive effects for some people. Considering that for most people financial services (whether they can access them, and how they use them) will be only one among many possible determinants of their life chances and their socioeconomic well‐being, this finding ought not to be unexpected, and we anticipate that it will be confirmed by future research. The potential and actual impacts of financial inclusion need to be viewed against those of comparable interventions, such as graduation and livelihoods‐enhancement programmes.

We note that, fortunately, our findings regarding impact chime in with an emerging realism around microfinance, including in the donor community: recognising that erstwhile claims of transformative impact were unrealistic and that the hype for microfinance, particularly microcredit, was overblown. We welcome this newfound realism and wish to encourage it with the help of this review, in which we provide a systematic overview of the evidence as well as the areas of doubt in the evidence base. At the same time, we wished that going through all stages of the hype cycle—enthusiasm, inflated expectations, and disillusionment—had not been necessary in order to arrive here. And we must warn that we see a similar hype of strong claims emerging around the much more encompassing notion of financial inclusion, with the promise of marrying macro‐structural economic improvements with micro‐structural poverty relief. We found no evidence for the wider claims made for the beneficence of financial inclusion, as offering poor people a better service, or as having broader macro‐structural effects, being any truer than those once made for microfinance, in large part due to a lack of appropriate research at the meta‐study level. We strongly caution against repeating the hype cycle, this time around the idea of financial inclusion.

At the same time, we think it crucial to bear in mind that the alternative to financial inclusion is not to do “nothing,” but rather it is necessary to uncover what kinds of interventions work best for whom and where, and how best to deliver them. The policy and research space—and ultimately poor and low‐income people themselves—would benefit from a more open and clear‐sighted discussion on the many valid alternatives to financial inclusion programming and on how best to gain the necessary evidence to inform that discussion. To this end, our review also includes a brief examination of the impact evidence for graduation and livelihoods programmes.

In terms of evidence gaps, it is noteworthy that none of the meta‐studies we reviewed (high‐, medium‐ or low‐confidence) managed to assess debt levels or indebtedness patterns in depth as an outcome of financial inclusion. While we cannot comment on the reasons for the lack of attention paid to the issue, except that we are aware of it also being a blind spot of the underlying primary studies, we find this to be a glaring omission of the financial inclusion literature as a whole. We believe the political economy of research funding needs to shift such that researchers are enabled and encouraged to more rigorously explore the most important potential downsides and risks of development initiatives like financial inclusion. Furthermore, we found no evidence (among the high‐, medium‐ or low‐confidence meta‐studies) for the claim that financial inclusion interventions lead to macroeconomic development and subsequent improvements in the lives of the poor; this may be because the argument has only become prominent in recent years. There is also not much attention given (among the high‐, medium‐ or low‐confidence meta‐studies) to service/amenities‐related programmes such as water credit, sanitation loans, or loans for micro solar systems, especially the notion of “Green Microfinance” where microfinance is applied to promote environmental sustainability.

Moreover, given that the majority of financial inclusion effects we found in assessing the high‐ and medium‐confidence studies were at the early stages of the causal chain, there is a need for studies to better capture long‐term effects and demonstrate more meaningful impacts, especially at the final stages of the causal chain. The vast majority of the studies that our meta‐studies reviewed had a duration of 1–3 years. These studies are likelier to find changes in behaviours or attitudes rather than structural changes to people's poverty status, and it is not safe to assume that the latter will result from the former. The design of most studies underlying the meta‐studies that we reviewed has not been conducive to establishing whether short‐term or immediate outcomes (such as financial knowledge or entrepreneurial propensity) would translate into intermediate outcomes (such as savings accumulation or microenterprise income) and especially more distal, transformative outcomes (higher net worth or higher incomes). We would suggest that this also reflects a problem of the political economy of development research, with a combination of funder restrictions (favouring shorter timelines over multi‐year projects) and difficulty of gaining long‐term support from implementer organisations discouraging appropriate designs.

We have also encountered some important limitations of working at this level of systematisation, including: difficulties of assessing the reliability of the levels of evidence underlying ours; analysing effect sizes that are presented in standardised and indexed form, which often reveal little about the underlying measures used; the different ways in which data have been analysed and findings presented across very different types of meta‐studies; crude categories for intervention and outcome types, lumping together a highly diverse evidence base that muddies the waters further. Another problem we encountered was that the meta‐studies we reviewed, regardless of their own quality, often built on a relatively weak underlying base of underlying studies, making their findings fragile. To put it differently, combining a wide range of low quality studies into systematic reviews to aggregate their findings is risky, and perhaps analogous to the behaviour of financial institutions in the run‐up to the 2008 financial crisis, with pooling dubious individual assets (such as subprime mortgages and loans) into “triple‐A” structured financial products, with only seemingly better aggregate results.

Going forward, we would recommend that authors of primary studies and meta‐studies engage more critically with study quality and ensure better, more detailed reporting of the concepts, data and methods they used. At the systematic review of review level, more methods guidance (especially in terms of synthesis approaches) and clearer reporting standards that adapt the Cochrane (health‐focused) guidance to the social science and international development context would be helpful.

## 3 BACKGROUND

### 3.1 The problem, condition or issue

Financial inclusion is presently one of the most widely recognised areas of activity in international development. As of 2017, globally, about 1.7 billion adults were counted as “unbanked”, not having an account at a financial institution or through a mobile money provider, but 515 million adults worldwide opened an account between 2014 and 2017 (Demirgüc‐Kunt, Klapper, Singer, Ansar, & Hess, 2018 p. 2–4). Adults may be “unbanked” for reasons including unaffordability and inaccessibility of financial services, low quality, or choice. Financial inclusion refers to efforts to deliver affordable financial services—transactions, payments, savings, credit and insurance—to these people in a responsible and sustainable way. Financial exclusion is often blamed for inequalities (including in access to economic opportunities), a lack of security, and an exacerbated exposure to risk (Carbo, Gardener, & Molyneux, 2005, p. 5–7). The expectation underlying financial inclusion is that greater access to financial services will create poverty‐alleviating and empowering effects; or, according to the United Nations Secretary‐General's Special Advocate for Inclusive Finance for Development, have the effect of “transforming lives” (UNSGSA, 2017).

With financial inclusion, policymakers and donors hope that access to financial services (including credit, savings, insurance and money transfers) provided by a variety of financial service providers, of which MFIs are a subset, will allow poor and low‐income households in low‐ and middle‐income countries to enhance their welfare, grasp opportunities, mitigate shocks, and ultimately escape poverty, as well as advance macroeconomic development, which is also expected to benefit poor/low‐income households (Beck, Demirgüc‐Kunt, & Levine, 2007; World Bank, 2014). More recently, some donors have suggested behavioural changes (such as household spending decisions) to be desired outcomes of access to financial services, as well (Karlan, Ratan, & Zinman, 2014; World Bank, 2015). However, the present state of evidence leaves it insufficiently clear to what extent and for whom what benefits occur or do not occur (Demirgüc‐Kunt, Klapper, & Singer, 2017; Mader, 2016).

Systematic reviews and meta‐analyses (in short: meta‐studies, we often use the term “reviews” interchangeably with “meta‐studies” in the sections below) have sought to clarify the impacts from financial inclusion on poor people in low‐ and middle‐income countries, based on an array of different underlying studies which include quantitative and qualitative work based on long‐term and short‐term data. The bulk of these meta‐studies have been focused on microfinance, and many specifically on microcredit. The very different quality and approaches of these meta‐studies, and of the studies underlying them, however, pose a major challenge for policymakers, programme managers and practitioners in assessing the benefits and drawbacks of finance‐based approaches to poverty alleviation. Increasingly there is confusion about the impacts and a risk of “cherry picking” among different findings. Further, many meta‐studies are not taking into account what is missing from their primary studies, which would affect the understanding of the evidence, for example by not analysing or reporting gendered impacts. More recently, primary studies^1^ have also sought to understand the impacts of financial inclusion initiatives more broadly, especially regarding macro‐structural changes (Cull, Ehrbeck, & Holle, 2014; Demirgüç‐Kunt & Klapper, 2013), but the systematic review evidence has not yet progressed as far.

Our primary aim is to gain better clarity about the impacts of financial inclusion on the poor by systematically reviewing the existing systematic reviews and meta‐analyses (meta‐studies). Unlike most previous systematic reviews, which focused on microfinance interventions (or subsets thereof), we explicitly adopt a broader scope to review any available systematic review and or meta‐analysis evidence on financial inclusion as a whole field. Greater clarity through greater evidence systematisation is urgently needed given the strong focus on expanding access to financial services in the Sustainable Development Goals (SDGs), in particular SDG 1 on eradicating poverty^2^ and SDG 5 on achieving gender equality and women's empowerment,^3^ and in light of the risks that some forms of financial inclusion pose to vulnerable populations (Guérin, Morvant‐Roux, & Villarreal, 2013). In addition to this primary aim, we have three secondary sub‐objectives

- to better inform the decisions of development donors, policymakers and programme managers by establishing what is known and not known about the impacts, using a meta review methodology;
- to facilitate better research by assessing the strengths and weaknesses of existing systematic reviews and meta‐analyses, and suggesting pathways toward improved and common standards and methods, particularly with more explicit attention to gendered equity determinants and better use of qualitative studies;
- to understand better the political economy of knowledge, which may explain which questions are asked and why, what analysis used and why, and how results are interpreted.

### 3.2 The intervention

Financial inclusion is an umbrella term, which the World Bank Group defines as follows: “Financial inclusion means that individuals and businesses have access to useful and affordable financial products and services that meet their needs—transactions, payments, savings, credit and insurance—delivered in a responsible and sustainable way.”^4^

The field of interventions to bring about financial inclusion in low‐ and middle‐income countries is diverse and complex. It encompasses microfinance, as the best‐known intervention in this space, but increasingly extends well beyond it. *Microfinance* refers to the provision of financial services including loans, savings accounts, insurance (e.g., health, crop, life, credit life or default insurance), and money transfer services, specifically to poor and low‐income people in low‐ and middle‐income countries around the world who are not usually served by the regular banking sector, by *dedicated providers* who collectively identify as MFIs; these providers may range in size and type from small, local nonprofit NGOs to large commercial microfinance companies. *Financial inclusion interventions* refer to the range of broader efforts to expand financial systems to deliver financial services—loans, savings, insurance or payment services—to a wider client base, in particular poor and low‐income people in low‐ and middle‐income countries, that has not traditionally been served by the regular banking sector, by any range of formal service providers.^5^ These service providers commonly include MFIs in addition to commercial banks, nonbank financial companies, credit card companies, government programmes, cooperative banks, village savings and loan associations, some types of self‐help groups (SHGs), and also mobile network operators and fintech companies. In recent years, the delivery of financial services through digital means of service provision has been increasingly emphasised by governments, development funders and service providers themselves (Gabor & Brooks, 2017).

The financial services provided in the financial inclusion space are of four main types: credit, savings, insurance and payment services. The most commonly‐provided services within financial inclusion still are microcredit loans, made to about 211 million families worldwide (Microcredit Summit Campaign, 2015), with durations of around 12 months, which are repaid in weekly (and sometimes bi‐weekly or monthly) instalments, and are often guaranteed by group membership, small collateral items, or personal guarantors. Savings and insurance services are usually offered only in conjunction with loans—mixed (micro‐) finance—, but also sometimes independently. Particularly in South Asia, savings, credit, and other financial services are often delivered through community‐based savings groups (CBSGs), which include SHGs. Money transfers and mobile payments services (i.e., financial technologies, or fintech, that have the potential to disrupt established business models of the inclusive financial space by delivering financial services via digital platforms) are a relatively new area of activity, which is still under development in many countries, but has achieved scale in parts of East Africa and South Asia. In assessing financial inclusion, we thus face a multitude of services, providers, and users. Interventions for financial inclusion include of a diverse set of services orchestrated through various delivery mechanisms, ranging from small‐scale and community‐led initiatives to often very large scale government‐organised, donor‐backed or commercially‐driven programmes. The space of financial inclusion is changing rapidly, and the purpose of this systematic review of reviews^6^ is to assess evidence for the broader range of inclusive financial services increasingly being offered, as far as possible, including but going beyond (micro‐)credit. Below, in reporting outcomes, we differentiate between (micro‐)credit, (micro‐)savings, (micro‐)insurance, CBSGs and mixed microfinance (where it is unclear exactly which microfinancial services are provided, or where several are provided together).^7^

It is important to note that, while many financial inclusion services may be delivered separately or bundled by a given provider, in practice, households often combine them in a variety of ways, or even use services for different purposes, for instance using access to credit as a form of insurance. Hence, this renders an intervention‐focused systematic review of reviews artificially narrow, and instead calls for a synthesis of impacts by outcomes, while tracing any effects back to particular interventions or services as much as possible, and this is what we propose to do in this review.

### 3.3 How the intervention might work

The policy rationale behind financial inclusion activities is that the usage of financial services is expected to improve the lives of poor and low‐income people in low‐ or middle‐income countries (i.e., generate a positive impact). Our systematic review of reviews is theory‐based in the sense that it examines the evidence for and against the correctness of the theory of change underlying financial inclusion programming. The importance of developing and applying a theory of change—to clarify how “the intervention is expected to have its intended impact” (White, 2009, p. 274)—has been increasingly emphasised in recent years in impact evaluations and meta‐studies (cf., Maitrot & Niño‐Zarazúa, 2017). A theory of change serves to explain how activities are *expected* to produce a series of results that contribute to achieving intended impacts, by schematically explaining the causal links from programme inputs to ultimate (or higher‐order) outcomes. Using a theory of change or “logic model” allows us to link “programme inputs and activities to a chain of intended or observed outcomes, and then [use] this model to guide the evaluation” (Rogers, 2008, p. 30; White, 2009). In other words, the theory of change of financial inclusion should show how financial inclusion initiatives are expected to create desired positive changes for the target population, and thus to aid the interpretation of findings by clarifying differences between programme uptake, immediate effects, and more transformative impacts.

**Figure 1:**
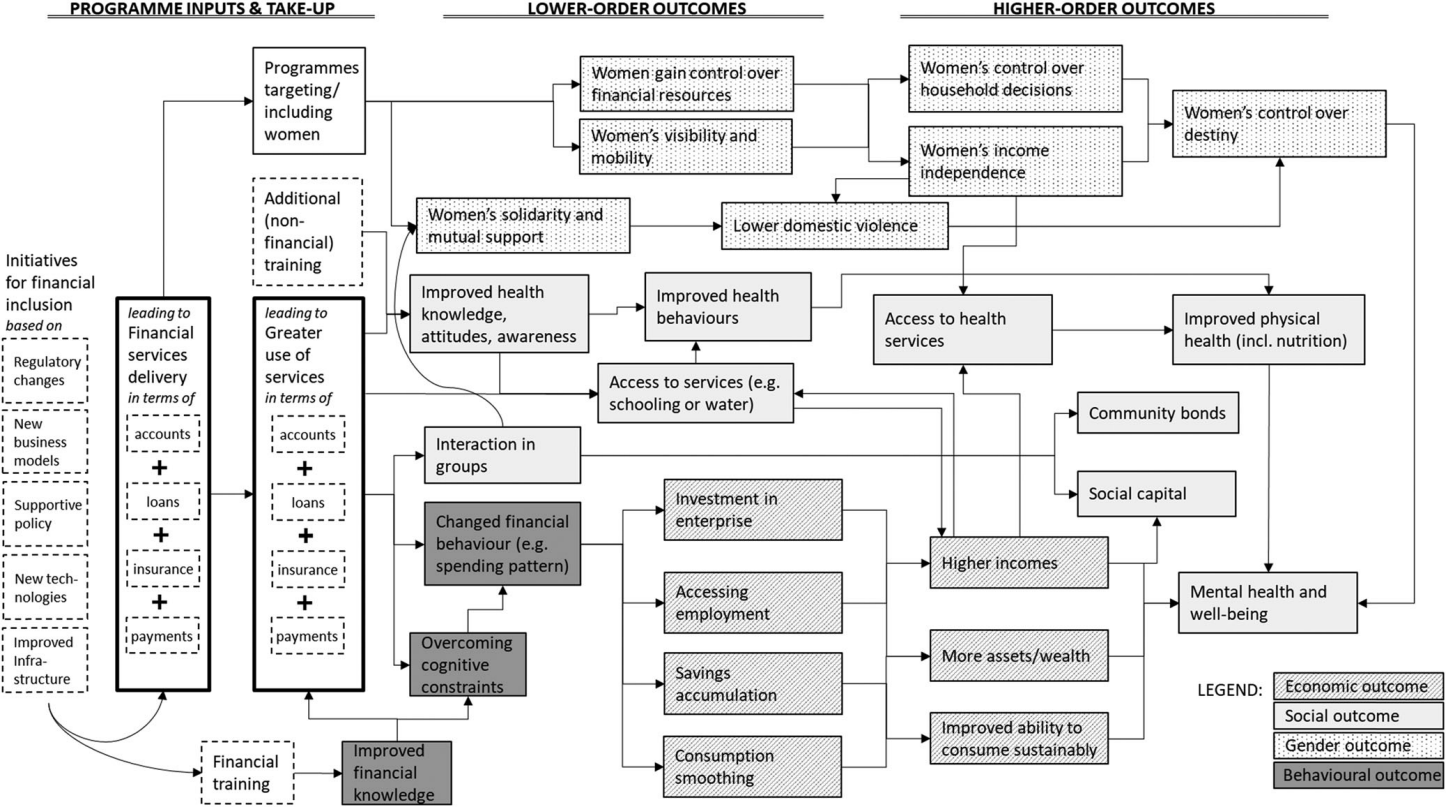
Financial inclusion impacts: Theory of change flow diagram. A macro‐structural outcome category is not shown, because its causal chain does not operate at a household level

Financial inclusion encompasses a wide range of intervention types and approaches, and numerous different types of intended outcomes and impacts have been suggested as part of its transformative impacts and as intermediary steps leading to them. Given this complexity, our theory of change must necessarily be abstract, simplified, and non‐exhaustive, highlighting main (or exemplary) channels of influence rather than all possible effects (and cross‐linkages between effects) of financial inclusion interventions, Figure 1 highlights the main theorised channels of influence (rather than all possible effects, backward linkages, cross‐linkages, or potential unintended consequences) of financial inclusion interventions, beginning with the possible drivers of enhanced financial service delivery. As shown in the left part of Figure 1, regulatory changes, the emergence of new business models and technologies, supportive policies, and improvements to (financial) infrastructures are expected^8^ to lead to a more inclusive offering of accounts (including savings accounts), credit, insurance and payments services (as well as financial training), which households in turn access and use (uptake).

Our representation of the theory of change then proceeds from the recognition that uptake is different within households, and that financial services are fungible within the household, such and that households use and combine them for very different ends (Collins, Morduch, Rutherford, & Ruthven, 2009). Thus, although in reporting findings we seek to distinguish as much as possible between the outcomes from different service types, in developing an encompassing theory of change for financial inclusion, we believe it would be counterproductive to focus on particular impacts as arising only from only particular financial services. We focus on establishing a set of causal chains from households’ uptake (usage of *any or several or all* of these financial services) to immediate changes (lower‐order outcomes) and from there to more transformative changes (higher‐order outcomes). In doing this, we distinguish between four outcome categories: economic, social, gender, and behavioural (distinguished in Figure 1 by different shading). Notably, these causal chains from financial inclusion to potential impacts of financial services usage on poverty are interdependent, as indicated by the cross‐connections in the figure. Most existing meta‐studies have focussed on individual parts of this broad theory of change, or only certain pathways within them.

#### 3.3.1 Economic

In theory, financial inclusion could lead to benefits for poor people through changes in their financial behaviours such that they use financial services to gain access to new income sources or enhance existing ones, to save money that they would otherwise spend or lose, to invest in assets, to sustainably consume more goods, or to cope with shocks. Specifically, credit might be used to create or expand a business that then makes a profit, or to gain access to a new (other) income‐earning opportunity, such as a job that requires travel. Credit or savings might also be used to mitigate a shock, invest in a household asset, or pay off a more expensive loan. Credit or savings can allow people to accumulate a lump sum for a large investment, cope with shocks, or simply to avoid more expensive credit. Lower‐order outcomes, that is, impacts found on outcomes early in the causal chain, would include the simple fact of having an enterprise (rather than none), increasing the size of one's enterprise, accessing new or better employment, accruing more savings, and having smoother consumption patterns (for instance no periods of hunger). Higher‐order outcomes that occur further along the causal chain (and which these lower‐order outcomes ought to lead to, in order to actually alleviate poverty) would include sustainably higher incomes and more assets or wealth (higher household net worth, net of debts). The ability to consume more goods sustainably (i.e., without over‐spending) is also a higher‐order outcome; however the sustainability of consumption is difficult to ascertain, because changes in consumption levels are might stem from positive causes (such as having more available income) or negative causes (such as higher costs or spending on credit).

#### 3.3.2 Social

Under the heading of “social outcomes” we collect the gamut of other beneficial outcomes that are not strictly behavioural, economic or gender‐related. We break these down further into three broad categories: health (physical health, nutrition, mental and psychological health), social‐relational (strengthening of social ties, community bonds), and access to beneficial services (such as water or schooling). In theory, financial inclusion might affect these in multiple different ways, again with lower‐order outcomes leading to higher‐order outcomes within each category.

- Health: Financial services, particularly when accompanied with training or awareness‐raising efforts, might positively affect health knowledge, attitudes or behaviours (lower‐order outcomes), which in turn may lead to improved health outcomes (higher‐order). Increased incomes, savings or spending capacity would also enable people to access to health services by making them more affordable, leading to better health outcomes (as a higher‐order outcome). Increased income independence and improved control over their own destiny for women could improve their health outcomes in particular. Reduced poverty or increased capabilities resulting initially access to financial services could also improve mental health and psychological well‐being (higher‐order).
- Social‐relational: In particular with forms of financial service delivery that lead to more regular and positive interaction in groups (lower‐order), clients’ social ties and social capital might be strengthened and community bonds be created (higher‐order). Reduced poverty at an individual level may also improve clients’ social capital, as they rise in the estimation of others (higher‐order).
- Services: reduced poverty, which may result from financial inclusion, would make households more able to pay for services such as schooling, water and sanitation (lower‐order outcomes), which in turn would lead to better health and economic outcomes (higher‐order outcomes). Financial products might also be used by clients directly to finance access to particular services or amenities, if they choose to do so; or financial services may be linked to the purchase or use of particular products, as with school savings accounts or sanitation loans. Financial service delivery might also include components of sensitisation, awareness or attitude‐change, to increase clients’ propensity to use (or pay for) particular amenities.

#### 3.3.3 Gender

Financial services may have very different impacts on women and men, particularly if they target women or at least are accessible for women. Many financial inclusion programmes (particularly microfinance and SHGs) have a history of targeting women and aiming to effect women's empowerment; some modes of digital financial services have also been claimed to have positive effects particularly for women by allowing them to save independently, despite not targeting women. In theory, financial services could affect gender relations in a number of complex and interrelated ways, which would be difficult to label as lower‐order or higher‐order.^9^ Through financial inclusion, women could gain control over financial resources and this may improve their implicit or explicit bargaining position within the household, including on matters such as family planning. Women's control over financial resources could allow them to create or access an independent source of income. As their women's independence improves, domestic violence could reduce. Leaving the home to access financial services or engage in business can make women more visible in the community and give them greater mobility, and women's participation in economic life outside the home may also lead to a broader sense of empowerment and control over destiny, all of which could improve their physical and mental health and well‐being. Furthermore, regular meetings of women could improve women's sense of solidarity and strengthen their mutual support, and some programmes have specific components of solidarity‐building or exposure to women's rights. However, with all these changes, it is important to note that they may contain ambivalences, for instance where women might not want to be more visible (as in some traditional societies) or when newfound independence leads to adverse reactions from men which could mitigate or undermine the benefits.

#### 3.3.4 Behavioural

It has been suggested, particularly by behavioural economists and recently the World Bank,^10^ that financial services, especially ones that contain particular modalities to affect users’ behaviour, lead to various potentially desirable cognitive capabilities and behavioural changes. In theory, changes in behaviours and cognitive capacities could come from several factors. First, changes in financial knowledge and abilities could come directly from directly being taught in financial literacy or education programmes (which are sometimes attached to financial service delivery, but which on their own we deemed beyond the scope of this review, as not being directly part of financial services and only training for readiness to use the latter) or through experience gained over time in using money and financial services. Financial products might also, as a by‐product of their usage, change users’ money‐usage patterns over time, for instance leading to higher propensities to save, more investment in business, or less spending on particular goods such as “temptation goods” (Banerjee, Duflo, Glennerster, & Kinnan, 2015). It has also been suggested that specially designed financial products could help poor people overcome behavioural or cognitive constraints or attitudes that the designers of these products believe worsen poverty and hold people in poverty, as for instance if “commitment” savings devices commit people to longer‐term goals rather than giving in to possible biases toward present enjoyment. We treat all behavioural outcomes as lower‐order outcomes, because they ought not to be seen as ends in themselves, and merely indicate a potential for poverty‐alleviating effects to happen further along the causal chain.

#### 3.3.5 Macro‐structural

Lastly, in recent years, it has been suggested that inclusive financial sectors are conducive to macroeconomic development, from which poor and low‐income people in turn would benefit (Cull, Demirgüç‐Kunt, & Morduch, 2013; World Bank, 2014). This outcome category is different, as the mechanisms of impact operate at the macro, rather than household, level; we have therefore not included it in Figure 1, which graphically presents the theory of change at the household level (however, our review still aims to capture any evidence on these types of effects). Some economic literature suggests more inclusive financial sector development could drive macroeconomic growth by mobilising savings and investments in the productive sector, and reducing information, contracting and transaction costs across the economy, leading to efficiency gains, which lead to growth; poverty alleviation would result if poor people benefit from subsequent economic growth, for instance through higher demand for their skills. It has also been suggested that financial sector development could reduce economic inequality indirectly (through forms of growth that lower inequality) or through enabling lower‐income individuals to use finance to invest in accumulating human capital (Beck et al., 2007; Jalilian & Kirkpatrick, 2005).

Finally, while this is not part of a theory of change—which serves only to clarify how “the intervention is expected to have its intended impact” (White, 2009, p. 274)—it is important to note that, for all outcome categories, the possibility of unintended negative consequences and adverse effects (on average, or for parts of the population, i.e., mixed impacts) also exists. There is no reason to assume a priori that the impacts of financial inclusion will be positive or significant. Some past evidence has suggested more inclusive financial service provision may also have negative impacts such as worsened impoverishment (Mosley, 2001), financial and emotional stress (Ashta, Khan, & Otto, 2015), debt traps and permanent indebtedness (Guérin et al., 2013; Schicks, 2010), gender‐based violence and women's disempowerment (Rahman, 1999), and undereconomic development and greater social inequality (Bateman, 2010; Sandberg, 2012). Our systematic review of reviews captures and accounts for any findings of negative impacts, including mixed ones.

### 3.4 Why it is important to do the review

While a large number of methodologically robust studies have systematically synthesised evidence on microfinance, the same cannot yet be said for financial inclusion more broadly. Some donor agencies, especially the World Bank, have carried out primary studies on financial inclusion of various types including microfinance facility to justify why financial inclusion policy matters, how it matters, and what it means to policymaking (cf. Cull et al., 2014; Demirgüç‐Kunt & Klapper, 2013; Demirgüc‐Kunt et al., 2017; World Bank, 2014). But the existing research syntheses on financial inclusion (beyond microfinance) have been unsystematic in their approach.

Polanin, Maynard, and Dell (2017) provide four reasons for why systematic reviews of reviews are important:

1. They can contribute to the knowledge base going beyond what systematic reviews and meta‐analyses report examining trends over time and thus be particularly useful to policymakers, practitioners and researchers.
2. Where many systematic reviews on a given topic exist reporting discordant views, systematic reviews of reviews can be particularly useful to make sense of these diverging conclusions by comparing and contrasting the results of multiple systematic reviews.
3. They have the potential to conduct network meta‐analysis (Ioannidis, 2009) to allow comparisons of multiple treatment and control groups.
4. They can point out when systematic reviews need updating again.

Finally, it is worth noting that systematic reviews of reviews also have a role to play in translating knowledge into policy impact (Whitty, 2015).

In the context of financial inclusion, without robust evidence that financial services generate significant and meaningful—ideally: transformative—impacts in poor people's lives, financial inclusion efforts would lack a clear justification in developmental or social policy terms. This can be said without pre‐judging the evidence. However, the existing meta‐studies (which have focused on microfinance rather than financial inclusion broadly‐defined) have generated few strong or unambiguous results, suggesting that the improvements in poor people's lives that accrue from financial inclusion are relatively small or manifest mainly as intermediary impacts—changes in behaviours and spending patterns, rather than changes in incomes or well‐being—, at least in the shorter term. Presently, too little is known across different meta‐studies with different approaches, and a systematic review of reviews helps generate a clearer picture.

Existing meta‐studies have reviewed primary studies of many different types of financial services. A substantial number of systematic reviews, meta‐analyses and research syntheses on financial inclusion and closely connected topics exist. However, the focus of the bulk of studies (in keeping with the activity focus of the financial inclusion sector) has been on credit and credit‐type (e.g., leasing) services, particularly those provided by MFIs. The evidence based on other services is smaller but growing rapidly, particularly in the area of mobile service provision and fintech for development.

The existing meta‐studies have followed diverse approaches. Some of the systematic reviews (or meta‐studies) are fairly broad, aiming to cover the whole microfinance spectrum (e.g., Duvendack et al., 2011). Others cover specific interventions, such as microcredit (e.g., Vaessen et al., 2014), formal banking services (Pande, Cole, Sivasankaran, Bastian, & Wendel, 2012), microenterprise (e.g., Grimm & Paffhausen, 2015), microsavings and microleasing (Stewart et al., 2012) and microinsurance (Cole, Bastian, Vyas, Wendel, & Stein, 2012). Some systematic reviews focus on particular populations, such as Sub‐Saharan African recipients (e.g., Stewart, van Rooyen, Dickson, Majoro, & de Wet, 2010), particular methods of providing financial services, such as SHGs (e.g., Brody et al., 2015) or particular outcomes, such as health (e.g., Leatherman, Metcalfe, Geissler, & Dunford, 2012) or empowerment (Brody et al., 2015; Vaessen et al., 2014). The systematic reviews also differ by focus, many covering effectiveness evidence, but others incorporating participant views (e.g., Brody et al., 2015; Peters, Lockwood, Munn, Moola, & Mishra, 2016) and barriers or enablers of uptake and effectiveness (e.g., Panda et al., 2016) including innovations in information and communications technology (e.g., Gurman, Rubin, & Roess, 2012; Jennings & Gagliardi, 2013; Lee et al., 2016; Sondaal et al., 2015).

The existing meta‐studies use a range of methodologies to synthesise the evidence, including theory‐based approaches, narrative syntheses and statistical meta‐analyses. Many of them have not been conducted to standards that would support a “high confidence” rating (as discussed below in the Section 5); not all meta‐studies that have impacted policy discussions have used a systematic methodology (Bauchet, Marshall, Starita, Thomas, & Yalouris, 2011; Beck, 2015; Odell, 2010). In addition, the majority of meta‐studies are available in technical reports where there is no transparent decision rule for determining implications of the findings, including critical appraisal and strength of evidence tools like GRADE assessment (Guyatt et al., 2013) and user‐friendly presentation of results (e.g., translating standardised effect sizes into metrics commonly used by decision makers). There is no overall synthesis of the implications for policy, programming, practice and research for the sector from this body of synthesised evidence.

Our systematic review of reviews brings a systematic overview about what is known about what aspects of financial inclusion (what, where, how?) and which gaps and white spaces remain in terms of knowledge about the impacts. Rather than visualise these gaps and white spaces, we describe them narratively, focusing on a range of parameters (e.g., intervention type, outcome measures, geographical focus, etc.), which in turn inform our synthesis approach which, among other things, also focuses on the following unresolved questions (discussed in more depth in the section outlining our approach to data synthesis):

- What can explain which questions are asked in some systematic reviews and meta‐studies about the impact of financial inclusion, and which ones not?
- What can explain different interpretations of results from existing studies?

A clear mapping of knowledge gaps allows policy‐oriented research funders to better direct funds towards addressing the gaps, and the systematic reviewing of known impacts allows policymakers to focus their efforts on those interventions that are known to work best, on where they work best, or to improve or otherwise eschew them. Our stakeholder engagement strategy includes a non‐technical report (for 3ie), dissemination events, and work with our advisory board of policy‐ and research‐related stakeholders.

## 4 OBJECTIVES

### 4.1 The problem, condition or issue

The objective of this systematic review of reviews is to systematically collect and appraise the existing systematic reviews and meta‐analyses of financial inclusion impacts, analyse the strength of the methods used, synthesise the findings from those systematic reviews and meta‐analyses, and report implications for policy, programming, practice and further research.

Systematic reviews of reviews have been undertaken in other sectors for which evidence is widely available, especially health (Becker & Oxman, 2008) and more recently education (Polanin et al., 2017), but they are nonexistent in international development, and thus this study represents a pioneering effort to address a notable evidence gap.^11^ It provides the opportunity to develop and pilot an evidence synthesis approach in a sector where there is a large body of evidence of variable quality, but systematic appraisal and synthesis of the body of systematic reviews and meta‐analyses is still lacking. Polanin et al. (2017) provide useful guidance on how best to conduct such systematic reviews of reviews; they point towards methodological challenges of such reviews and suggest ways forward to improving them.

This study critically reviews existing approaches to systematic reviews of reviews with a view to further developing systematic review of review methods, and it aims to answer the following questions to gain better clarity about financial inclusion impacts:

- Impacts
  ○ What is known from existing meta‐studies about the (social, economic, and behavioural) poverty impacts of different types of inclusive financial services (e.g., credit, savings, insurance and money transfers), regardless of provider, on poor and low‐income people in low‐ and middle‐income countries?^12^ This includes the poverty impacts from macroeconomic development, to the extent that it results from financial inclusion.^13^
  ○ What is known from existing meta‐studies about the gendered impacts of different types of financial inclusion activity (e.g., credit, savings, insurance and money transfers)—in other words, what does the evidence tell us about how gendered participation affects interventions’ effects, and about whether or not (and in what ways) financial services empower women in low‐ and middle‐income countries?
  ○ What is known from existing meta‐studies about the reasons for financial services uptake, or other participant views about the financial services on offer?
- Methodology
  ○ Including using a gender and equity lens, what methods and standards have meta‐studies used to draw conclusions from the studies they reviewed?
  ○ What difference does the choice of methods and standards make to the results?
  ○ How could the methods and standards be improved in order to draw more robust and reliable conclusions via meta‐studies?

## 5 METHODS

### 5.1 Criteria for considering studies for this review^14^

#### 5.1.1 Types of reviews

We sought to include all studies of sufficient quality (we discuss our understanding of “sufficient quality” in Section 5.3.3. as outlined in Table 2 but also in Appendix 7) which self‐identified as systematic reviews and or meta‐analyses of the impacts of financial inclusion (including, but not limited to, microfinance). These, in turn, have focused on synthesising quantitative, qualitative and or mixed methods evidence. According to the Campbell Collaboration,

A systematic review summarises the best available evidence on a specific question using transparent procedures to locate, evaluate, and integrate the findings of relevant research (The Campbell Collaboration, 2014, p. 6).

In the Cochrane Handbook (Higgins & Green 2011), the following definition of systematic reviews is outlined which we adopted:

A systematic review attempts to collate all empirical evidence that fits pre‐specified eligibility criteria in order to answer a specific research question. It uses explicit, systematic methods that are selected with a view to minimising bias, thus providing more reliable findings from which conclusions can be drawn and decisions made (Section 1.2 in Higgins & Green, 2011).

Higgins and Green (+++2011) specify the key elements that a systematic review should contain:

- A set of clearly stated objectives and pre‐defined eligibility criteria
- A methodology that is clearly defined allowing reproducibility
- A search strategy that allows the identification of studies meeting the pre‐defined eligibility criteria
- A critical appraisal of included studies
- A systematic synthesis, in many cases systematic reviews adopt a meta‐analytical approach which is a statistical method to synthesise the results of primary studies included in a systematic review

To identify meta‐analyses, we adopted the definition of the Cochrane Handbook (Higgins & Green, 2011):

“Meta‐analysis [is] the statistical combination of results from two or more separate studies” to produce an overall statistic with the aim to provide a precise estimate of the effects of an intervention (Section 9.1.2 in Higgins & Green, 2011).

It should be noted that not every systematic review automatically contains a meta‐analysis, for example, if primary studies are too heterogeneous in terms of study designs, conceptual framings and or outcomes, then a meta‐analysis may not be appropriate. Furthermore, occasionally meta‐analyses are published separately without drawing on the broader systematic review they may have been originated from.

We exclude any evidence that did not meet the definitions we outlined above.

#### 5.1.2 Types of participants

The scopes of the meta‐studies we include are diverse (different questions are often addressed and a range of linked interventions are examined, such as credit, savings, insurance, leasing, money transfers etc.) but there is considerable overlap in terms of their population of interest. Almost all focus on the impacts of financial inclusion on poor households based in low‐ or middle‐income countries (using the World Bank definition^15^). In other words, our population is the population of participants in inclusive finance activities that are conducted in low‐ and middle‐income countries. Where meta‐studies include evidence from high‐income countries, we would have only considered the findings that were presented for low‐ and middle‐income countries, but we did not find any such studies to include. We also included meta‐studies covering particular regions within low‐ and middle‐income countries, for example, Sub‐Saharan Africa or fragile and conflict‐affected areas.

At the primary study level, our population of interest would be participants taking part in inclusive finance activities in low‐ and middle‐income countries.

#### 5.1.3 Types of interventions

In this systematic review of reviews, we include all meta‐studies that address at least one or more types of intervention for financial inclusion, as described above. In the majority, the interventions are one or more sub‐categories of microfinance: credit, savings, insurance, leasing, and/or money transfers. However, our search strategy explicitly targets the broader range of inclusive finance activities, such as mobile monies, mobile payments systems, index insurance or savings promotion. For our purposes, to warrant inclusion of the systematic review or meta‐analysis, the reviewed intervention must have at least one financial service as an essential element of the intervention—for instance, not all systematic reviews of mhealth interventions would qualify for inclusion, but systematic reviews of mhealth interventions that required participants to purchase an insurance service would. The key is that the reviewed intervention must be fundamentally a financial service directed at poor and low‐income people, for it to qualify as a review of financial inclusion impacts.

At the primary study level, our intervention of interest would be interventions that address at least one or more types of financial inclusion interventions.

#### 5.1.4 Types of outcome measures

Existing meta‐studies of financial inclusion typically examine a wide range of poverty indicators (including income, assets, expenditure, personal networks, gender/empowerment, well‐being, health, etc.). In this systematic review of reviews, we include all meta‐studies that address at least one or more of these domains. We group the indicators in three categories of impacts: social, economic, or behavioural. We do not distinguish between primary or secondary outcomes but consider all outcome measures.

Our systematic review of reviews also assesses the evidence for outcomes early along the causal chain; most importantly rates of uptake, and then investment in productive activity, human capital accumulation, improved money management, savings accumulation, risk/shock management, health and nutrition spending, and women's economic activity. These might be enablers of improvements on poverty indicators further along the causal chain (over a longer term) even if, importantly, should not themselves be taken as evidence of impact in terms of poverty alleviation.

At the primary study level, our outcomes of interest would be outcomes that address at least one or more of the poverty domains described above.

#### 5.1.5 Timeframe

The first systematic reviews engaging with financial inclusion issues (Duvendack et al., 2011; Stewart et al., 2010) indicated that no systematic reviews existed prior to their reviews. The primary studies these two systematic reviews included date back to the late 1990s reporting on data that was collected in the early 1990s—this coincides with rigorous impact evaluations of financial inclusion (especially microfinance) becoming more mainstream. Hence, our searches are limited to 2010 onwards. However, to ensure that we are not excluding any relevant studies on date, we adopted a snowballing approach (as outlined below). In other words, any relevant meta‐studies published before 2010 would have been picked up through the snowballing procedure.

#### 5.1.6 Language

No restriction was placed on language of papers.

We did not need to make any changes to the eligibility criteria set out in this section during the course of the search and screening process *(relates to MECIR checklist, item 13*).

Evidence is included irrespective of its publication status (*relates to MECIR checklist, item 12*).

### 5.2 Search methods for identification of studies

We adopted a multi‐pronged search strategy which was informed by Kugley et al. (2016) and that explores bibliographic databases to identify published literature, institutional websites for published and unpublished literature, and back‐referencing from recent systematic reviews to ensure additional sources are identified.

#### 5.2.1 Electronic searches

We searched the following bibliographic databases:

- Business Source Premier (EBSCO)
- Academic Search Complete (EBSCO)
- EconLit—Via EBSCO Discovery Service
- RePEc—Via EBSCO Discovery Service
- World Bank e‐Library—Via EBSCO Discovery Service
- Scopus (Elsevier)
- Web of Science

#### 5.2.2 Searching other resources

The following institutional websites were searched:

*Financial inclusion‐specific institutions and web portals*:

- CGAP: www.cgap.org
- Microbanking Bulletin: www.themix.org
- Microfinance Gateway: www.microfinancegateway.org
- Microfinance Network: www.mfnetwork.org
- SEEP: http://www.seepnetwork.org
- Grameen Foundation
- BRAC Research and Evaluation Division
- Alliance for Financial Inclusion
- Accion Centre for Financial Inclusion.

*Multilateral and bilateral and non‐governmental donor organisations*:

- World Bank (WB e‐library was searched within EBSCO's Discovery Service but will also be searched and screened online via the World Bank's website)
- African Development Bank
- Asian Development Bank
- Inter‐American Development Bank
- DFID—R4D website
- USAID.

*Research institutions and research networks*:

- Centre for Global Development
- J‐PAL
- 3ie databases on systematic reviews
- ELDIS
- SSRN
- ResearchGate
- Academia.edu

After completing the screening process, we ran citation searches on included meta‐studies in Google Scholar, Scopus and Web of Science to identify more recent systematic reviews and or meta‐analyses not retrieved in database searches.

We piloted our key search terms (see Appendix 1 for full search strategies) and ran preliminary searches in EconLit (EBSCO) (510 hits), Scopus (1035 hits), RePEc (EBSCO) (238 hits), Academic Search Complete (EBSCO) (366 hits), and Web of Science (2014 hits). Search strategies were constructed using both textwords (title/abstracts) and where available index terms. Each strategy consisted of 3 parts—Intervention (financial inclusion, microfinance and other relevant terms), Study design (adapted from 3ie's search filter for its systematic review database), and LMICs (adapted from the Cochrane EPOC Group's LMICs filter based on World Bank definition of LMICs). We adjusted our search strategy for each database and web source. No restriction was placed on language of papers but all searches were limited to 2010 onwards (rationale provided above). We adopted a snowballing (also called reference harvesting) approach to ensure we have not missed any key systematic reviews and or meta‐analyses. We also consulted our advisory board to get their views on the sample of included studies and highlight any omissions. We ensured that our searches for all relevant databases were up to date, that is, they were updated within 12 months before publication of our study. In addition, we approached leading authors working on financial inclusion topics to double check that we are not missing out on any relevant ongoing studies.

### 5.3 Data collection and analysis

#### 5.3.1 Selection of studies

Two research assistants (RAs) screened all titles and abstracts of the studies identified by the academic and grey literature searches. Any disagreements were discussed and reconciled. The two review authors (MD and PM) independently reviewed each meta‐study marked for inclusion by the RAs to confirm the inclusion decision. Full texts were obtained and screened when a decision could not be made based on title and abstract screening. Disagreements were resolved by discussion or by involving a third party (e.g., a member of the advisory board) if a consensus could not be reached.

A PRISMA ﬂow diagram is presented in the results section (below) to summarise the study selection process and a table with excluded studies along with the reasons for exclusion is included in Appendix 5—see results section for more in depth discussions.

#### 5.3.2 Data extraction and management

Data were extracted by three RAs using the KoBo Toolbox^16^ which allowed conversion to an Excel spreadsheet. The extracted data were independently checked by the two review authors (MD, PM). In case of disagreements, they were resolved by discussion. The original authors of included systematic reviews and meta‐analyses were contacted where data were missing.

We extracted data on the following areas (for details see Table 1 below which was informed by Snilstveit et al., 2014):

1. Context
2. Type of intervention
3. Type of review, design and methods used
4. Outcome measures
5. Quality assessment
6. Study results and findings

**Table 1:** Data extraction form (template)

Data extraction items	Details
1. Context	• Source
	• Author
	• Publication year
	• Geographical focus (e.g., continent, countries and regions)
	• Funding source
2. Type of intervention	• Details of the population as discussed in the reviews (e.g., household, individual, enterprise; type of finance user, i.e., multiple borrower/saver, repeat borrower/saver; gender or other person characteristics, e.g., women focus or youth focus)
	• Broad category—type of product/service offered, ensure intervention has at least one essential financial service element
	• Detailed sub‐category of product (e.g., credit to existing businesses only, group savings account, etc.)
	• Comparator, i.e., comparing against nothing at all or against the next best alternative
	• Duration of intervention (e.g., length of exposure to intervention)
	• Modality of intervention—group vs individualLocation of intervention—urban/rural
	• Focus on women only (yes/no)
3. Type of review; design and methods	• Research question and review objectives—list actual question, plus clearly stated (yes/no)
	• Inclusion criteria—clearly stated (yes/no)
	• Search methods—e.g., number of databases, dates of search provided, search strategy/key words provided, additional search methods reported, any search restrictions (by language, timeframe?)
	• Study selection methods—clearly reported (yes/no), independent screening, full text review, consensus procedure for agreements
	• Number of included studies
	• Types of included studies
	• Types of data extraction methods—clearly reported (yes/no), independent screening
	• Types of data synthesis approaches (quantitative/qualitative)
	• Subgroup analysis conducted (yes/no)
	• Discussion of publication bias (yes/no)
4. Outcome measures	• Outcome definition, i.e., type of outcome measure to be grouped by social, economic, behavioural
	• Unit of measurement (e.g., at household or individual level, composition of empowerment indices)
5. Quality assessment	• Quality of review methods, their use and application—to be assessed using data extracted as part of “3. Type of review; design and methods” which will feed into AMSTAR rating
	• GRADE rating provided (yes/no)
	• Name of other quality assessment tools and their quality scores
	• Researcher bias/Conflict of interest
6. Study results and findings	• For each outcome:
	○ Sample size
	○ Type of effect size
	○ Magnitude and direction of effect size, if reported, to allow comparison across included studies

We extracted the most detailed data (also numerical data if it was available) to allow similar analyses of included studies.

We extracted information at the systematic review level. However, for systematic reviews classified as high and medium confidence, when necessary, we also extracted information at the primary study level on, for example, especially individual programme design, quality, and so forth.

##### 5.3.2.1 Criteria for determination of independent reviews (see MECIR checklist, items 40 and 42)

Some of the meta‐studies in our study sample have been published in multiple places, for instance as a Campbell systematic review but also as a peer‐reviewed journal article (e.g., Vaessen et al., 2014). Or they have been published on DFID's R4D website as well as a peer‐reviewed journal article (e.g., Stewart et al., 2012). Where this is the case, we treated them as duplicate reviews and extracted data from the most comprehensive version. Where we identified multiple versions of the same systematic review or meta‐analysis, we only included the latest updated version.

An issue that remains after removing duplicate systematic reviews and meta‐analyses is overlap. In our sample of included meta‐studies, we find reviews that included some of the same primary studies. One way to address overlap is to present a matrix (see Polanin et al., 2017) that includes all primary studies captured in the systematic reviews with a high and medium conference rating, which allows us to understand the extent of overlap, that is, which primary studies were included in which one of the high confidence systematic reviews in our study sample. A more detailed discussion on this can be found in the “Results” section.

#### 5.3.3 Assessment of risk of bias in included studies

##### 5.3.3.1 Assessment of methodological quality of included reviews (see MECIR checklist, items 52, 53 and 54)

The risk of bias of the included meta‐studies was assessed using the 3ie critical appraisal checklist,^17^ which is a variation of the checklist developed by the Specialist Unit for Review Evidence (SURE) in 2013. The objective of the original SURE^18^ checklist was to allow a critical appraisal to ensure that minimum levels of methodological rigour are met across included meta‐studies. We explored and added extensions to the 3ie checklist in collaboration with 3ie and added a critical appraisal component that captures the explicit use of theory in meta‐studies and to what extent an analysis of the causal chain has been undertaken; we discuss this in depth in Section 6.

Furthermore, to corroborate the findings of the 3ie critical appraisal checklist, we also employed the “A MeaSurement Tool to Assess systematic Reviews” (AMSTAR 2) developed by Shea et al. (2017), which is often used in the context of Cochrane overview studies. AMSTAR 2 is building on the original AMSTAR tool developed by Shea et al. (2007). It has 16 criteria^19^ and each is given a rating: “yes”, “partial yes” or “no”, allowing the user to make a broad assessment of the quality of the included meta‐studies. Table 2 below summarises the key assessment criteria of both the 3ie checklist and the AMSTAR 2 tool to clarify on what basis reviews were classified as low or medium/high confidence studies.

**Table 2:** Overview of the critical appraisal tools’ main quality assessment criteriaPossible result classes

3ie critical appraisal checklist	A MeaSurement Tools to Assess systematic Reviews (AMSTAR 2)
• Inclusion criteria reported	• Research questions and inclusion criteria reported with PICO (Population, Intervention, Comparator, Outcome)
• Reasonably comprehensive search strategy	• Review methods established prior to review; deviations from protocol reported
• Appropriate review time period	• Selection of included study designs explained
• Bias in selection of articles avoided	• Comprehensive literature search strategy used
• Characteristics and results of included studies reliably reported	• Study selection performed in duplicate
• Clear methods of analysis, including for calculating effect sizes	• Excluded studies listed and justified
• Extent of heterogeneity discussed	• Included studies described in adequate detail
• Findings of relevant studies appropriately combined relative to the question and available data	• Satisfactory technique used for assessing risk of bias
• Evidence appropriately reported	• Sources of funding of the included studies reported
• Assessment of factors explaining differences in results	• If meta‐analysis: appropriate methods used for statistical combination of results
• Consideration of aspects that may lead to questionable results	• If meta‐analysis: impact of risk of bias considered
• Consideration of mitigating factors for reliability	• Consideration of mitigating factors for reliability
• Use of programme theory of change^a^	• Risk of bias considered in interpretation and discussion of results
• Qualitative evidence incorporated in theory design^a^	• Heterogeneity discussed and explained
• Outcomes analysed along causal chain^a^	• If quantitative synthesis: publication bias considered
• Qualitative evidence incorporated in analysis^a^	• Conflicts of interest and funding for the review reported
• Qualitative evidence incorporated in other aspects^a^	
• Findings from quantitative and qualitative evidence integrated^a^	
• Quantitative and qualitative evidence integrated in conclusions and implications^a^	
Possible result classes:	
Low confidence	Critically low quality
Medium confidence	Low quality
High confidence	Moderate quality
	High quality

^a^ Criteria to capture use of theory and causal chain analysis, added after discussions with 3ie. See Appendix 7 for full versions of both quality appraisal tools.

We classed as medium‐low or medium‐confidence any meta‐studies that were classed either as at least “moderate quality” using the AMSTAR 2 tool or “medium confidence” using the adapted 3ie checklist. We classed as medium‐high or high‐confidence those meta‐studies that were classed as “high quality” or “high confidence” by at least one of the tools. We excluded from the final in‐depth analysis any studies that were classed lower than medium‐low confidence, that is, were not classed as at least “moderate” or “medium” by either of the tools. Instead of “quality”, throughout this review we use the term “confidence”, to highlight clearly distinction between the different levels of confidence in the absence of bias that we can have in the included studies.

The 3ie critical appraisal checklist and AMSTAR 2 tool were applied independently by the RAs and both review authors (MD, PM), and disagreements were resolved by discussion or by involving a third party (e.g., a member of the advisory board) if a consensus could not be reached.

##### 5.3.3.2 Assessment of the quality of the evidence in reviews

We attempted to extract GRADE ratings from each included meta‐study to assess the quality of the evidence. However, all except one of the included reviews adopted quality assessment approaches other than GRADE. Where this was the case, we reported the tool that was used and recorded its overall quality score, if one was given. We adapted the GRADE criteria related to risk of bias, inconsistency, imprecision, indirectness and publication bias (Guyatt et al., 2008)^20^ to suit the purpose of our systematic review of reviews. We employed revised GRADE criteria only for the meta‐studies that achieved a medium or high confidence rating. This work was conducted independently by one RA with involvement of both review authors (MD, PM), and disagreements were resolved by discussion or where necessary by involving a third party (i.e. a member of the advisory board).

#### 5.3.4 Data synthesis

The meta‐studies we included have included primary studies that employed quantitative, qualitative and mixed methods approaches. Hence, many of the meta‐studies in our study sample have adopted a narrative synthesis approach to deal with the methodological diversity of their included primary studies (e.g., Duvendack et al., 2011; Stewart et al., 2010, 2012). In some cases, however, meta‐analysis was feasible and was the preferred synthesis approach (e.g., Awaworyi, 2014; Lee et al., 2016; Yang & Stanley, 2013). In very few cases, a combination of qualitative and quantitative synthesis approaches was found (e.g., Vaessen et al., 2014).

Having reviewed the various synthesis methods set out by Barnett‐Page and Thomas (2009), we decided to adopt a narrative synthesis approach, as this accommodates both quantitative and qualitative information and is thus best suited for the diversity of the studies we included.

##### 5.3.4.1 Quantitative information

Some of the systematic reviews in our sample have taken a statistical meta‐analytical approach; where this was the case, we reported the average effects sizes for all outcomes for all medium‐ and high‐confidence reviews.^21^ We refrained from calculating the weighted average pooled effect sizes due to the high levels of heterogeneity in our sample of included studies. We are dealing with two levels of heterogeneity, at the primary study level—that is, studies included in each systematic review are highly heterogeneous—but also at the systematic review level where heterogeneity dominates which causes additional problems in terms of clustering interventions and outcomes in meaningful ways. In the attempt to ensure comparability across studies, we have translated all effect sizes into common metrics, using Polanin and Snilstveit (2016).

##### 5.3.4.2 Qualitative information

The majority of the included systematic reviews have adopted a theory‐based narrative synthesis approach. We present our findings according to the statistical information available in each systematic review, which is often a textual commentary. This commentary is enhanced by drawing on summary tables and figures using frequencies and percentages to describe and summarise the evidence we collected from the included reviews (see Smith, Devane, Begley, & Clarke, 2011 for suggestions for summary tables). Where possible, we also report findings in metrics of effect sizes and 95% confidence intervals, which occasionally requires the use of standard formulae to translate between effect sizes (e.g., see Sánchez‐Meca, Marín‐Martínez, & Chacón‐Moscoso, 2003 for guidance). As mentioned above, we would like to stress that, while we explored this reporting approach, we found it often had limited usefulness due to the very high levels of heterogeneity in a very small sample of medium‐ and high‐confidence meta‐analyses.

Baker, Costello, Dobbins, and Waters (2014) argue that the emphasis of systematic reviews of reviews should be on the presentation of the results and conclusions of the included reviews in accordance with their overall objectives. With this in mind, we organise our description of studies and synthesis by data extraction areas with a focus on outcome measures (as outlined above):

1. Context
2. Type of intervention
3. Type of review, design and methods used
4. Outcome measures
5. Quality assessment
6. Study results and findings.

The findings from our theory‐based mixed methods synthesis approach inform the conclusions of this study; we do not stray beyond the studies included in this review when discussing the implications for research and practice.

#### 5.3.5 Subgroup analysis and investigation of heterogeneity

We attempted to report sub‐group analyses, adapting the PROGRESS‐Plus checklist^22^ which was originally developed for Cochrane reviews focusing on health equity to enhance our understanding of impact heterogeneity, that is, impacts of certain elements of financial inclusion interventions may differ by gender, ethnic background, poverty level, and so forth. Thus, reporting sub‐group analyses would allow us to comprehend which interventions (or elements thereof) may or may not be effective in relation to certain sub‐groups in the population. However, it was very difficult to report any sub‐group analyses and further unpack these drivers of heterogeneity as the reviews we included often did not provide further disaggregated information, they rather created broad categories lumping together a range of diverse outcomes and intervention types as a way to deal with high levels of heterogeneity.

#### 5.3.6 Sensitivity analysis

Where possible, we stratify the included systematic reviews by quality, that is, confidence, rating (high, medium and low confidence) and explore whether the types of interventions or the number and/or types of the underlying primary studies play a role. We provide descriptive information on these topics for selected key outcomes.

## 6 RESULTS

### 6.1 Description of studies

#### 6.1.1 Results of the search

We initially identified 4,611 records from searching seven bibliographic databases. An additional 133 records were identified by trawling through websites of financial inclusion‐specific institutions and research networks. Sophisticated search terms trialled during the protocol stage (see Appendix 1 for details) were used and adapted for websites that only allowed limited search functions.

**Figure 2:**
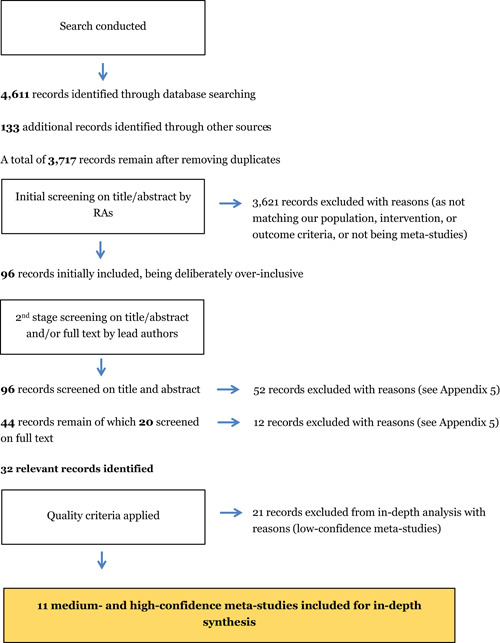
PRISMA flow diagram. [Color figure can be viewed at wileyonlinelibrary.com]

After removing duplicates, 3,717 records were screened independently by title and abstract by two RAs, with quality assurance from both lead review authors (MD and PM). On the basis of the title and abstract screening, 3,621 records were removed, leaving 96 records to be independently screened by the two lead review authors. Of these 96 records, 52 were excluded based on title and abstract screening. Twenty records required full text review, which led to exclusion of an additional 12 studies, that is, a total of 64 studies were excluded (see Appendix 5 for details) leaving a final sample of 32 studies for data extraction—Figure 2 below provides more details.

After the search and screening process, a quality appraisal was conducted (described in Section 6.2), which disaggregated the sample of 32 included studies by levels of confidence. Twenty‐one studies were classified as low‐confidence and 11 studies as medium‐ or high‐confidence (see Section 5.3.3, above, and Appendix 7). As outlined in the protocol (Duvendack & Mader, 2018), the in‐depth synthesis presented in this report focuses on the 11 medium‐ and high‐confidence studies only. As Appendix 9 shows with an overview of the results on the quality assessment criteria, 21 meta‐studies were classified as low‐confidence not on the grounds of a few specific criteria, but generally due to shortcomings on numerous criteria that contributed to their classification as low‐confidence. Often, unclear or only partial reporting (rather than outright failure to meet particular criteria) played a role.

#### 6.1.2 Included studies

##### 6.1.2.1 Key characteristics

Table 3 describes the key characteristics of the 32 included meta‐studies. The table is split into two parts: the top half presents the summary characteristics of the 11 medium‐ and high‐confidence studies, presents this information for the 21 low‐confidence studies. Of the 32 studies, 22 had global geographical coverage, while 5 focused on South Asia, 2 on Sub‐Saharan Africa and in the case of 3 studies the geographical focus was not explicitly mentioned but it can be assumed that the focus was global.

**Table 3:** Summary of included meta‐studies

Authors	Year	Source	Type of meta‐study	Geographic focus	Broad outcome categories	Confidence	Participants	Financial inclusion category					
								Micro‐credit	Micro‐insurance	Micro‐savings	CBSGs	Mixed micro‐finance	No. of primary studies
*11 medium‐ and high‐confidence meta‐studies (included for in‐depth synthesis)*:													
Steinert et al.	2018	Peer‐reviewed journal	Systematic review & Meta‐analysis	Sub‐Saharan Africa	Economic, social and behavioural	High	Household, individual, microenterprise			X			27
Vaessen et al.	2013	Final report	Systematic review & Meta‐analysis	Global	Gender	High	Individual	X					25
Brody et al.	2015	Final report	Systematic review & Meta‐analysis	Global	Gender	Med‐high	Individual, group				X		34
Stewart et al.	2012	Working paper	Systematic review	Global	Economic	Med	Household, individual	X		X		X	17
Duvendack et al.	2011	Technical report	Systematic review	Global	Economic, social, gender and mixed	Med	Household, individual, microenterprise	X				X	58
Orton et al.	2016	Peer‐reviewed journal	Systematic review	Global	Social, gender and behavioural	Med‐low	Household, individual	X			X	X	31
Gopalaswamy et al.	2016	Working paper	Systematic review & Meta‐analysis	South Asia	Economic, social and gender	Med‐low	Household, individual, microenterprise	X	X	X	X	X	69
Peters et al.	2016	Technical report	Systematic review	South Asia	Economic, social and gender	Med‐low	Household, individual, Communities	X	X	X	X		20
Stewart et al.	2010	Technical report	Systematic review	Sub‐Saharan Africa	Economic, social and gender	Med‐low	Household, individual, microenterprise	X		X			15
Chliova et al.	2015	Peer‐reviewed journal	Systematic review & Meta‐analysis	Global	Economic, social and gender	Med‐low	Household, individual, microenterprise	X					90
Kennedy et al.	2014	Peer‐reviewed journal	Systematic review	Global	Gender and behavioural	Med‐low	Individual	X			X	X	12
*21 low‐confidence meta‐studies (not included in‐depth synthesis)*													
Habib et al.	2016	Peer‐reviewed journal	Systematic review	Global	Economic, social and behavioural	Low	Household individual		X				23
Lorenzetti et al.	2017	Peer‐reviewed journal	Systematic review	Global	Social and behavioural	Low	Household individual	X	X		X	X	35
Cole et al.	2012	Technical report	Systematic review	Global	Behavioural	Low	Household individual		X				13
Maîtrot & Niño‐Zarazúa	2017	Working paper	Systematic review	Unclear	Economic	Low	Household individual microenterprise village					X	54
Pande et al.	2012	Working paper	Systematic review	Global	Economic	Low	Household individual microenterprise			X			12
Apostolakis et al.	2015	Peer‐reviewed journal	Systematic review	Global	Economic and social	Low	Household individual programme/institution		X				64
Arrivillaga & Salcedo	2014	Peer‐reviewed journal	Systematic review	Global	Social	Low	Household individual	X		X		X	14
Bhageerathy et al.	2017	Peer‐reviewed journal	Systematic review	South Asia	Behavioural	Low	Household individual				X		20
Awaworyi Churchill et al.	2016	Peer‐reviewed journal	Systematic review & Meta‐analysis	South Asia	Economic	Low	Household individual	X					8
Awaworyi Churchill	2015	Book chapter	Systematic review & Meta‐analysis	Global	Gender	Low	Individual	X		X		X	7
Madhani et al.	2015	Peer‐reviewed journal	Systematic review	South Asia	Gender and social	Low	Individual					X	12
Marr et al.	2016	Peer‐reviewed journal	Systematic review	Global	Behavioural	Low	Individual		X				45
O'Malley & Burke	2017	Peer‐reviewed journal	Systematic review	Unclear	Social, gender and behavioural	Low	Household individual	X				X	41
Awaworyi Churchill	2014	Working paper	Systematic review & Meta‐analysis	Global	Economic	Low	Household individual microenterprise	X					25
Gammage et al.	2017	Working paper	Systematic review	Global	Economic, social, gender and behavioural	Low	Household individual community group					X	594
Gash	2017	Learning brief	Systematic review	Global	Economic, social and gender	Low	Household individual				X		53
Hidalgo	2009	Master's thesis	Systematic review & Meta‐analysis	Global	Economic	Low	Household individual microenterprise	X					30
Isangula	2012	Peer‐reviewed journal	Systematic review	Unclear	Social, gender and behavioural	Low	Individual	X			X	X	49
O'Grady	2016	Coursework	Systematic review	Global	Economic	Low	Individual	X		X			38
Palmkvist & Lin	2015	Bachelor's Thesis	Systematic review	Global	Gender	Low	Individual				X		12
Yang & Stanley	2013	Working paper	Systematic review & Meta‐analysis	Global	Economic	Low	Household individual	X					13

*Notes*: We explain the confidence categories in more depth in the section “Assessment of methodological quality of included reviews”.

The meta‐studies covered a range of financial inclusion interventions (Appendix 3 provides details on the main research questions of each of the included reviews). We categorised studies by five broad intervention types: microcredit, microinsurance, microsavings, CBSGs and broader/mixed microfinance interventions. The latter category mainly refers to interventions that provided either a mixture of services or an unclear combination of services; it may also contain further sub‐intervention types, such as financial literacy or financial skills training, money transfers and other types of activities which the studies themselves may or may not specify. The table below indicates that out of 32 studies, the majority of studies (*n *= 19) focused on one intervention type—predominantly microcredit (six studies) or micro‐savings/CBSGs (also six studies)—followed by four studies covering two types, six studies covering three types and three studies being broader, covering four or five intervention types (two and one studies, respectively). In Appendix 4, we provide additional information describing the included studies, disaggregating them using PICOS^23^ criteria and level of confidence to get a better overview of which sub‐themes are covered by how many and by what proportion of reviews.

**Figure 3:**
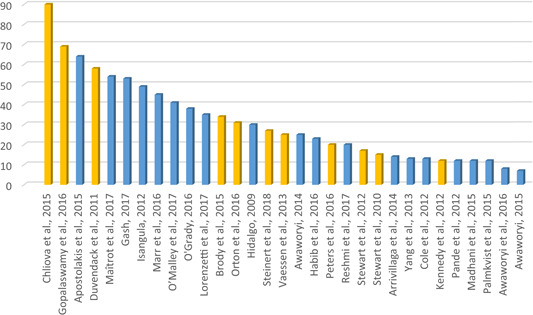
Number of primary studies per meta‐study (11 medium‐/high‐confidence meta‐studies highlighted). *Note*: Gammage et al. (2017) is excluded from this figure as it includes 594 primary studies. [Color figure can be viewed at wileyonlinelibrary.com]

The number of primary studies included in each of the 32 studies varied, ranging from 7 to 594 studies, as outlined in Figure 3 below. The mean value of included primary studies across the 32 studies is 49; however, this mean value is driven up by Gammage et al. (2017), who included 594 studies. Removing Gammage et al. (2017), we arrive at a mean value of 31 primary studies per review, with a range of 7–90 studies. This potentially raises the issue of small‐sample bias, which we will discuss in Section 7, further below.

Quantitative research designs dominate the primary evidence included in the 32 meta‐studies; that is, this is the case for 90% of our reviews. This quantitative focus, however, does not necessarily translate into quantitative synthesis methods. On the contrary, often due to high levels of heterogeneity within the primary evidence base, the preferred synthesis method across the 32 included meta‐studies is narrative in nature, with 53% of all included studies performing a narrative synthesis versus 22% that adopted a quantitative synthesis method, and 25% pursuing a mixed methods synthesis (Table 4 below). Mixed methods synthesis approaches play a greater role among the medium‐ and high‐confidence reviews.

**Table 4:** Synthesis methods used by meta‐studies of different confidence levels

Type of synthesis method	Number of meta‐studies	% of meta‐studies	21 low confidence meta‐studies		11 medium/high confidence Meta‐studies	
			Number	%	Number	%
Quantitative synthesis methods	7	22	5	24	2	18
Narrative synthesis methods	17	53	12	57	5	45
Mixed quantitative‐narrative synthesis methods	8	25	4	19	4	37
Total	32	100	21	100	11	100

##### 6.1.2.2 Outcomes

A wide range of intervention types across broad geographical focal areas are examined across the 32 included studies. The coverage in terms of outcomes is equally broad, as presented in Table 5.

**Table 5:** Summary of included studies by synthesis approach and number of outcomes reported

Study	No. of reported outcomes	How is the outcome reported?			Type of outcome—broad category				
		Meta‐analysis result	Mixed quant‐qual approach	Narrative synthesis	Behavioural	Economic	Gender	Social	Mixed
Total	183	63	24	96	22	72	38	37	14
*11 medium‐ and high‐confidence studies*	87	35	12	40	8	41	17	16	5
Steinert et al. (2018)	14	14	0	0	4	9	0	1	0
Vaessen et al. (+++2013)	1	1	0	0	0	0	1	0	0
Brody et al. (2015)	7	5	1	1	0	0	7	0	0
Stewart et al. (2012)	9	0	0	9	0	9	0	0	0
Duvendack et al. (2011)	11	0	11	0	0	2	2	2	5
Orton et al. (2016)	5	0	0	5	1	0	1	3	0
Gopalaswamy, Babu, and Dash (2016)	12	6	0	6	0	8	2	2	0
Peters et al. (2016)	3	0	0	3	0	1	1	1	0
Stewart et al. (2010)	12	0	0	12	0	6	1	5	0
Chliova, Brinckmann, and Rosenbusch (2015)	9	9	0	0	0	6	1	2	0
Kennedy, Fonner, O'Reilly, and Sweat (2014)	4	0	0	4	3	0	1	0	0
*21 Low‐confidence studies*	96	28	12	56	16	33	25	22	0
Habib, Perveen, and Khuwaja (2016)	6	0	0	6	3	1	0	2	0
Lorenzetti, Leatherman, and Flax (2017)	4	0	0	4	1	0	0	3	0
Cole et al. (2012)	2	0	2	0	2	0	0	0	0
Maitrot and Niño‐Zarazúa (2017)	2	0	0	2	0	2	0	0	0
Pande et al. (2012)	5	0	5	0	0	5	0	0	0
Apostolakis, van Dijk, and Drakos (2015)	2	0	0	2	0	1	0	1	0
Arrivillaga and Salcedo (2014)	2	0	0	2	0	0	0	2	0
Bhageerathy, Nair, and Bhaskaran (2017)	2	0	0	2	2	0	0	0	0
Awaworyi Churchill, Danso, and Appau (2016)	4	4	0	0	0	4	0	0	0
Awaworyi Churchill, Korankye Danso, and Appau (2015)	10	10	0	0	0	0	10	0	0
Madhani, Tompkins, Jack, and Fisher (2015)	3	0	3	0	0	0	2	1	0
Marr, Winkel, van Asseldonk, Lensink, and Bulte (2016)	2	0	0	2	2	0	0	0	0
O'Malley and Burke (2017)	5	0	0	5	3	0	1	1	0
Awaworyi Churchill (2014)	13	13	0	0	0	13	0	0	0
Gammage et al. (2017)	7	0	0	7	2	2	2	1	0
Gash (2017)	12	0	0	12	0	2	1	9	0
Hidalgo (2009)	1	0	1	0	0	1	0	0	0
Isangula (2012)	8	0	0	8	1	0	5	2	0
O'Grady (2016)	1	0	1	0	0	1	0	0	0
Palmkvist and Lin (2015)	4	0	0	4	0	0	4	0	0
Yang and Stanley (2013)	1	1	0	0	0	1	0	0	0

To grapple with the sheer number of outcomes identified across the 32 included studies, we categorised them by five broad outcome categories, with further sub‐categories: Behavioural, economic, gender, social and, finally, mixed outcomes when they could not be clearly slotted into any of the other four outcome categories (in the synthesis of results, this mixed category was not used, and rather these outcomes were integrated into the other four categories). A total of 183 outcomes are reported with 87 outcomes reported across the 11 medium‐ and high‐confidence studies and 96 outcomes reported across the 21 low confidence studies. All studies report on multiple (sub‐) outcomes across all broad outcome categories.

**Figure 4:**
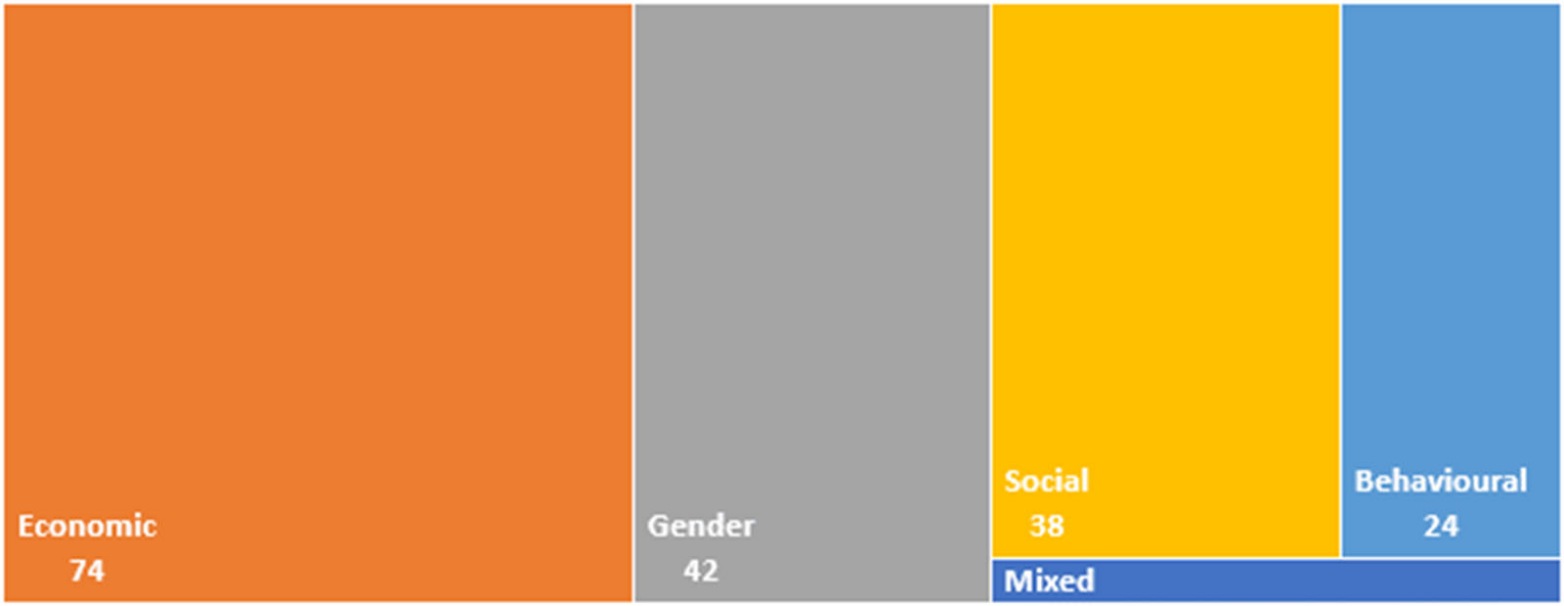
Number of outcomes by broad category. [Color figure can be viewed at wileyonlinelibrary.com]

Looking across the 32 included studies, the number of outcomes by broad outcome category are as follows (see Figure 4), a clear focus on economic outcomes (reported 74 times), followed by gender (reported 42 times), social (reported 38 times) and behavioural outcomes (reported 24 times) can be observed.

It is worth breaking down these broad outcome categories into further sub‐categories to better understand the high levels of heterogeneity within each broader category across all the 32 reviews we included.

**Figure 5:**
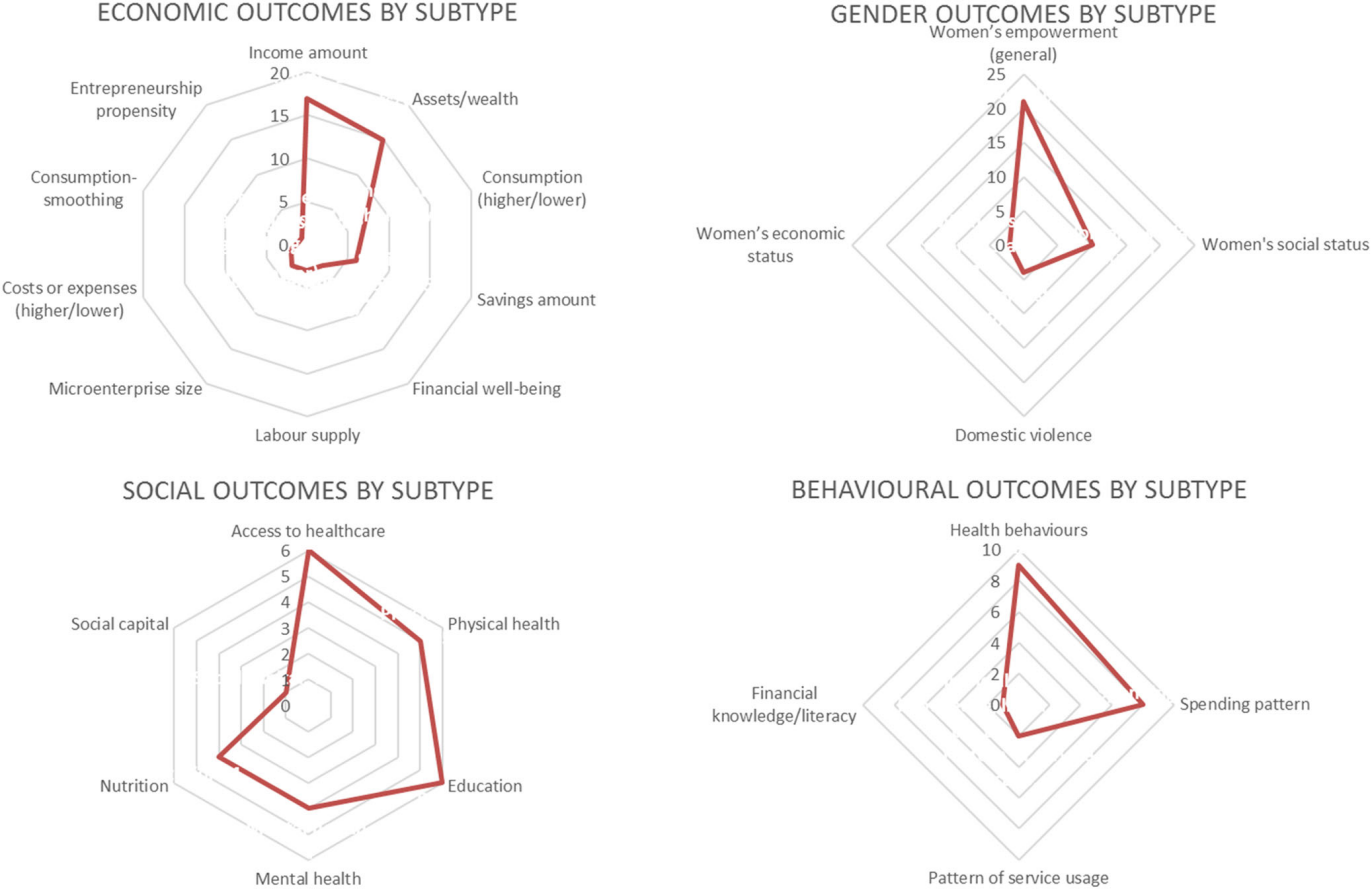
Broad outcome category by outcome sub‐category/type. [Color figure can be viewed at wileyonlinelibrary.com]

From the collation of charts under Figure 5, we can see a wide range of sub‐categories within each of the broader outcome categories. For instance, within economic outcomes, income (reported 17 times) and assets/wealth (reported 15 times) clearly dominate, followed by consumption (reported seven times), and savings amount (reported six times) and financial well‐being, labour supply, size of microenterprise, reported only three times. Within the broader gender category, women's empowerment is mostly reported in general terms (21 times), followed by women's social status (10 times). The picture is more mixed when looking at what we categorised as “social” outcomes, where access to healthcare and education are reported six times each, followed by physical health (reported five times), mental health and nutrition (reported four times each). Behavioural outcomes appear equally mixed, with health behaviours (largely due to two health‐oriented meta‐studies) and spending patterns/behaviour dominating, reported nine and eight times, respectively. Overall, high levels of heterogeneity of outcome categories and subcategories can be found across the 32 included systematic reviews. It is also worth noting that many of the outcomes we identified seem to be located in the early stages of the causal chain; we discuss this in more depth further below.

**Figure 6:**
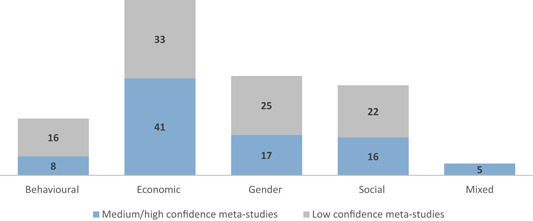
Number of outcomes by outcome type. [Color figure can be viewed at wileyonlinelibrary.com]

To examine how this information may differ by quality of studies, we have distilled elements of Table 4 into additional graphics to highlight key insights. Figure 6 below presents the number of outcomes reported, comparing medium‐ and high‐confidence studies with low confidence studies. In line with the findings above, economic outcomes still dominate across all studies, irrespective of quality, followed by gender and social outcomes.

**Figure 7:**
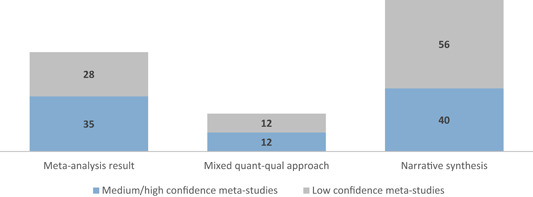
Number of outcomes by synthesis approach. [Color figure can be viewed at wileyonlinelibrary.com]

Worth examining also is the number of outcomes reported by type of synthesis approach, as outlined in Figure 7, below. Irrespective of confidence level, the majority of outcomes are reported adopting a narrative synthesis approach, with only a third of outcomes reported using meta‐analysis. Having said that, there is a slight tendency for the higher‐confidence meta‐studies to report meta‐analytical findings, which can be explained by the higher number of meta‐analyses and mixed approaches classified as medium or high confidence meta‐studies.

##### 6.1.2.3 Gender

**Figure 8:**
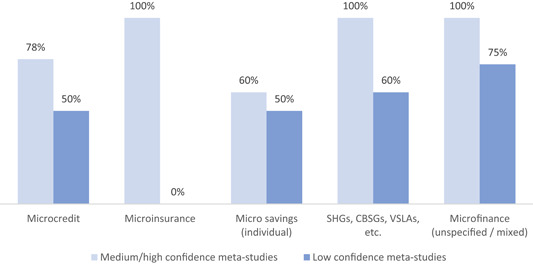
Proportion of meta‐studies focusing on women, by financial inclusion category. [Color figure can be viewed at wileyonlinelibrary.com]

Given the prominence of gender outcomes in our 32 included meta‐studies, and the specific targeting of women by many financial inclusion interventions, we should note that in our sample of medium‐ and high‐confidence studies 73% focus on gender issues while only 43% of the low confidence studies have a gender focus. Figure 8 provides further disaggregated information on the proportion of meta‐studies focusing on women, by type of financial inclusion intervention. We have adopted the five broad categories we had outlined above and find that within our sample of medium‐ and high‐confidence studies, 100% of the general microfinance, micro‐insurance and community‐savings interventions focus on women. Seventy‐eight percent of reviews of micro‐credit interventions focus on women, while it is 60% for micro‐savings schemes. The picture is more mixed among the sample of low confidence studies, where only 50% of the reviews of micro‐credit and micro‐savings focus on women.

#### 6.1.3 Excluded studies

During title and abstract as well as full‐text screening by the two lead authors, 64 studies (details in Appendix 5) were excluded, largely because of not meeting the inclusion criteria for “types of intervention”—this applies to 27 studies, which did not address at least one or more type of intervention that clearly aimed at financial inclusion. Eleven studies did not meet the inclusion criteria for “types of reviews” or “study design”; notably, some studies were labelled by their authors as systematic reviews, but did not meet basic criteria for any systematic review or the definition we put forward in the “Methods” section. One study did not meet the inclusion criteria for “types of participants”, or “population”, and 25 additional duplicates were identified.

#### 6.1.4 Independence of reviews—Overlap

**Figure 9:**
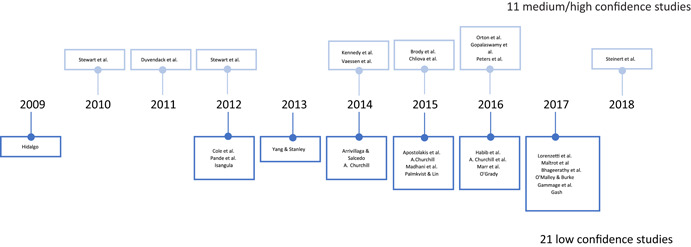
Publication years for all 32 meta‐studies. [Color figure can be viewed at wileyonlinelibrary.com]

Given that all our included meta‐studies were published within less than a decade of one another (see Figure 9, below), the question of overlap arises. In other words, it is highly likely that many of our included reviews have common interests and overlap in terms of the main review questions they pose (see Appendix 3 for an overview of review questions of all included studies). Overlap can also occur in all or some aspects of the PICOS criteria (see Appendix 4 for a breakdown of the proportion of reviews covering common PICOS criteria).

Our strategy to deal with overlap was to closely examine the main review questions of each of the reviews and to investigate whether our 32 included studies would draw on the same core pool of primary studies for synthesis and policy recommendations. In case of considerable overlap, we were prepared to remove the review(s) in question in order to avoid duplication.

Examining the review questions for overlap was less straightforward, as each review had very distinct focal areas (as presented in Section 6.1, where we outlined the high levels of heterogeneity we found across all included studies, especially in terms of geographical focus but also in terms of types of interventions and outcomes). To investigate whether the same pool of primary studies had formed the basis of many of our reviews, we tasked two RAs to list all primary studies included in the 32 meta‐studies and count how many times each of the included primary studies was included in the synthesis of each of the reviews. Contrary to our expectations, we found only limited overlap across all 32 meta‐studies. Overlap within the pool of low‐confidence studies was more substantial, compared to the medium‐ and high‐confidence studies.

In the case of the 11 medium‐ and high‐confidence studies (see Table 6), we find that the highest correlations occur between Chliova et al. (2015) and many of the earlier meta‐studies, notably Stewart et al. (2010, 2012—a correlation of 47% for the latter). Duvendack et al. (2011) and Vaessen et al. (2014), which may be due to the broad scope of the Chliova et al. (2015) review. There is also considerable overlap between Duvendack et al. (2011) and Stewart et al. (2012)—41%—, which is not surprising as both studies were published only a year apart and Stewart et al. (2012) clearly stated in their review that they built on and expanded (i.e., included micro‐insurance and micro‐leasings) on the evidence base synthesised by Duvendack et al. (2011). There is also notable overlap between Duvendack et al. (2011) and Gopalaswamy et al. (2016), and it is less clear why this is the case. One explanation could be the focus on South Asia, because Duvendack et al. (2011) pool of included primary studies was dominated by evidence from South Asia while Gopalaswamy et al. (2016) had a sole focus on South Asia.

**Table 6:** Correlation matrix of medium/high confidence meta‐studies to demonstrate overlap

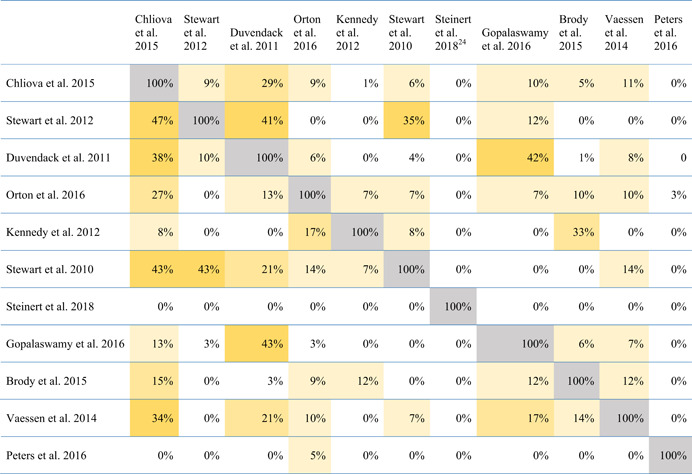

^a^ There is indeed no overlap of Steinert et al. (2018) with any other review, this is largely due to the narrow focus of Steinert et al. (2018), that is, only RCTs capturing microsavings interventions in the African context are included, but also due to the publication dates of many of the included studies (the majority of the included studies were published in 2016 and 2017).

However, none of the correlations in the pool of medium‐ and high‐confidence studies exceeded 50%, which is different for the 21 low confidence studies (see Appendix 6 for details), where we found correlations of up to 83% (between Awaworyi et al. 2014, 2016) and 71% (between Awaworyi et al. 2014; Awaworyi Churchill et al., 2015). The correlation between Awaworyi et al. (2016) and Maitrot and Niño‐Zarazúa (2017) is 67% and correlations between Maitrot and Niño‐Zarazúa (2017), Awaworyi et al. (2014) and Yang and Stanley (2013) are 83%. Lorenzetti et al. (2017) and Arrivillaga and Salcedo (2014) are correlated by 43%. We would have certainly excluded at least one of the Awaworyi studies on the basis of these findings, but given they are both low‐confidence, this was not necessary. In Section 6.3, we summarise only the results of the medium‐ and high‐confidence meta‐studies.

**Figure 10:**
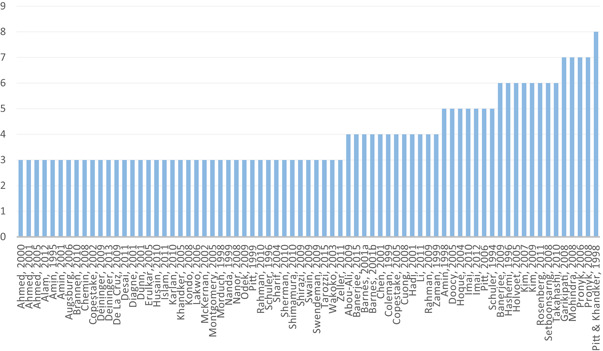
Number of included primary studies, three times or more. [Color figure can be viewed at wileyonlinelibrary.com]

A final point on overlap: we were curious to find out whether a particular set of studies would stand out and dominate some, or any, of our 32 meta‐studies. The seminal paper by Pitt and Khandker (1998), we found, was included eight times, which is particularly interesting, because this study has been criticised extensively due to challenges in replicating its original findings that cast doubts on its reliability (for an overview, see e.g., Duvendack et al., 2011; Stewart et al., 2012). We discuss this issue in more depth in Section 6.2.2. Studies by Mohindra, Haddad, & Narayana, 2008; Pronyk et al. (2006 and 2008) and Garikipati (2008) were included seven times, followed by Banerjee, Duflo, Glennerster, and Kinnan (2009), Hashemi, Schuler, and Riley (1996), Holvoet (2005); Kim et al. (2007 and 2009), Rosenberg, Seavey, Jules, and Kershaw (2011), Setboonsarng and Parpiev (2008) and Takahashi, Higashikata, and Tsukada (2010) which were all included six times. Figure 10 below lists all studies that were included three times or more in any of the 32 included meta‐studies.

### 6.2 Risk of bias in included studies

#### 6.2.1 Assessment of methodological quality of included reviews

The quality appraisal or risk‐of‐bias process in the context of reviewing systematic reviews is different from the standard process commonly used in systematic reviews themselves. This is due to the unique methodological characteristics of systematic reviews, which need to be reflected in the quality appraisal criteria. As outlined in Section 5.3.3. and Table 2, above, two different tools exist to assess the quality of systematic reviews, without much consensus in the literature on which is to be preferred. Hence, we used both tools—AMSTAR 2(24) and an adapted version of the 3ie critical appraisal checklist^25^—to assess the quality (or, as we interpret the outcomes of this appraisal process: our *confidence* in their management of the risk of bias) of the 32 meta‐studies that we included, as discussed in depth in the “Data collection and analysis” section above.

AMSTAR 2 is based on 16 criteria, each allowing a rating of “yes”, “partial yes”, or “no”, adding up to a summary assessment of the quality of the systematic review. Shea et al. (2017) note, however, that there is an element of subjectivity in using the AMSTAR 2 tool, which requires users to exercise their own judgement in making final decisions on the quality of systematic reviews. AMSTAR emphasises formal elements of methods and analysis over other potentially important aspects such as content, thematic importance, or wider contribution to the literature, and thus could lead to exclusion of nonetheless important or useful reviews. The element of subjectivity is part of the reason for using another tool to corroborate and complement the findings of AMSTAR 2. There is a degree of overlap between AMSTAR 2 and the 3ie critical appraisal checklist (see Appendix 7 for details on both tools), but also some differences, especially in relation to assessing whether reviews analysed the intervention's causal chain.

Given the importance of unpacking causal mechanisms to understand how, why and for whom an intervention works, we adapted the 3ie checklist to include criteria that relate to the explicit use of theory in meta‐studies and to what extend an analysis of the causal chain is undertaken (see Appendix 7, Table A, 7.2, section D1‐D7). We find that none of the 32 included meta‐studies scored very highly in this regard, suggesting that more encouragement would be needed to ensure reviewers explicitly incorporate theory in the systematic review process.

It is worth mentioning that AMSTAR 2 in particular very much focuses on technical and procedural aspects of the systematic review process, such as: did the meta‐study include all components of PICO? Did the review authors perform study selection and data extraction in duplicate? Did authors use a satisfactory technique to assess risk of bias? Were appropriate statistical techniques used if meta‐analysis was conducted? In the case of some of the reviews we examined, answers to these questions were not reported in the final published study, which may partially explain their low quality assessment; that is, why we have low confidence in their results. Lack of reporting of substantive issues related to processes, methods and data is a frequent occurrence, not just in the context of meta‐studies but of primary studies, too. In other words, if a particular meta‐study is categorised as low quality, or low confidence, according to AMSTAR 2 or the 3ie tool, this does not necessarily mean that it does not substantially contribute to the discussion of financial inclusion impacts. But it does mean that the evidence for it meeting certain “critical domains” (Shea et al., 2017, p. 5) that affect the validity of reviews and its conclusions was too limited for us to treat it with high confidence.

Table 7, above, presents the 11 included studies that were classified as high‐ and medium‐confidence, while the remaining 21 included studies achieved a low confidence rating (see Appendixes 8 and 9). We class as medium‐low or medium‐confidence those that were classed as at least “moderate confidence” using the AMSTAR 2 tool or “medium confidence” using the adapted 3ie checklist (eight studies). We class as medium‐high or high‐confidence or those meta‐studies that were classed as “high confidence” by at least one of the tools (three studies). The synthesis presented in the next section will highlight and discuss the findings of the 11 high‐ and medium‐confidence systematic reviews in more depth.

**Table 7:** Quality assessment of meta‐studies included for in‐depth review

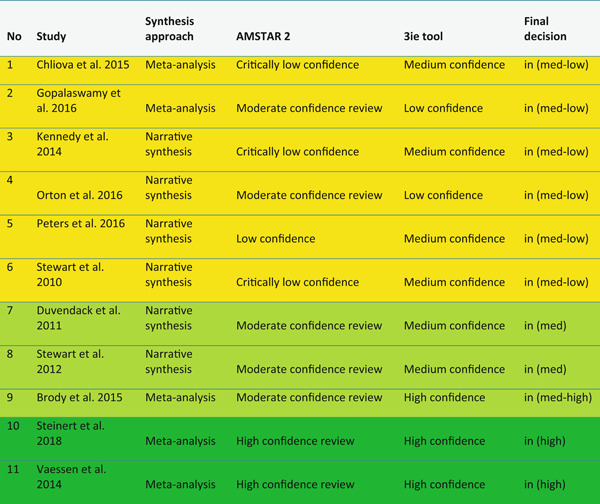

As a final note on quality of the meta‐studies, we should point out some discrepancies in the results of the ratings of the AMSTAR 2 and 3ie tools. For instance, the study by Chliova et al. (2015) achieved a critically low rating in AMSTAR 2, but a medium‐confidence rating on the 3ie checklist. Where this type of discrepancy was the case, we would accept the decision of the tool with the higher, more positive, rating, and assign it—as in the case of the Chliova et al. (2015) study—a final label of “medium‐low” confidence. Interestingly, the ratings of both tools differ mostly for the studies on the lower‐confidence spectrum, leading to six final medium‐low ratings. We take the fact that there were never any complete discrepancies between the two quality rating tools—such as “high confidence” on AMSTAR 2 and “low confidence” on 3ie—as an encouraging sign that the tools reasonably consistent with one another, and may be amenable to further harmonisation in future. On the issue of discrepancies, we should also note that we had some disagreements with our RAs on the results of their quality appraisals across both tools, which were resolved using our consensus procedures. However, these disagreements may suggest that applying these tools requires expert knowledge and that there is an element of subjectivity in assessing the quality of reviews, as discussed by Shea et al. (2017). We would not be surprised if a different review team would reach slightly different conclusions in terms of critical quality appraisal for some of the meta‐studies. In fact, we categorised some meta‐studies as low confidence, following the application of the criteria, even though we felt they merited inclusion and would have enriched our review. Future systematic reviews of reviews may want to carefully consider whether a rigid quality appraisal process is the right way forward.

#### 6.2.2 Assessment of methodological quality of primary studies included in reviews

The quality assessment of the included studies using AMSTAR 2 and the 3ie critical appraisal checklist led to the removal from in‐depth review of a large number of studies: 21 to be precise. We will not discuss their findings or evidence base in any further depth. As a next step, we must examine the quality of the primary evidence that underlay the remaining 11 medium‐ and high confidence‐reviews. Sixty‐four percent of the medium‐ and high‐confidence reviews indicate limitations that led to the inclusion of at least some low‐quality primary evidence. For example, Brody et al. (2015) note that

“both the quantitative and the qualitative primary studies suﬀered from limitations related to their quality” (p. 36).

Similarly, Vaessen et al. (2014) write that

“in terms of methodological quality, apart from Husain, Mukherjee, and Dutta (2010), all studies suffered from threats to validity” (p. 185).

Gopalaswamy et al. (2016) acknowledge that

“the studies with low risk of bias have low overall effect sizes compared to studies with medium and high risk of bias across outcome indicators. This indicates that there exists the possibility of exaggerated effects, arising from low‐quality impact evaluation” (p. 7).

Duvendack et al. (2011) go so far as to say that

“almost all impact evaluations of microfinance suffer from weak methodologies and inadequate data [….] This can lead to misconceptions about the actual effects of a microfinance programme, thereby diverting attention from the search for perhaps more pro‐poor interventions” (p. 4).

Finally, Steinert et al. (2018) conclude that

“unreliable or biased results may lead to erroneous conclusions” (p. 242).

In short, there are major concerns in relation to the quality of the primary evidence that informed the findings of even the medium‐ and high‐confidence meta‐studies we include in our review. Hence, it pays to be cautious about how much one can trust the overall findings presented in the systematic review evidence, as one simply cannot transform “dross into gold” (Morgenson and Rosner, 2011, p. 280), by combining a wide range of low quality studies into systematic reviews to enhance their profile; this would be analogous to what financial institutions did during the 2008 financial crisis when they pooled dubious cash‐flow generating assets such as low‐quality mortgages, bonds and loans into collateralised debt obligations (CDOs), to effectively convert risky assets into triple A‐rated products. Having said that, several of the meta‐studies we include sought to mitigate their quality concerns by providing sub‐group analysis, disaggregating their findings by risk of bias profile. For instance, Brody et al. (2015) and Vaessen et al. (2014) would distinguish between findings from studies with high, medium and low risk of bias. In the case of Brody et al. (2015), high risk of bias studies would present biased estimates exaggerating the impact of SHGs on women's empowerment, while the low and medium risk of bias studies were less biased, still presenting positive impacts, but less strong (p. 36). This is in line with what Duvendack et al. (2011) and Gopalaswamy et al. (2016) find, too (see quotes above).

To conclude this section, we should note that a wide range of quality assessment tools were used in the 11 included studies, such as a customised risk of bias tool adapted from the Cochrane Handbook (see Higgins and Green, 2011) and EPPI‐Centre guidelines (e.g., Gough, 2007) used by Duvendack et al. (2011), Vaessen et al. (2014) and Gopalaswamy et al. (2016). EPPI‐Centre methods also guided the quality appraisal tools used by Stewart et al. (2010 and 2012). Brody et al. (2015) adapted a 3ie quality tool for their purposes and Steinert et al. (2018) used the Cochrane Risk of Bias Assessment Tool. Orton et al. (2016) used a tool that captures six domains: selection bias, study design, confounders, blinding, data collection, and withdrawals and dropouts, which are then combined to produce an overall quality rating. Kennedy et al. (2014) adopt a different set of criteria related to (a) prospective cohort; (b) control/comparison group; (c) pre‐/post‐intervention data; (d) random assignment to intervention; (e) random selection for assessment; (f) follow‐up ≥80%; (g) socio‐demographic equivalence; and (h) baseline outcome measure equivalence. Chliova et al. (2015) did not assess the quality of their included studies. Peters et al. (2016) is the only study using the JBI QARI Critical Appraisal Checklist for Interpretive and Critical Research, as they only included qualitative evidence.

The uncertainty about the quality of the underlying evidence base—and the debatability of many paper's methods—is worth keeping keep in mind when assessing the overall reliability of the findings presented by the 11 medium‐ and high‐confidence reviews in the next section.

### 6.3 Synthesis of results

#### 6.3.1 Overview

Our approach to synthesising the results is a theory‐based mixed methods synthesis, with a focus on narrative synthesis that incorporates qualitative and quantitative elements, as appropriate. As mentioned above, our synthesis focuses on the 11 medium‐ and high‐confidence studies, of which eight synthesised only quantitative research, two synthesised both qualitative and quantitative data (Brody et al,. 2017; Stewart et al., 2010) and one synthesised purely qualitative data (Peters et al., 2016). Table 8 below contains the summary headline findings for each of these 11 meta‐studies.

**Table 8:** Summary of results from 11 medium‐ and high‐confidence meta‐studies

Study	Focus	Short description of findings	Meta‐analysis?
Brody et al. (2015) (medium‐high confidence)	Effect of SHGs on women's empowerment in South Asia	Women's self‐help groups have a positive effect on women's political empowerment, women's mobility, and women's control over family planning, but there is no rigorous evidence for SHGs reducing domestic violence or having positive effects on psychological empowerment (self‐confidence and self‐esteem)	Yes
Chliova et al. (2015) (medium‐low confidence)	Effect of microcredit on business ventures	Microcredit has significant positive effects on venture size and proﬁtability, but not on the survival of ventures. There are large positive effects on women's empowerment and small beneﬁcial effects on health and nutritional outcomes and on educational outcomes for clients’ children, but these are potentially offset by negative factors. Effects of microcredit are heterogeneous and context‐dependent	Yes
Duvendack et al. (2011) (medium confidence)	Effects of microfinance (mainly microcredit) on economic, social and empowerment outcomes	Studies on microfinance report many positive effects, but offer no convincing evidence of impacts on overall well‐being, due to the evidence base being too weak to draw robust conclusions. There is no clear evidence for positive economic outcomes or empowerment, and some indications of negative effects. Most impacts (positive or negative) are early in the causal chain	No
Gopalaswamy et al. (2016) (medium‐low confidence)	Effects of various microfinance types (incl. SHGs) on economic and social well‐being in South Asia	Microfinance has positive but small effects on income, women's empowerment, employment, asset creation, and consumption expenditure. The poorest of the poor are more likely to experience larger positive effects on household consumption. The effects on education are mixed, as only some small effects are found for girls’ education	Yes
Kennedy et al. (2014) (medium‐low confidence)	Effects of microfinance (mainly credit plus health training/messaging) on HIV prevention	Microfinance alone had no effect on HIV prevention, and had mixed outcomes when combined with health education. No evidence was found for effects on HIV prevalence	No
Orton et al. (2016) (medium‐low confidence)	Health effects of group‐based microfinance (mainly credit)	The overall findings were inconclusive for empowerment and health outcomes. Membership in larger, well‐established schemes was associated with improvements in some health outcomes, especially maternal and child health, and use of contraceptives	No
Peters et al. (2016) (medium‐low confidence)	Participants’ views of microfinance (microcredit, microsavings, micro‐leasing, and micro‐insurance) in South Asia	Participants reported a variety of positive and negative experiences, which were heterogeneous and different for women and men. Microsavings and microcredit each had positive sides and downsides. Positive experiences included effects on clients’ health, children's health, asset‐building and empowerment; negative ones included debt‐induced stress and disempowerment. There were no conclusive findings on impact	No
Steinert et al. (2018) (high confidence)	Effects of savings promotion on savings, consumption and investment in Sub‐Saharan Africa	Savings promotion has relatively small but signiﬁcant positive effects on intermediate outcomes (savings amount and enterprise propensity) and on wider poverty measures (household expenditure, income, and food security). There are no effects on health or housing, and programmes’ effectiveness is lower for women. Programmes for improving access to savings services are effective, while demand‐enhancement (financial education) is not	Yes
Stewart et al. (2010) (medium confidence)	Effects of mixed microfinance (microcredit and microsavings) on incomes, wealth and non‐financial outcomes in Sub‐Saharan Africa	Microcredit has mixed impacts, and microsavings has no impact on income, but both have a generally positive impact on health, food security and nutrition. Evidence on education and women's empowerment remains unclear. There is some evidence that microcredit makes some people poorer	No
Stewart et al. (2012) (medium‐low confidence)	Effects of microfinance (microcredit, microsavings and micro‐leasing) on economic opportunities	Microsavings has no significant effect on engagement in economic opportunities, and there is only relatively weak and inconsistent evidence that microcredit has a positive influence on incomes. Microcredit may reduce savings, and has potential to inflict financial harm. Microcredit and microsavings do not impact on income diversification. There is no evidence for effects of micro‐leasing	No
Vaessen et al. (2014) (high confidence)	Effects of microcredit on women's control over household spending	There is no reliable evidence for impacts of microcredit on women's control over household resources, making it unlikely that microcredit has a substantial impact on women's empowerment in a broader sense	Yes

In the remainder of this section, we cluster the findings of studies by four outcome categories^26^: economic, social, behavioural and gender outcomes, as set out in the theory of change (see Figure 1) in the section on “How the intervention might work”, and where feasible, in presenting impact findings, differentiate between lower‐order and higher‐order outcomes along the causal chain.

A simplistic reading of the results summaries in Table 8 would suggest an overall positive, if mixed, set of findings. Nearly half (5 of 11) included meta‐studies come to generally positive conclusions about the relationship between financial services access and positive changes for poor people (Brody et al., 2015; Chliova et al., 2015; Gopalaswamy et al., 2016; Orton et al., 2016; Steinert et al., 2018). The other six come to mixed, neutral, or no conclusions about impact, and none conclude that the evidence suggests an overall negative effect of financial inclusion interventions. At the same time, the information in Table 8 points to very high levels of heterogeneity between the results of different interventions in terms of different outcomes for different people and in different contexts. There are heterogeneous and inconsistent findings reported within the meta‐studies (e.g., Steinert et al., 2018; Stewart et al., 2010) as well as across different meta‐studies (for instance Chliova et al., 2015 and Vaessen et al., 2014 reach divergent conclusions in terms of women's empowerment). No results are found for macro‐structural outcomes.

The bulk of reported findings in the literature regarding impacts are positive, with few negative ones. But the positive evidence should be taken with caution, due to the often low quality of the underlying primary evidence that informs the findings (see discussion in Section “Assessment of methodological quality of evidence in reviews”). Furthermore, and more notably, the effect sizes overall are quite small and do not plausibly indicate transformative changes.

Many of the effects we found are strongly heterogeneous, both across studies and over time, places, populations, and between interventions. There may also be an issue with small sample bias. Slavin and Smith (2009) and others (e.g., Kjaergard, Villumsen, & Gluud, 2001) suggest that reviews with small sample sizes (*n* < 100) tend to report larger, more positive effect sizes than reviews with larger sample sizes (*n* > 100), and that they are often of lower methodological quality. In the case of our medium‐ and high‐confidence reviews, the sample of primary studies they included range from 12 to 90, positioning our reviews in the small‐sample category. Thirty‐six percent of the 11 medium‐ and high‐confidence studies also voice concerns about the limited quantity of evidence they included.

We should also note that positive findings tend not to repeat from one context to another. At least as many findings are mixed or inconclusive as are positive. With reference to the financial inclusion theory of change (presented in the “Background” section), most of the positive impact estimates are for outcomes that are early along the causal chain (discussed in more depth below), such as health‐focused meta‐studies finding changes in health knowledge, but not in health outcomes, or meta‐studies looking at enterprise activity finding growth in business ventures run by households but not in household incomes as a result. An exception appears to be for savings, where both immediate outcomes *and* wider poverty measures are affected in a positive but relatively small way.

In the following, more detailed, discussion of results, we cluster the findings of studies for four outcome categories—economic, social, behavioural and gender outcomes—and relate the findings to different financial intervention‐types (as applicable) with reference to the theory of change presented in the section “How the intervention might work”.

#### 6.3.2 Economic outcomes

As explained in the theory of change, in theory, financial inclusion could lead to benefits for poor people through changes in their financial behaviours such that they use financial services to gain access to new income sources or enhance existing ones, to save money that they would otherwise spend or lose, to invest in assets, to sustainably consume more goods, or to cope with shocks. Lower‐order outcomes, that is, outcomes found on outcomes early in the causal chain, would include the fact of having an enterprise, increasing the size of one's enterprise, accessing employment, saving more, and having smoother consumption patterns; these could, in turn, be enablers of higher‐order outcomes further along the causal chain. Higher‐order outcomes would include sustainably higher incomes and more assets or wealth. Consumption is an ambiguous indicator, because higher consumption might stem from a higher income and ability to consume or from higher costs that represent a financial drain; likewise, a reduction in consumption could indicate lower costs and more savings or financial distress or unsustainable consumption that has led to distress.

#### 6.3.3 Lower‐order/intermediate outcomes

##### 6.3.3.1 Entrepreneurship & microenterprise size

There were four meta‐studies that looked at microenterprise investment, size or profits as an outcome. Chliova et al. (2015) found significant results, indicating that microcredit leads to venture growth and increased proﬁts, but only a marginally significant effect on venture survival; they suggest that the small magnitudes do not indicate the effects of microcredit on microenterprise to be transformational. Steinert et al. (2018) found a small but significant effect from savings promotion interventions in Sub‐Saharan Africa on investment in family businesses, and similarly small but signiﬁcant downstream impacts on business returns and proﬁts. Stewart et al. (2010) found mixed (positive and negative) evidence on whether microcredit and microsavings lead to greater investment or growth in business assets in Sub‐Saharan Africa. Stewart et al. (2012) found only little reliable evidence (from one country) on microcredit increasing poor people's expenditures on business, and found that microsavings did not significantly increase poor people's engagement in economic opportunities.

##### 6.3.3.2 Labour supply

Two meta‐studies looked at labour supply and employment. Gopalaswamy et al. (2016) found there to be a marginal and insignificant effect on employment from mixed types of microfinance on employment in South Asia, with some underlying studies suggesting a small amount of employment generation and an increase in male hours of employment, but not female hours. The effects were more pronounced for broader microfinance approaches than for microcredit alone. Stewart et al. (2012) found little evidence t0 suggest that microcredit had any impact on job creation.

##### 6.3.3.3 Savings

Four meta‐studies engaged with savings as an outcome, one of them in depth. Steinert et al. (2018) meta‐analysis of savings programmes in Sub‐Saharan Africa found that programmes that focused on offering opportunities to save showed positive and signiﬁcant, though also relatively small, effect sizes in terms of savings amount. Programmes that focused on building savings by changing attitudes or overcoming behavioural constraints, by contrast, had no effect. The changes in savings amounts were greater for men than women, although this may have been due to the characteristics of the men and women involved. Stewart et al. (2010) found both microcredit and microsavings in Sub‐Saharan Africa to have positive impacts on poor people's savings levels, while also increasing their expenditure and asset accumulation. Stewart et al. (2012) found further evidence that access to microsavings facilities significantly increased people's savings amounts, though in some countries only for women. For microcredit, however, their most reliable evidence suggested it reduced savings, and their less reliable evidence suggested it increased savings or had no effect. Peters et al.’s (2016) qualitative review of user experiences in South Asia found that participants emphasised the importance of being able to save money, and savings had both functional and symbolic value for women.

##### 6.3.3.4 Costs or expenses

No meta‐study explicitly examined the effects of financial services on households’ costs or expenses. However, Peters et al.’s (2016) qualitative review noted that participants in South Asia appeared to appreciate microfinance as an offer to reduce their reliance on more expensive sources of credit. More broadly, no meta‐study reviewed evidence about the costs of financial inclusion borne by clients, particularly interest and fees paid to financial service providers.

##### 6.3.3.5 Consumption‐smoothing

The evidence for consumption‐smoothing and dealing with shocks (in the four meta‐studies that examine this) tends to be thin; this may at least partly stem from the methodological challenges of assessing it. Peters et al.’s (2016) review of qualitative evidence found participants in South Asia appreciated the ability to stabilise their families’ consumption patterns as one short‐term benefit of financial services. Gopalaswamy et al. (2016) found in South Asia that participation in microfinance programmes of various types dampened seasonal variations in agricultural incomes, albeit driven more by income‐smoothing effects (extra sources of income) than by borrowing and lending. Stewart et al. (2010) found some evidence that mixed microfinance (credit and savings) in Sub‐Saharan Africa enables poor people to deal better with shocks, but that this is not universal, and some evidence pointing to the contrary (some clients take their children out of school in response to shocks). Stewart et al. (2012) suggest that the ability to withstand shocks or increase wealth thanks to microcredit might be outweighed by the risks of increased debt and loss of collateral.

#### 6.3.4 Higher‐order/final outcomes

##### 6.3.4.1 Income

Moving on to higher‐level outcomes (which would indicate actual poverty reduction): four meta‐studies specifically examined the effects of financial services on incomes. The meta‐analysis by Gopalaswamy et al. (2016) found a small positive but statistically insignificant effect on income from mixed forms of microfinance in South Asia, while suggesting that broader microfinance interventions had a greater (but still insignificant) effect than simple microcredit initiatives. Their narrative synthesis also indicated a positive trend, albeit with variance across studies. Stewart et al. (2010, p. 48) similarly found for Sub‐Saharan Africa that microcredit had mixed impacts on income and microsavings had none. However, they also noted indications of a “worrying trend” that “the benefits of micro‐credit appear to diminish—and even become negative—the longer clients are enroled in a programme”, indicating harmful debt cycles. Stewart et al. (2012) found only weak and inconsistent evidence on microfinance (microcredit and microsavings) increasing engagement in economic opportunities, and consequently that microcredit (as well as combined microcredit‐microsavings) interventions had mixed and varied impacts on borrowers’ income; some studies suggested positive impacts, but these were prone to bias. Microsavings on its own appeared to increase incomes in some cases, but not in others. Steinert et al. (2018) found for Sub‐Saharan Africa that savings led to small but signiﬁcant increases in households’ expenditures and incomes, albeit with both outcomes pooled as a single variable (see discussion of “consumption” below).

##### 6.3.4.2 Assets

Another indication of poverty reduction would be the accumulation of more assets, increasing households’ net worth. Five meta‐studies examined this. Peters et al.’s (2016) review of qualitative evidence for microcredit, micro‐leasing, microsavings and micro‐insurance noted the high value that clients attached to owning assets, including nonproductive ones, such as home improvements, particularly for women's sociocultural life experience, and that clients often reported using credit to accumulate assets. However, they also reported client experiences of being forced to sell assets due to problems with repaying loans. Gopalaswamy et al.’s (2016) findings from meta‐analysis suggest that the overall effect of mixed microfinance, and particularly microcredit, on assets in South Asia is positive and statistically significant (and larger than its effect on income). Assets accumulated were mainly land and livestock. However, this finding is based on only very few (six) studies, with a high degree of heterogeneity, and it is not clear to what extent these assets were owned outright (net of debt). Steinert et al.’s (2018) meta‐analysis of savings promotion programmes in Sub‐Saharan Africa does not ﬁnd signiﬁcant impacts, across interventions, on asset ownership or housing quality (gpooled = 0.038, 95% CI [~0.01, 0.09]). Stewart et al. (2010) find for Sub‐Saharan Africa that microcredit and microsavings both have positive impacts on clients’ accumulation of assets, including their housing, but they also note a significant heterogeneity between clients, with some people being made poorer by microfinance, particularly microcredit clients. Stewart et al.’s (2012) review of access to economic opportunities found that the impact of microfinance (credit alone, or combined with savings) on the accumulation of non‐financial assets was mixed across countries; microsavings in some, but not all, cases helped savers to accumulate nonfinancial assets.

##### 6.3.4.3 Financial well‐being

Two meta‐studies explicitly conceptualised outcomes in terms of the financial well‐being of clients. Chliova et al. (2015) aggregated household consumer durables, consumption expenditure, income, poverty status (being under the poverty line or not) and diversiﬁcation of income sources as “financial well‐being”. Their meta‐analysis found the effect of microcredit on the ﬁnancial well‐being of entrepreneurs to be positive (*r* = .16, 95% CI [0.12, 0.21]). However, this finding was based mostly on (non‐experimental) studies with higher risk of bias. Duvendack et al. (2011) adopted a broader perspective on well‐being including, but not limited to, financial aspects. Their findings for the effects of mixed microfinance (mainly microcredit) suggest that no clear positive or negative conclusions can be drawn from the evidence, with only few economic impacts being reliably measured (see pp. 74–76).

##### 6.3.4.4 Consumption

Rising consumption levels (or rising household expenditure) may indicate different things. On the one hand, they could indicate higher incomes and the resultant ability to consume more; on the other hand, they could indicate unsustainable spending patterns fuelled by debt or driven by worsening circumstances. Without further information, it is thus unclear whether improvements or exacerbations are measured. Also, there are important differences in how credit versus savings may act to enable changes in consumption expenditure.

Four meta‐studies explicitly—and one implicitly, as part of “financial well‐being” (Chliova et al. 2015)—reviewed the evidence of financial inclusion initiatives on consumption levels. Chliova et al.’s (2015) finding of microcredit's effect on improved financial well‐being is only based to a small part (31 out of 214 observations) on consumption expenditure. The results of Gopalaswamy et al.’s (2016) meta‐analysis indicate that the effects of mixed microfinance interventions in South Asia were positive and statistically significant, but these estimates were strongly influenced by outliers. Microsavings for women had a significant impact in terms of raising expenditure, but only in Bangladesh, and with heterogeneous effects. Steinert et al.’s (2018) review of savings in Sub‐Saharan Africa found savings access to have small but signiﬁcant impacts on household expenditures, with larger effects for male savers than for women (which may be due to their background, rather than sex). They noted that expenditure increases enabled by savings accumulation, unlike by other financial services, must be seen as evidence of a household's financial improvement. For Sub‐Saharan Africa, Stewart et al. (2010) found both microcredit and microsavings to increase clients’ expenditure, but suggested that higher expenditure may also be a sign of clients consuming more instead of investing in their future. Stewart et al. (2012) found mixed and inconclusive evidence across countries of the effects of microcredit and microsavings on expenditure; while their evidence suggested microcredit in most cases tended to reduce expenditure, they noted that the advantages or disadvantages of this were not clear.

##### 6.3.4.5 Summary of evidence on economic outcomes

Overall, the effects of financial inclusion interventions, particularly microcredit and combined/mixed microcredit‐microsavings initiatives, on economic outcomes such as income or assets are positive but inconsistent and not particularly large. Credit and other financial services delivered through microfinance programming appear to have overall positive but decidedly mixed impacts, in terms of both lower‐ and higher‐order outcomes. The picture for microsavings looks more hopeful, suggesting small but more consistently positive effects, especially on savings accumulation and incomes (and not on non‐financial asset accumulation), and with fewer downsides for clients compared to credit. Having said that, Stewart et al. (2012) indicate that microsavings access does not enable the poor to engage in economic opportunities, but they also support the view that in some cases an increase in income, savings, expenditures and the accumulation of non‐financial assets is observable.

#### 6.3.5 Social outcomes

We have collected under the heading of social outcomes the gamut of beneficial outcomes that are not strictly economic or gender‐related. In the meta‐studies that we reviewed, these fell into three broad categories: social‐relational (strengthening of social ties, community bonds), health (physical health, nutrition, mental & psychological health), and access to beneficial services (such as water or schooling). It is difficult to distinguish any of these categories themselves as lower‐ and higher‐order outcomes, and rather there are pathways from lower‐ to higher‐order outcomes within each of them, as we point out below (and as shown in Figure 1, in the “Background” section).

##### 6.3.5.1 Social‐relational outcomes

Findings on social‐relational outcomes among the included meta‐studies were relatively few. Stewart et al.’s (2010) narrative synthesis of quantitative and qualitative evidence for Sub‐Saharan Africa found no studies measuring social cohesion. Brody et al. (2015) narrative review of qualitative evidence found that networking experiences in SHGs represented a signiﬁcant change for women from the domestic sphere and from speaking only to family and neighbours. They found high‐conﬁdence qualitative evidence for improvements in women SHG members’ speaking conﬁdence and feeling comfortable working with other stakeholders in their communities (as an aspect of interaction in groups, we take this to be a lower‐order outcome). The findings of improved cohesion are corroborated by Peters et al.’s (2016)’s finding from reviewing qualitative evidence on South Asian clients’ experiences that participation in various microfinance programmes helped women access the social support and solidarity of other women. The effect was more meaningful for programmes with stronger empowerment and solidarity‐building aspects (such as CBSGs and SHGs), and was only indirectly related to the financial service itself.

##### 6.3.5.2 Services

Six reviews assessed access to or usage of education facilities as an outcome (a lower‐order outcome, compared to educational results). Chliova et al.’s (2015) meta‐analysis of the effect of microcredit on business ventures found a small but positive and significant effect (*r* = .05, 95% CI [0.02, 0.08]) for the clients and their families, which they suggested may be partially offset by some detrimental effects of credit, such as removal of children from school for work. Gopalaswamy et al.’s (2016) meta‐analysis suggested a positive pooled effect size of various types of microfinance in South Asia on education, but the effect, similarly, was small, and more pronounced for girls. However, they noted contradictions in their evidence base, with their narrative synthesis finding impacts to be varied. Steinert et al.’s (2018) meta‐analysis found no effects of savings promotion on educational investment or school enrolment in Sub‐Saharan Africa. Stewart et al. (2010) found the evidence for the impact of microcredit and microsavings access on education to be varied, with limited evidence for positive effects and considerable evidence that microcredit could do harm. While they did not find evidence that microcredit increased child labour, they found that clients had difficulties paying school expenses, and some evidence that clients took children out of school. Similarly, Duvendack et al. (2011) found some evidence for mixed microfinance (mainly microcredit) having positive impacts on school enrolment overall, but only from less reliable sources, and no robust evidence for girl's enrolment. Peters et al.’s qualitative review of client experiences found that investing in children's future was a common motivator for joining various types of microfinance groups, and some evidence that clients used borrowed money for education or other child‐related expenses.

We found no reviewing regarding the impacts of financial services on access to other services, such as water, sanitation, or electricity.

##### 6.3.5.3 Health: physical

At least nine of the 11 meta‐studies in our sample made reference to physical health outcomes and behaviours, but only a subset of these explicitly assessed physical health‐related outcomes. Two focused exclusively on health: Orton et al. (2016) on the broader health impacts of group‐based microfinance that targeted women, and Kennedy et al. (2014) specifically on the HIV‐related impacts of microfinance and other income generation interventions for poor people. Orton et al. (2016) found an association between microfinance scheme membership (mainly delivering microcredit) and reduced infant mortality (a higher‐order outcome), however this was based on only two higher‐confidence studies in their review. Maternal health was found to improve, but this was inconsistent and based on lower‐confidence evidence. Evidence on nutritional status and the general health of women proved inconclusive, and measured improvements in empowerment did not clearly translate into health outcomes. Kennedy et al. (2014) found that evidence for how income generation interventions (most containing a microcredit element) affecting HIV‐related behaviours and outcomes was inconclusive. Their review had to rely on a moderately rigorous set of studies, of which few studies found significant effects on health behaviours (such as condom use and sexual risk‐taking). They found no data on HIV prevalence as the ultimate outcome. However, they did find moderate but significant improvements in accessing primary care for child health as well as knowledge about HIV and sexually transmitted infections (STI) (lower‐order outcomes) from those programmes that combined credit access with health education/messaging. For an aggregate of health and nutrition outcomes, Chliova et al.’s (2015) meta‐analysis found evidence of potential beneﬁcial effects from microcredit; however the effects were so small (*r* = .08, 95% CI [~0.04, 0.22]) that the authors suggested they might be partially offset by detrimental effects of credit. Steinert et al.’s (2018) meta‐analysis found no evidence for a health‐related effectiveness of savings programmes in Sub‐Saharan Africa, neither in terms of general health status nor health investments. Stewart et al. (2010) found microcredit and microsavings in Sub‐Saharan Africa had generally positive impacts on health, but with evidence extending mostly only to (lower‐order outcomes) health‐related behaviours rather than outcomes (higher‐order outcomes).

##### 6.3.5.4 Health: mental

Several reviews examined mental health and psychological well‐being effects under headings such as psychological empowerment, anxiety, stress, self‐confidence and self‐esteem; we would class these as higher‐order outcomes, because they are ends rather than means. However, none found strong or clear evidence of such effects. Brody et al. (2015) meta‐analysis of SHG programmes in South Asia found no evidence for positive eﬀects on psychological empowerment of women, and their narrative synthesis found some evidence of disappointment and frustration among women. Orton et al. (2016) found credit, as part of microfinance, could lead to increased anxiety due to repayment pressures. Gopalaswamy et al. (2016) suggested that SHGs and other types of microfinance participation led to increased self‐esteem, but without specifying the magnitude or mechanisms of such an effect. Given the experiential nature of mental states and psychological well‐being, Peters et al.’s (2016) review of qualitative evidence on client experiences was most instructive here. They find a wide variety of self‐worth‐enhancing and ‐reducing effects, depending on very different ways of being treated by (across and within) different programmes. Most importantly, they suggest greater self‐confidence results more from group membership and activities that promote learning, social engagement, and entrepreneurship, and less from using a financial service itself; stand‐alone financial services, rather, enhanced the risk of clients feeling isolated or manipulated.

##### 6.3.5.5 Health: nutrition

Nutrition is an important aspect and enabler of further health outcomes. Peters et al.’s (2016) qualitative review of user experiences in South Asia found users of various different types of microfinancial services (their review often does not distinguish the intervention type clearly) highlighted the importance of good diets and the ability to invest in the future of their children in relation to their (positive and negative) experiences of financial services usage. Chliova et al.’s (2015) pooled coefficient for microcredit's effect on health and nutrition was positive but very small (*r* = .08, 95% CI [~0.04, 0.22]) and was possibly offset by negative aspects of credit usage. Steinert et al.’s (2018) meta‐analysis found signiﬁcant increases in food security (*g* = 0.052, 95% CI [0.01, 0.10]) from savings promotion programmes in Sub‐Saharan Africa, while Stewart et al. (2010) found a generally positive but heterogeneous and inconsistent impact from microcredit and microsavings on food security and nutrition in the same region. For South Asia, however, Gopalaswamy et al. (2016) found no effect of microsavings on food consumption. Stewart et al.’s (2012) review found mixed (positive and negative) effects from microcredit and microsavings across countries and client groups.

##### 6.3.5.6 Summary of evidence on social outcomes

Overall, in comparison to the effects for economic outcomes, it appears that the effects for social outcomes are even smaller, or even more mixed.

#### 6.3.6 Gender outcomes

Microfinance programmes, particularly in South Asia, have a history of targeting women and claiming to bring about women's empowerment, but in theory, all financial services could affect gender relations in a number of complex and interrelated ways. We would not label these effects as lower‐order or higher‐order outcomes. To consider, for instance, women's independence and women's mutual support for one another as outcomes of a lower order than improved family planning or reduced domestic violence—which are likely to be enabled by the former, but in turn might also be enablers of it—, would involve a value judgment and assumptions about causality that we refrain from here. Therefore, in discussing gender outcomes (similarly to what we have done presenting the social outcomes), we make no distinction between lower‐ and higher‐order gender outcomes, and rather we report findings for several specific gender‐related impacts before reporting findings for women's empowerment more broadly.

All 11 medium‐ and high‐confidence meta‐studies took an interest in gender and women's empowerment in one way or another. Two meta‐studies, both including a meta‐analysis, explicitly focused on women's empowerment (Brody et al., 2015; Vaessen et al., 2014). They indicate different effects from microcredit versus SHG approaches.

##### 6.3.6.1 Family planning/sexual decision‐making

Several meta‐studies examined family planning, sexual self‐determination and sexual decision‐making as an outcome of using financial services. Brody et al. (2015) meta‐analysis of SHGs, using evidence from four studies, suggested a large but not statistically significant effect on women's family size decision‐making power (SMD = 0.26, 95% CI [−0.04, 0.56]) (their qualitative synthesis did not report on this outcome). In the context of HIV prevention, Kennedy et al. (2014) found no clear or consistent evidence for credit for income‐generation accompanies by health training/messaging having any significant effects on contraceptives usage, number of sexual partners, or other HIV‐related behavioural outcomes. Orton et al. (2016) found no consistent evidence across programmes that participation in group‐based microfinance (mainly credit) led to improvements in family planning. Gopalaswamy et al. (2016) found mixed and inconclusive evidence regarding fertility and contraceptives usage from participation various types of microfinance programme in South Asia.

##### 6.3.6.2 Domestic violence

No review found any clear evidence for or against access to financial services leading to increases or decreases in domestic violence and abuse. However, several studies indicated the effects (predominantly of credit access, in these studies) to be heterogeneous across different women and different contexts (Brody et al., 2015; Kennedy et al., 2014; Peters et al. 2016; Vaessen et al. 2014). Some, furthermore, suggested a sequential impact, with initially higher violence or abuse potentially being followed by lower levels, as women solidified empowerment gains (Brody et al. 2015 looking at SHGs, Kennedy et al. 2014 looking at credit for income‐generation). As Orton et al. (2016, p. 701) conclude: “while microfinance may eventually lead to a reduction in such violence, an initial increase may occur as gender norms are challenged”.

##### 6.3.6.3 Women's economic status

Improvements in women's economic status may be seen as a sub‐set and potential enabler of broader empowerment. Few meta‐studies explicitly assess it. Brody et al. (2015) defined women's economic empowerment as women's ability to access, own, and control resources. They found it was measured mostly in terms of women's bargaining power or decision‐making power in the household, mainly regarding decisions about expenditures and borrowing (leaving it unclear to what extent a woman taking a loan may already count as evidence of empowerment). On the basis of evidence from seven studies with medium and low risk‐of‐bias, their meta‐analysis found participation in SHGs in South Asia had a positive and statistically signiﬁcant eﬀect (SMD = 0.18, 95% CI [0.05, 0.31]); however, their analysis also showed strong heterogeneity in the impact estimates. Vaessen et al.’s meta‐analysis of microcredit and women's control over household spending found only a small positive and not clearly significant effect among medium risk‐of‐bias studies (SMD = 0.069, 95% CI [−0.003, 0.141]), and no effect among the low‐risk experimental studies, leading them to conclude that overall there was no evidence for a significant effect. Steinert et al. (2018) concluded that, in Sub‐Saharan Africa, savings promotion programmes do not seem to have the intended economic impacts on female recipients, and suggest that targeting women alone would not overcome negative intra‐household dynamics. Gopalaswamy et al. (2016) found mixed and inconclusive evidence on the effects of various types of microfinance in South Asia on employment for women. Chliova et al.’s (2015) study of credit for entrepreneurship suggests that the enablers of women's broader empowerment might not primarily be related to women's economic activity, and Peters et al.’s (2016) findings from qualitative studies of user experiences suggest that the women empowerment impacts of various microfinance (mainly microcredit) programmes came mainly from women gaining mobility and independence rather than from changes in their economic status.

##### 6.3.6.4 Women's empowerment (general)

Brody et al. (2015) meta‐analysis of SHG programmes in South Asia found that women's participation had positive eﬀects on their economic and political empowerment, mobility and control over family planning decisions. However, the poorest tended to be excluded from participating and benefiting. They further suggested that the main mechanisms of empowerment ran through women's increased familiarity in handling money, increased independence in ﬁnancial decision‐making, group solidarity‐building and social networks and respect gained within the household and the community. The eﬀect of participating on political empowerment, which Brody et al. (2015) defined as “women's ability to participate in decision‐making focused on access to resources, rights, and entitlements within community” was estimated to be positive and significant (SMD = 0.19, 95% CI [0.01, 0.36]); however, this was based on only two studies. The effect on women's mobility, similarly, was found to be positive and significant (SMD = 0.18, 95% CI [0.06, 0.31]), but this was based on evidence from only three studies. Brody et al. (2015) narrative synthesis of qualitative evidence, similarly, suggested high conﬁdence in ﬁndings that being an SHG member resulted in increased respect from community members, with women being more mobile and having greater self‐confidence. Their qualitative review findings also suggested that for some women participation in SHGs served as a stepping stone toward wider social participation, exposed them to women's rights, and gave them political capital through networking. Vaessen et al.’s (2014) meta‐analysis of experimental studies, however, found no evidence for impacts of microcredit on women's empowerment‐related variables, neither in individual studies nor when using pooled effect sizes (SMD = −0.007, 95% CI [−0.041, 0.027]). From non‐experimental studies, they found a small but positive and statistically significant effect (SMD = 0.129, 95% CI [0.035, 0.222]), albeit with a high degree of heterogeneity, and driven by two outlier studies. Overall, they found the evidence on women's empowerment to be confounded by the variety of contexts, types of microcredit service delivery, and different outcome variables, and concluded that there was no effect on women's control over household resources, in turn making it “very unlikely that microcredit has a meaningful and substantial impact on empowerment processes in a broader sense” (Vaessen et al. 2014, p. 67–68).

Among the non‐gender focused reviews, Chliova et al. (2015) found microcredit for enterprise having a positive effect on female empowerment (*r*  = .21, 95% CI [0.14, 0.27]), measured as women being allowed to make decisions regarding the purchase of assets, which they argued stands out among their results in terms of its magnitude. This indicates, they suggested, that microcredit participation triggers a virtuous cycle, starting with microcredit groups assigning women responsibilities and rights, and leading to repeated social and economic interactions, which could confer power onto women and facilitate the joint pursuit of common interests. However, to the contrary, Duvendack et al.’s (2011) global review of the impacts of microfinance (predominantly microcredit) found no robust evidence of positive impacts overall on women's empowerment. In South Asia, Gopalaswamy et al.’s (2016) meta‐analysis of mixed microfinance types found an overall positive but very small and marginally significant effect on women's empowerment, conceptualised as the decision‐making power of women (SMD = 0.028, 95% CI [0.005, 0.052]). The findings underlying their analysis are highly heterogeneous (with positive and negative and usually insignificant results) and are sensitive to the definitional parameters used. They found no difference between the impacts of microcredit versus broader microfinance programmes. For Sub‐Saharan Africa, Stewart et al. (2010) found some evidence that microcredit empowers women, however, it was not consistent across studies, and empowerment outcomes were poorly and inconsistently measured. Orton et al.’s (2016) review of the health effects of SHGs found that membership in relatively large and well‐established schemes generally led to increased women's empowerment, but this did not necessarily translate into improved health outcomes. Peters et al.’s (2016) review of qualitative evidence also suggested women in South Asia were more likely to experience empowerment from participating in programmes that promoted community‐building and social engagement, and which required them to leave their household and move about in the community, rather than from stand‐alone financial services.

##### 6.3.6.5 Summary of evidence on gender outcomes

The effects of financial inclusion interventions on women's empowerment appear to be positive on the whole, albeit relatively small. The effects heavily depend on programmatic features of the interventions, with several meta‐studies raising the question to what extent financial services themselves, rather than other programme elements, such as exposure to women's rights, awareness‐raising, or efforts at group‐building and social networking (which may be independent from any financial intervention) explain the effects. The effects of specifically gender‐targeted programme elements were larger than those of the actual financial service (Chliova et al., 2015; Peters et al. 2016). The main enablers of empowerment effects appear to be group interactions, opportunities to leave the house, and exposure to additional rights‐related training, rather than financial services. The effects also depend on contextual circumstances, as several studies note (Peters et al. 2016; Vaessen et al., 2014) such as existing gender norms, and are often restricted to particular aspects of women's empowerment—as also described in other literature referring to, for example, participation in household decision‐making over use of financial services (e.g., Kabeer, 2001), control over assets (e.g., Goetz & Sen Gupta, 1996), physical mobility, political and legal awareness of women as well as their participation in public protests or political campaigns (e.g., Hashemi & Schuler, 1994). The challenge of obtaining uncontested empowerment impacts is moreover hampered by difficulties of conceptualising and measuring women's empowerment. As discussed by Vaessen et al. (2014), primary studies assessing the impact of financial inclusion interventions on women's empowerment often reach different conclusions due to diverging methodologies, concepts and measures of empowerment (Kabeer, 2001), making it difficult to reach any generalisable findings on the impact of financial inclusion on women's empowerment.

#### 6.3.7 Behavioural outcomes

It has been suggested that access to financial services, especially services that contain particular modalities to affect their users’ behaviour, leads to the development of various potentially desirable cognitive capabilities and behavioural changes. In theory, changes in behaviours and cognitive capacities could come from several factors. Firstly, changes in financial knowledge and abilities could directly come from financial literacy programmes (which we deemed beyond the scope of this review, as not being directly part of financial services, but rather training for readiness) or through experience gained over time in using money and financial services. Particular financial products might, as a by‐product of their usage, change users’ money‐usage patterns over time, for instance leading to higher propensities to save, more investment in business, or less spending on particular goods such as “temptation goods” (Banerjee et al., 2015). It has also been suggested that specially designed financial products could help poor people overcome behavioural or cognitive constraints or attitudes that worsen their poverty and keep them in poverty; for instance when “commitment” savings devices that commit people to longer‐term goals and help them overcome possible biases toward present enjoyment.

We were surprised to find a general lack of evidence for and relatively little attention paid to behaviour‐related outcomes among the meta‐studies we reviewed, not least given the attention that behavioural thinking has garnered in recent years in development research and policymaking in general (Klein, 2017; World Bank, 2015) and in discussions of financial inclusion in particular (Karlan et al., 2014). Relatively few meta‐studies explicitly discussed behavioural changes, and none focused on them. However, a number of meta‐studies sought to assess changes in spending and saving patterns, financial knowledge and capability, and propensity to engage in enterprise.

We treat all behavioural outcomes as lower‐order outcomes, because they ought not to be seen as ends in themselves. They would indicate, if found, merely the potential for poverty‐reducing or transformative effects to happen further along the causal chain.

##### 6.3.7.1 Spending and saving patterns

The evidence found for changes to consumption and expenditure patterns should be seen in light of the relatively weak and unclear evidence on changes to overall consumption levels, as well as the ambiguous nature of changes to consumption and expenditure, noted above. Even if the evidence did not suggest any consistent or substantial changes to expenditure *levels* to result from financial inclusion, beneficial changes to spending *composition* may nonetheless represent an important effect at an early stage in the impact chain, and may be an enabler of eventual transformative economic impacts. However, the reviewed evidence does not suggest this to be the case. The evidence found is mixed, inconsistent, and heterogeneous. In South Asia, Gopalaswamy et al. (2016) found inconsistent and unclear patterns of changes to spending composition from access to various microfinance programmes. Stewart et al. (2012) found some evidence of composition changes, albeit mixed and inconsistent, for instance evidence that microsavings significantly increased spending on food and personal items, such as alcohol and clothing, in Kenya, but in Bosnia and Herzegovina microcredit had no effect on business spending and led to a significant decrease in consumption of food at home among entrepreneurial clients; combined microcredit and microsavings in India appeared to have the effect of increasing spending on housing and consumer goods, but not on food. Stewart et al. (2010) noted that patterns of consumption change in Africa, which microcredit may cause, could in fact indicate clients becoming poorer: some clients consumed more instead of investing in their futures. Steinert et al.’s (2018) meta‐analysis of savings found no support for the hypothesis that ‘‘tying one's hands” with behavioural constraints (such as purpose‐labelled accounts, peer pressure, or institutionalised withdrawal restrictions) changed the effectiveness of savings programmes.

##### 6.3.7.2 Financial knowledge and capability

Financial knowledge and capability were not a focus of this review, which was focused on poverty impacts (we excluded meta‐studies that focused exclusively on these as outcomes of interest). Yet relevant changes in these might still count as early‐stage effects on a trajectory towards poverty alleviation. The reviewed reviews, however, turned up few relevant clear results. Steinert et al.’s (2018) meta‐analysis of savings promotion in Sub‐Saharan Africa found that programmes that actually delivered savings services were significantly more effective at raising savings than those seeking to change savings‐related attitudes, build financial literacy or raise demand for savings services. While they found a trend towards increases in ﬁnancial literacy levels, heterogeneity levels were high (gpooled = 0.12, 95% CI [~0.01, 0.24]), and demand‐based programmes, namely ones that focused on ﬁnancial literacy and ﬁnancial education, were not associated with signiﬁcant changes in immediate or more transformative poverty‐related outcomes. For SHGs in South Asia, Brody et al. (2015) found high‐conﬁdence results that suggested women reported feeling empowered by the newness of handling money, but also that women in six studies reported feeling unsure about ﬁnancial decisions despite receiving training, and sometimes felt pressured by others and unprepared to take financial decisions.

##### 6.3.7.3 Entrepreneurship propensity

No strong or clear findings regarding entrepreneurship propensity emerged from the reviewed literature. As mentioned above (under economic outcomes), effects of financial service access on venture growth and survival were mixed and small, where present. Chliova et al.’s (2015) meta‐analysis of microcredit for enterprise found some evidence of enterprise growth, but did not discuss the propensity to start an enterprise. Stewart et al. (2012) found no evidence that microsavings had an effect on enterprise, and some evidence that microcredit influenced poor people's engagement in enterprise, however most of this evidence came from less‐reliable sources.

##### 6.3.7.4 Summary of evidence on behavioural outcomes

Behaviour‐changes could be enablers of more transformative changes. However, we found little evidence for behaviour‐related changes among the meta‐studies. A number of intricate and complex findings emerged, but none suggested consistently significant positive or negative changes. Effects on of credit and savings products on spending and saving patterns were mixed, inconsistent, and heterogeneous, and no evidence showed commitment devices improving the impact of savings interventions. Financial literacy and ﬁnancial education programmes for changing savings behaviours had no significant effect on poverty‐related outcomes. No strong or clear evidence was found for financial inclusion interventions enhancing entrepreneurship propensity. None of the meta‐studies presented evidence on behavioural outcomes that could be categorised as enabling higher‐order or final outcomes.

#### 6.3.8 Summary of findings

To help with the transition from descriptively synthesising the findings of the 11 medium‐ and high‐confidence reviews to discussing the implications of these findings for policy and practice, we draw on GRADE to structure the qualitative and quantitative evidence we highlighted in the sections above. Table 9, below, summarises the findings for specific outcome sub‐categories, clarifying the financial inclusion intervention category, and the confidence level of the meta‐study reporting the outcome, and the number of studies included in that meta‐study that reported a particular outcome.^27^

**Table 9:** Summary of findings

Outcome	Meta‐studies	Confidence of the meta‐study	Financial inclusion category	Direction	Meta‐analysis	Specific outcome	Sample size (No. of studies)	Effect size	Confidence Interval (CI 95%)		Type of effect size
**Economic**											
*Lower‐order/intermediate outcomes*											
Savings amount	Stewart et al. (2012)	Medium	Microfinance in general	Inconclusive	No						
	Stewart et al. (2010)	Med‐low	Microcredit & microsavings	Positive	No						
	Steinert et al. (2018)	High	Microsavings	Positive	Yes	Savings balance	18	0.077	0.03	0.12	SMD
				Insignificant		Propensity to save	4	0.061	−0.02	0.09	SMD
*Higher‐order/final outcomes*											
Assets/wealth	Gopalaswamy et al. (2016)	Med‐low	Microfinance in general	Positive	Yes	Financial assets	6	0.258	0.093	0.425	SMD
	Stewart et al. (2012)	Medium	Microfinance in general	Inconclusive	No		3				
	Stewart et al. (2010)	Med‐low	Microcredit & microsavings	Positive	No		17				
	Steinert et al. (2018)	High	Microsavings	Insignificant	Yes	Housing assets	9	0.038	−0.01	0.09	SMD
				Insignificant		Lumpy' investment	9	0.045	0.00	0.09	SMD
Income	Gopalaswamy et al. (2016)	Med‐low	Microfinance in general	Insignificant	Yes		11	0.067	−0.093	0.226	SMD
	Chliova et al. (2015)	Med‐low	Microcredit	Positive	Yes		6	0.11 (0.22)	0.02 (0.04)	0.19 (0.39)	PCC (SMD^**^)
	Stewart et al. (2010)	Med‐low	Microcredit & microsavings	Inconclusive	No		5				
	Steinert et al. (2018)	High	Microsavings	Positive	Yes	Microenterprise profits	7	0.044	0.02	0.07	SMD
				Positive		Wage work income	11	0.066	0.02	0.12	SMD
**Social**											
Services: education	Gopalaswamy et al. (2016)	Med‐low	Microfinance in general	Positive	Yes		5	0.044	0.015	0.072	SMD
	Stewart et al. (2010)	Med‐low	Microcredit & microsavings	Inconclusive	No						
	Steinert et al. (2018)	High	Microsavings	Insignificant	Yes	School enrolment	3	0.06 (0.11)	−0.18 (0.33)	0.30 (0.54)	OR (SMD*)
	Chliova et al. (2015)	Med‐low	Microcredit	Positive	Yes		24	0.05 (0.10)	0.02 (0.04)	0.08 (0.16)	PCC (SMD^**^)
Health: nutrition	Stewart et al. (2010)	Med‐low	Microcredit & microsavings	Positive	No						
	Chliova et al. (2015)	Med‐low	Microcredit	Positive	Yes	Health & nutrition	42	0.08 (0.16)	0.04 (0.08)	0.11 (0.22)	PCC (SMD^**^)
	Orton et al. (2016)	Med‐low	Microcredit	Inconclusive	No						
Health: physical	Stewart et al. (2010)	Med‐low	Microcredit & microsavings	Positive	No						
	Orton et al. (2016)	Med‐low	Microcredit	Positive	No						
**Gender**											
Women's social status	Brody et al. (2015)	Med‐high	Self‐help groups	Insignificant	Yes	Women's family size decision making	6	0.25	−0.03	0.54	SMD
				Positive	Yes	Women's mobility	3	0.18	0.06	0.31	SMD
	Vaessen et al. (2014)	High	Microcredit	Positive	Yes	Women's control over HH spending in Bangladesh	6	0.124	0.021	0.226	SMD
				Insignificant	Yes	Women's control over HH spending elsewhere	8	0.013	−0.057	0.082	SMD
Women's empowerment	Gopalaswamy et al. (2016)	Med‐low	Microfinance in general	Positive	Yes		6	0.028	0.005	0.052	SMD
	Stewart et al. (2010)	Med‐low	Microcredit & microsavings	Inconclusive	No						
	Chliova et al. (2015)	Med‐low	Microcredit	Positive	Yes		26	0.21 (0.43)	0.14 (0.28)	0.27 (0.56)	PCC (SMD^**^)
	Kennedy et al. (2014)	Med‐low	Microcredit	Inconclusive	No						
	Brody et al. (2015)	Med‐high	Self‐help groups	Insignificant	Yes	Women's psychological empowerment	2	0.02	−0.21	0.26	SMD
				Positive	Yes	Women's political empowerment	2	0.19	0.01	0.36	SMD
				Positive	Yes	Women's economic empowerment	7	0.18	0.05	0.31	SMD
				Insignificant	Yes	Domestic violence	2	0.07	−0.06	0.2	SMD
**Behavioural**											
Health behaviour	Kennedy et al. (2014)	Med‐low	Microcredit	Inconclusive	No						
	Orton et al. (2016)	Med‐low	Microcredit	Positive	No						
Spending patterns	Steinert et al. (2018)	High	Microsavings	Insignificant	Yes	Education investment	6	0.01	−0.03	0.05	SMD
				Insignificant	Yes	Health investment	5	0.01	−0.01	0.03	SMD

*Notes*: Brody et al. (2015): Effect sizes correspond to RCT and medium risk of selection bias quasi‐experimental studies. This table has been adapted from Waddington et al. (2014) and is inspired by GRADE.

* SMD calculated from log‐odds ratio using Cox transformation.

^**^ SMD calculated from correlation coefficient (formulae in Polanin & Snilstveit, 2016).

Five (e.g., Brody et al., 2015; Chliova et al., 2015; Gopalaswamy et al., 2016; Steinert et al., 2018; Stewart et al., 2010) out of the 11 reviewed meta‐studies drew largely positive conclusions (with exception for some outcomes, where insignificant or inconclusive effects were found) about the relationship between financial services access and changes for poor people. The other six (e.g., Duvendack et al., 2011; Kennedy et al., 2014; Orton et al., 2016; Stewart et al., 2012; Peters et al., 2016; Vaessen et al., 2014) drew largely mixed, neutral, or unclear conclusions (as summarised in Tables 8 and 9).

The detailed review of the evidence above uncovered an even more nuanced picture, reflecting large variations across the effects of different interventions (credit only, savings only, community‐based finance, mixed microfinance) and for different people in different contexts. Findings across the meta‐studies were heterogeneous and often inconsistent, both within and across meta‐studies, and many did not find evidence of expected or presumed impacts.

Overall, we find:

- Financial services do have some impacts on poor people's lives, and these are more likely to be positive than negative. But the impacts vary, they are often mixed, and they appear not to be transformative in scope or scale. The effects tend to occur in the early stages of the causal chain.
- The effects of financial services on core economic poverty indicators such as incomes, assets or spending are small and inconsistent.
- The effects of participating in programmes that deliver financial services on women's empowerment appear to be generally positive, but they depend upon programme features (often only peripheral or unrelated to the financial service itself, for instance exposure to women's rights), context (such as social norms), and on which aspects of empowerment are considered. The assessment of gender impacts is confounded by a difficulty of consistently conceptualising and measuring empowerment (across meta‐studies and across underlying studies).
- The effects of credit and other financial services on health status and other social outcomes appear to be small or non‐existent.
- There is no evidence for meaningful behaviour‐change outcomes leading to further positive effects.
- Accessing savings opportunities appears to have small but much more consistently positive effects for poor people, and logically and empirically entails fewer downside risks for clients than credit.

The bulk of the directional findings reported by the narrative syntheses and meta‐analyses regarding impacts are positive, with few negative ones. Whether the reasons for this are a dominance of positive effects or a dominance of *reporting* of positive effects is not fully clear (e.g., two of the meta‐studies made it clear that they could not rule out publication bias; see Gopalaswamy et al., 2016; Steinert et al., 2018). We note that meta‐studies, generally, tend to focus on reporting the (few) impacts that they find, or highlight these much more strongly in their conclusions, than on highlighting non‐findings and the (often very large) gaps in their evidence base. In some cases, we believe this has entailed reviewers paying less attention to the to the problems of their small and inconsistent effect sizes and/or the unreliability of their evidence bases in terms of quality, in favour of drawing vaguely positive (and positively vague) conclusions.^28^ In other cases, however, authors have been transparent and reflective about non‐findings, smallness of effect sizes, and about articulating their doubts regarding the evidence base, and thus managed to draw higher‐quality and more helpful conclusions.^29^

Importantly, even where we assessed meta‐studies as being high confidence, that does not mean that their underlying evidence base was of a high quality standard. Very many of the underlying studies have “medium” or even high risk of bias, as reported by the meta‐studies (and as reflected upon by several), due to their study design, poor reporting of methodology and other causes. Consequently, we have the choice, as Stewart et al. (2012, p. 55) note, between dismissing the bulk of evidence, or seeking to establish what constitutes “good‐enough evidence”. Some of the meta‐studies we reviewed paid great attention to differentiating their findings according to the reliability of evidence; others less so. Our capacity in this systematic review of reviews to look “through” the meta‐studies to assess the reliability of their underlying primary evidence base has been limited (and indeed, doing so systematically would defeat the purpose of a systematic review of reviews). As several of the meta‐studies highlight (and we feel some should have more clearly considered), it was mainly higher‐risk‐of‐bias studies that drove most of the positive impact estimates (as discussed in greater depth above). Our findings thus broadly confirm “Rossi's stainless steel law of evidence” (as observed by Peter Rossi^30^ and adapted by Petticrew, 2003) that the more rigorous and lower‐risk of bias studies are, the less likely they are to find effects. This applies to both our reviewed meta‐studies and to the underlying studies that constituted their evidence base. Given that the reviews we classified as being of medium‐to‐low confidence were more likely to report positive incomes, we must treat their positive findings with greater caution. Brody et al. (2015) and Steinert et al. (2018) are exceptions, being high‐confidence meta‐analyses that report relatively positive findings, though they make it very clear that their findings apply to only particular sub‐types of financial service delivery (SHGs and savings), reflect on adverse effects, and also clearly differentiate their findings from their non‐findings.

Looking across the meta‐studies, almost all effect sizes are quite small—based on a small sample of meta‐analyses (*n *= 5) capturing only 76 effect sizes across 15 very diverse outcomes —and are hardly indicative of transformative changes from financial inclusion, as dominantly lower‐order outcomes are affected. Many effects are strongly heterogeneous, both across studies and over time, places, populations, gender and ethnicity as well as between interventions^31^; this suggests them to be unreliable and/or context‐dependent. Positive findings tend not to repeat from one context, intervention‐type or study to another, and at least as many findings are mixed or inconclusive as are positive. Consequently, the positive results found for financial inclusion are fragile, and need to be treated with caution.

It is crucial, finally, to note how most of the positive effects found are on outcomes that are early in the causal chain; for instance, meta‐studies of health‐focused interventions find most positive changes in health knowledge, few in health behaviours, and none in health outcomes. In other cases, positive effects are found for growth in business ventures, but not in household incomes as a subsequent result. An exception appears to be with regards to savings, where both immediate outcomes and wider poverty measures were affected in a positive, but relatively small, way, however we base this mainly on the findings of one high‐confidence meta‐analysis (Steinert et al., 2018); while there is little evidence of a savings “revolution” (Ashe & Neilan, 2014), at least the evidence shows savings to do some good and no harm. The design of most studies underlying the meta‐studies that we reviewed has not been conducive to establishing whether short‐term or immediate outcomes (such as financial knowledge or entrepreneurial propensity) translate into intermediate outcomes (such as savings accumulation or microenterprise income) and especially more distal, transformative outcomes (higher net worth or higher incomes).

## 7 DISCUSSION

### 7.1 Summary of main results

We initially identified 32 eligible meta‐studies (systematic reviews and meta‐analyses) examining the impact of financial inclusion interventions on a range of economic, social, gender and behavioural outcomes. After subjecting these to a quality appraisal process, we excluded 21 reviews due to quality concerns, leaving a core sample of 11 medium‐ and high‐confidence meta‐studies.

As we can see from Table 10 below, reviews examining credit and savings interventions in relation to economic outcomes dominate the 11 medium and high confidence meta‐studies. A large number of outcomes were reported for mixed microfinance interventions, meaning ones that may have offered both savings and credit or further services (via different provision modalities, e.g., MFIs or SHGs), but not always linking specific interventions to outcomes. We found a relative shortage of reporting of behavioural and social outcomes.

**Table 10:** Linking outcomes (number of outcomes reported) and interventions for 11 medium and high confidence meta‐studies

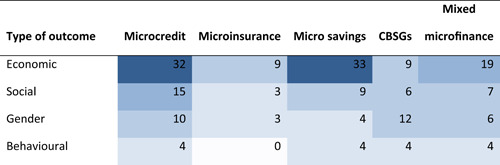

In a nutshell, across these 11 medium and high confidence meta‐studies, we find:

- Financial services do have some impacts on poor people's lives, and these are more likely to be positive than negative. But the impacts vary, they are often mixed, and they appear not to be transformative in scope or scale. The effects tend to occur in the early stages of the causal chain.
- The effects of financial services on core economic poverty indicators such as incomes, assets or spending are small and inconsistent.
- The effects of participating in programmes that deliver financial services on women's empowerment appear to be generally positive, but they depend upon programme features (often only peripheral or unrelated to the financial service itself, for instance exposure to women's rights), context (such as social norms), and on which aspects of empowerment are considered. The assessment of gender impacts is confounded by a difficulty of consistently conceptualising and measuring empowerment (across meta‐studies and across underlying studies).
- The effects of credit and other financial services on health status and other social outcomes appear to be small or nonexistent.
- There is no evidence for meaningful behaviour‐change outcomes leading to further positive effects.
- Accessing savings opportunities appears to have small but much more consistently positive effects for poor people, and logically and empirically entails fewer downside risks for clients than credit.

For the 21 excluded studies, due to the low confidence in their findings, we do not include the directions of reported outcomes in our synthesis. However, we would suggest that knowing the patterns of outcome reporting in these other studies can be useful for the design of future, higher‐confidence meta‐studies that complement the existing medium‐ and high‐quality evidence base. We note that the picture regarding types of outcomes reported is not very different for these low confidence studies as we can see from Table 11, again with an emphasis on economic outcomes and a relative paucity of reporting of social and behavioural outcomes. However, insurance and CBSGs dominate more strongly among the low confidence studies.^32^ A similar share of the effects were reported for “mixed” microfinance as among the included medium‐ and high‐confidence studies.

**Table 11:** Linking outcomes (number of outcomes reported) and interventions for 21 low confidence meta‐studies

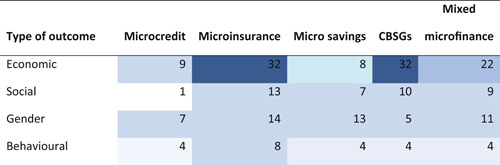

It is important to note, however, that the evidence base for both low and medium/high confidence studies is highly heterogeneous in terms of focusing on different intervention types, outcomes and geographies. As for the 11 medium and high confidence studies, many of the effects we find being reported positive, but often very small and occurring early on in the causal chain, which, if these meta‐studies had a higher confidence level, would similarly suggest a lack of long‐lasting and transformative changes.

As discussed above, the results of our narrative synthesis of the effects reported in 11 meta‐studies raise the question whether financial inclusion interventions are the most appropriate way forward in terms of poverty alleviation compared to other, potentially more cost‐effective or impact‐generating alternatives. These could be, for instance, graduation or livelihoods programmes. We have also shared quality concerns in relation to the meta‐study and primary study evidence base, and discussed the implications of small sample bias, which further caution the reader to place too much faith in the small and positive effects that we can report from reviewing these meta‐studies.

#### 7.1.1 The impact of financial inclusion interventions versus graduation and livelihoods programmes

Given the fragmented evidence base on the impact of financial inclusion interventions, it is worth considering the impact of potentially comparable alternatives, to understand whether there are alternatives with the potential to complement or to substitute financial inclusion activities in certain contexts. Hence, for comparison, we unsystematically (because a full systematic review of these would far exceed the scope of our review) sought to assemble a comparable evidence base of meta‐studies for livelihoods and graduation programmes. We argue that these interventions are comparable to financial inclusion interventions in terms of also having similar objectives of poverty alleviation and women's empowerment through directly working with poor people and seeking to increase their economic welfare and opportunities. However, livelihoods and graduation interventions often cast their net wider, in terms of the types of activities offered and overall outcomes that are targeted, and particularly livelihoods interventions are more heterogeneous. They also often do not have the same cost‐covering or profit‐making aims as financial inclusion activities.

We embarked on this exercise by searching the same bibliographic databases we used to identify the financial inclusion studies; we also searched the website of BRAC, which we identified as the leading organisation for graduation programmes. We found 17 relevant meta‐studies (systematic and unsystematic reviews) and nine impact evaluations on livelihoods and graduation programmes. These can be found listed below, in the “References” section, under “Additional references”.

**Figure 11:**
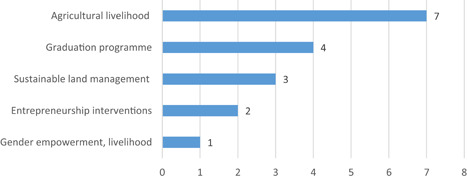
Intervention types across reviews. Note: This figure is based on the following 17 reviews: Banerjee et al., 2015; Blackmore, Lesorogol, & Iannotti, 2018; Blundo Canto et al., 2018; Bowler et al., 2010; Cho & Honorati, 2013; Devereux, Roelen, Sabates, Stoelinga, & Dyevre, 2015; Dickson & Bangpan, 2012; Halder & Mosley, 2004; Hemming et al., 2018; Higgins, Balint, Liversage, & Winters, 2018; Juillard, Mohiddin, Péchayre, Smith, & Vince, 2016; Liu & Kontoleon, 2018; Loevinsohn, Sumberg, Diagne, & Whitfield, 2013; Stewart et al., 2015; Sulaiman, 2016; Ton et al., 2013; Ton, Desiere, Vellema, Weituschat, & D'Haese, 2017. [Color figure can be viewed at wileyonlinelibrary.com]

**Figure 12:**
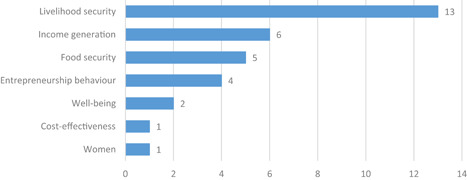
Outcome types across reviews.Note: Number and types of outcomes identified across 17 reviews: Banerjee et al., 2015; Blackmore et al., 2018; Blundo Canto et al., 2018; Bowler et al., 2010; Cho &d Honorati, 2013; Devereux et al., 2015; Dickson & Bangpan, 2012; Halder & Mosley, 2004; Hemming et al., 2018; Higgins et al., 2018; Juillard et al., 2016; Liu & Kontoleon 2018; Loevinsohn et al., 2013; Stewart et al., 2015; Sulaiman, 2016; Ton et al., 2013; Ton et al., 2017. [Color figure can be viewed at wileyonlinelibrary.com]

Unsurprisingly, the reviews that we identified cover a wide brief in terms of intervention types (see Figure 11 above). The nine impact evaluations all focused on graduation programmes. Thus, it is not surprising to find a wide range of outcomes and impacts. Figure 12 indicates that many of the livelihood and graduation programmes discussed in the reviews we found focus on livelihood security but also on income generation—an outcome that they have in common with financial inclusion programmes. Similarly, the nine impact evaluations largely focus on well‐being as the main outcome of interest, followed by income generation.

Nine of the 17 reviews reported positive impacts across a wide range of outcomes, while three reported largely positive but also some mixed impacts, and five reported only mixed impacts. Six of the 17 reviews focus on income generation and three of those found positive and one positive‐mixed evidence while two found mixed evidence. The nine impact evaluations covering graduation programmes are dominated by randomised controlled trials (RCTs) but also some quasi‐experiments, finding, similar to the reviews, positive (four studies), positive and mixed (two studies) and mixed impacts (three studies).

It is important to note that we find high levels of heterogeneity within livelihood and graduation type of activities across the reviews and impact evaluations. Furthermore, context may very much drive the difference in effects. Geographically, the studies we identified were widely dispersed and the quality of the evidence also needs to be taken into consideration to be able to assess the reliability of their findings. However, examining these issues in greater depth is beyond the scope of this review. We concluded from this brief review that the picture is similarly mixed and heterogeneous for graduation and livelihood programmes. We also note that, at least in the case of graduation approaches, there is a clear evidence gap, with no meta‐study having yet been done.

Our unsystematic and brief review of the livelihoods and graduation programmes only skims the surface of potential alternatives to financial inclusion interventions. It would also be worth exploring the cost effectiveness of financial inclusion interventions vis‐à‐vis livelihoods and graduation programmes or additional alternatives; but this may open up another “can of worms” altogether, which we cannot engage with here, and would first require similar levels of evidence synthesis between the interventions to have been attained.

Going forward, it would be worth trying to grapple with the high levels of heterogeneity within livelihoods and graduation programmes to better understand what drives their impacts and how they may be best integrated with, or promoted instead of, financial inclusion interventions, to enhance and harness the limited impacts we observe in financial inclusion. There may also be other alternatives worth investigating, such as social safety net programmes.

The point is that the alternative to financial inclusion is not “do nothing”, or to prioritise financial inclusion over the delivery of other services and forms of assistance, but rather to uncover what works best for whom and where, and how best to deliver it. Overall, a more open and clear‐sighted discussion in the policy and research space is needed on the many valid alternatives to financial inclusion programming and on how best to gain the necessary evidence to inform that discussion.

### 7.2 Overall completeness and applicability of evidence

During the in‐depth synthesis of the 11 medium‐ and high‐confidence meta‐studies, a number of evidence gaps became apparent, the largest of which we list below:

- None of the meta‐studies we reviewed assessed debt levels or indebtedness patterns. While some reviews reflected in their discussion of results that expanded access to credit can lead to vicious cycles of debt (Stewart et al., 2010, 2012) or reviewed clients’ negative perceptions of debt, none of the reviews assessed debt levels or trajectories as an outcome of financial inclusion. (None of the 21 low‐confidence meta‐studies made debt or indebtedness patterns their focus, either.) Debt remains the Achilles’ Heel of the financial inclusion sector and yet is one of its least systematically studied facets (Guérin et al., 2013).
- We found no evidence on the service/amenities‐related impacts of financial inclusion (beyond education), for example, water credit, sanitation loans, or loans for micro solar systems, which have grown rapidly in recent years. Especially the notion of “Green Microfinance”, where microfinance is applied to promote environmental sustainability moving beyond alleviating poverty and empowering women, has not been explored in any meta‐studies, even though this has been an area of growth increasingly receiving attention from policymakers. (Again, this applies to both higher‐ and lower‐confidence meta‐studies.)
- We also found no evidence for the claim that financial inclusion interventions lead to macroeconomic development and thus in turn improve the lives of the poor in low‐ and middle‐income countries. None of the studies in our sample (low‐, medium‐ or high‐confidence), examined the causal link between the development of an inclusive financial sector and economic growth. It is best to abandon this assertion until reliable evidence is found.
- Given that the majority of financial inclusion impacts are found at the early stages of the causal chain (e.g., see Table 9), there is a need for studies to capture long‐term effects to demonstrate more meaningful impacts especially at the final stages of the causal chain. The vast majority of the studies underlying the meta‐studies we reviewed in‐depth had a duration of 1–3 years. These studies are likelier to find changes in behaviours or attitudes rather than structural changes to people's poverty status, and it is not safe to assume that the latter will result from the former. The design of most studies underlying the meta‐studies that we reviewed has not been conducive to establishing whether short‐term or immediate outcomes (such as financial knowledge or entrepreneurial propensity) translate into intermediate outcomes (such as savings accumulation or microenterprise income) and especially more distal, transformative outcomes (higher net worth or higher incomes).
- We also need more meta‐studies that make more of an effort to understand impact heterogeneity. In other words, few of the meta‐studies we reviewed in depth successfully unpacked the drivers of heterogeneity of financial inclusion impacts, that is, impacts are not sufficiently disaggregated by gender, ethnicity, poverty status, and so forth and thus we still do not know for whom financial inclusion does or does not work, and why (not).
- We found a lack of high‐confidence systematic reviews or meta‐analyses of (micro)insurance products, despite an underlying rigorous study base already existing. Stewart et al. (2012) attempted to include microinsurance in their review, but found the insurance services to be too recent to have an adequate evidence base. We found five meta‐studies on insurance, including ones focused on on the effectiveness of index‐based weather insurance (Cole et al., 2012; Marr et al., 2016), and of micro health insurance (Bhageerati et al., 2017; Habib, Perveen, & Khuwaja, 2016; ). However, all five were ranked low confidence during the assessment of methodological quality and risk of bias. The evidence regarding the impact of the different types of insurance offered to poor people in financial inclusion programming would be an opportunity for a high‐quality, up‐to‐date systematic review or meta‐analysis, potentially by upgrading some of the more comprehensive existing efforts in this field.^33^

### 7.3 Quality of the evidence

In Section 6.2, we extensively discussed the quality of the included meta‐studies as well as the quality of the primary evidence that informed the meta‐studies.

At the level of meta‐studies, we had to discard, on quality grounds, from our in‐depth review two‐thirds of the studies that we had initially included. Our quality appraisal was rigorous, as we combined two reputable tools and had them applied independently by a group of RAs and the results checked by the lead review authors.

We should note that the AMSTAR 2 tool and 3ie quality appraisal checklist suffer from a certain degree of residual subjectivity, and the results of applying them were contradictory in the case of a few studies. It is also important to point out that only because a meta‐study has been categorised as low confidence, this does not mean that it cannot substantially add meaningful evidence to the knowledge base on the impact of financial inclusion interventions; it means that it did not meet (or report on) the rather stringent technical and procedural requirements that would have made it a medium or high confidence systematic review or meta‐analysis according to AMSTAR 2 and the 3ie checklist.

At the primary study level, 7 of the 11 medium‐ and high‐confidence meta‐studies stressed the often low quality of the evidence base they had included. Despite quality concerns, however, many low quality primary studies were included in the review process, which in turn raises concerns about the reliability of the overall findings presented in the meta‐studies we included, in particular where no mitigating actions were taken, for example, disaggregating the synthesis of findings by risk of bias level. As argued above, combining a wide range of low quality studies into systematic reviews is unhelpful and potentially risky; we would argue it is analogous to the repackaging of poor‐quality assets by financial institutions into larger‐volume triple A products as observed in the lead up to the 2008 financial crisis.

### 7.4 Limitations and potential biases in the review process

To our knowledge, we conducted the first systematic review of systematic reviews in the area of international development. As such, we did not find much guidance in the literature on how best to embark on such a review in this particular area of research. Hence, we adopted guidance from the Cochrane Collaboration, which is very focused on health‐related interventions, and we followed advice shared by Polanin et al. (2017) in the context of education research. We acknowledge that there is significant scope to improve methods guidance as well as reporting standards in the context of systematic reviews of reviews in international development.

Following Cochrane guidance and Polanin et al. (2017), we developed a protocol to set out the systematic review of review process (Duvendack & Mader, 2018). We adopted strict inclusion and exclusion criteria to ensure that only relevant systematic review and meta‐analysis evidence was considered for the purpose of this review. Hence, the search terms were also carefully considered and trialled on numerous occasions. The search process was very comprehensive, placing no restrictions on the language of papers, but was limited to 2010 onwards (as justified in the “Search methods for identification of studies” section). With one exception (a study which was subsequently screened out), we only identified English language studies, which may suggest that non‐English language studies may not be picked up sufficiently in the course of the search process; this may apply especially to contexts where non‐Latin alphabets are used, for example, Hindi and Chinese scripts were not picked up.

The rigidity of the quality appraisal process that we followed, as per our protocol, led us to exclude from in‐depth analysis a number of meta‐studies that, using slightly different sets of criteria (and acknowledging the subjectivity and margins in applying some of these criteria, as discussed on various occasions above, and as previously raised by Shea et al., 2017), might have merited inclusion. The appraisal process we adopted may have reduced the risk of bias for our systematic review of reviews, but gave us a more limited evidence base to work with than we would have ideally hoped for.

The two lead reviewers have extensively published in the financial inclusion context, their potential conflicts of interest are clearly acknowledged in the relevant section (below).

### 7.5 Agreements and disagreements with other studies or reviews

No conflict with other systematic reviews of reviews exist, as this is the first one on this topic.

## 8 AUTHORS’ CONCLUSIONS

### 8.1 Implications for practice and policy

We recognise, to follow Whitty (2015, p. 3), that “[p]olicymaking is a professional skill [and] most scientists have no experience of it and it shows”.^34^ Consequently, we aim for no grand conclusions, but rather would let the results summary largely speak for itself. We hope to have reduced the amount of confusion and uncertainty arising from the many different meta‐studies on financial inclusion published in recent years, not least thanks to our systematic assessment of the variations in quality within that field.

We note that, fortunately, our findings regarding impact chime in with an emerging realism around microfinance, including in the donor community: recognising that erstwhile claims of transformative impact were unrealistic and that the hype for microfinance, particularly microcredit, was overblown. We welcome this newfound realism and wish to encourage it with the help of this review, in which we provided a systematic review of the evidence as well as the areas of doubt in the evidence base. At the same time, we wished that going through all stages of the hype cycle—enthusiasm, inflated expectations, and disillusionment—had not been necessary in order to arrive here. And lastly, we must warn that we see a similar hype of strong claims emerging around the much more encompassing notion of financial inclusion, with the promise of marrying macro‐structural economic improvements with micro‐structural poverty relief. Consequently, we chose “financial inclusion” rather than “microfinance” as the frame of this evidence review. We found no evidence for the wider claims made for the beneficence of financial inclusion, as offering poor people a better service, or as having broader macro‐structural effects, being any truer than those once made for microfinance, in large part due to a lack of appropriate research at the meta‐study level. We strongly caution against repeating the hype cycle, this time around the idea of financial inclusion.

A rigorous assessment of the meta‐study evidence base on financial inclusion impacts led us to find impacts that often varied, and that were often more likely to be positive than negative but that also largely occurred in the early stages of the causal chain, which casts doubts onto their being transformative in nature, scope and/or scale. The impacts we found further along the causal chain, on indicators such as incomes or assets, were very small and not consistent across study samples and programmatic contexts or types of interventions. The effects of financial services on women's empowerment seem to be an exception, with generally positive impacts, but again, these impacts are small, often are related to non‐financial programme features, and are highly dependent on the aspects of empowerment under investigation. Also, they were often shaped by diverging views and methodologies regarding how to assess empowerment. Furthermore, quality concerns at the meta‐study as well as at the primary study level should further caution against reading too much into the findings of impacts that we report; major doubts remain about the confidence we can place in many meta‐study findings. In particular, the lack of consistent findings regarding enterprise growth and entrepreneurship propensity lends some credence to the turn away from focusing on microenterprise promotion in financial inclusion. One promising aspect we discovered, however, relates to accessing savings opportunities. The effects may be small, but they are more consistently positive than some of the other effects we found, with fewer downside risks for the users than from credit products. Savings does some good and little harm.

### 8.2 Implications for research

We have taken the evolution of the financial inclusion impact literature toward a natural conclusion, with a higher level of evidence systematisation, to provide an overview of what has become an increasingly perplexing array of meta‐studies that each offer partial overviews. By reviewing these reviews, we have drawn on what is likely the largest‐ever evidence base on financial inclusion impacts, and have uncovered strengths, gaps and weaknesses of the existing high‐level evidence.

From this review, the (perhaps boring) truth that seems to emerge about financial inclusion is that it is not changing the world. On average, financial services may not even have a meaningful net positive effect on poor or low‐income users, although some services have some positive effects for some people. Considering that for most people financial inclusion (which financial services they can access, and how they use them) will be only one among many possible determinants of their life chances and their socio‐economic well‐being, this finding ought not to be unexpected, and we anticipate that it will be confirmed by future research. Our findings add to an emerging realism about microfinance, which we hope will soon extend to the presently more inflated expectations for financial inclusion.

In terms of evidence gaps, it is noteworthy that none of the meta‐studies we reviewed (high‐, medium‐ or low‐confidence) managed to assess debt levels or indebtedness patterns in depth as an outcome of financial inclusion. While we cannot comment on the precise reasons for the lack of attention paid to the issue, we are aware of it being a blind spot of the underlying primary studies. We find this to be a glaring omission of the financial inclusion literature as a whole, and argue the political economy of research funding needs to shift such that researchers are enabled and encouraged to more rigorously explore the most important downsides and risks of development initiatives like financial inclusion. Furthermore, we found no evidence (among the high‐, medium‐ or low‐confidence meta‐studies) for the claim that financial inclusion interventions lead to macroeconomic development and subsequent improvements in the lives of the poor. This may be because the argument has only become prominent in recent years. There is also not much attention given (among the high‐, medium‐ or low‐confidence meta‐studies) to service/amenities‐related programmes such as water credit, sanitation loans, or loans for micro solar systems, especially the notion of “Green Microfinance” where microfinance is applied to promote environmental sustainability.

Given that the majority of financial inclusion impacts we found in assessing the high‐ and medium‐confidence studies were at the early stages of the causal chain, there is a need for studies to better capture long‐term effects and demonstrate more meaningful impacts, especially at the final stages of the causal chain. The vast majority of the studies that our meta‐studies had reviewed had a duration of 1–3 years. These studies are likelier to find changes in behaviours or attitudes rather than structural changes to people's poverty status, and it is not safe to assume that the latter will result from the former. The design of most studies underlying the meta‐studies that we reviewed has not been conducive to establishing whether short‐term or immediate outcomes (such as financial knowledge or entrepreneurial propensity) would translate into intermediate outcomes (such as savings accumulation or microenterprise income) and especially more distal, transformative outcomes (higher net worth or higher incomes). We would suggest that this also reflects a problem of the political economy of development research: a combination of funder restrictions (favouring shorter timelines over multi‐year projects) and a difficulty of gaining long‐term support from the implementer organisations.

Finally, we only found meta‐study (Peters et al., 2016) exclusively drawing on qualitative studies, and two that systematically incorporated qualitative evidence for part of their review (Brody et al., 2015; Vaessen et al., 2014), which suggests that qualitative evidence is still under‐utilised in the systematic review process.

We have also encountered some important limitations of working at this level of systematisation, including: difficulties of assessing the reliability of the levels of evidence underlying ours (respectively having to rely on others’ claims and assessments of its reliability and having to deal with a lack of reporting); analysing effect sizes that are presented in standardised and indexed form, which often reveal little about the underlying measures used (which can be contested and highly heterogeneous, as in the case of women's empowerment—see Vaessen et al., 2014 for a good discussion); the different ways in which data have been analysed and findings presented across very different types of meta‐studies; crude categories for intervention and outcome types, lumping together a highly diverse evidence base that muddies the waters further.

Going forward, we would recommend that authors of primary studies and meta‐studies engage more critically with study quality and ensure better, more detailed reporting of the concepts, data and methods they used. At the review of review level, more methods guidance (especially in terms of synthesis approaches) and clearer reporting standards that adapt the Cochrane health‐focused guidance to the social science and international development context would be helpful.

### 8.3 ROLES AND RESPONSIBILITIES

- Content: Maren Duvendack, Philip Mader
- Systematic review methods: Maren Duvendack, John Eyers (search strategy)
- Statistical analysis: Maren Duvendack, Philip Mader, Daniela Anda Leon
- Information retrieval: John Eyers, Ada Sonnenfeld
- Initial inclusion screening: Ada Sonnenfeld and Raj Popat
- Data extraction: Ada Sonnenfeld, Daniela Anda Leon, Esther Winslow
- Overlap matrices: Miriam Berretta

## 9 SOURCES OF SUPPORT

The International Initiative for Impact Evaluation (3ie) is funding this work.

## 10 DECLARATIONS OF INTEREST

Maren Duvendack was lead author on one systematic review (Duvendack et al., 2011) and contributing author on one (Vaessen et al., 2014).

Philip Mader conducted an overview of (only most recent) financial inclusion impact evidence in early 2017 for a consultancy (unpublished).

Neither author has any professional affiliation with or received research funding from organisations engaged in financial inclusion activities.

## 11 PLANS FOR UPDATING THE REVIEW

Ideally, this review should be updated every five years to include new systematic reviews and meta‐analyses on financial inclusion. However, regular updates are subject to availability of funding.

## Supporting information

Supporting information

## Footnotes

1 We use the term primary studies to denote individual studies that make up a systematic review and meta‐analysis.

2
https://sustainabledevelopment.un.org/sdg1

3
https://sustainabledevelopment.un.org/sdg5

4
https://www.worldbank.org/en/topic/financialinclusion/overview, as of September 28, 2018.

5 Financial inclusion also aims at service provision for marginal populations in higher‐income countries, but this lies outside of the remit of this review.

6 We use the term systematic review of reviews to denote our approach; we refer to population of studies consisting of systematic reviews and meta‐analyses collectively as meta‐studies, or often just as “reviews”.

7 This reflects the distinction between intervention‐types in the medium‐ and high‐confidence meta‐studies that we included in our systematic review of reviews (see Section 6, below). None of these meta‐studies focused on money transfers or digital financial services.

8 This draws partly on the representation of enablers of financial inclusion in the World Bank's Universal Financial Access 2020 initiative (http://www.worldbank.org/en/topic/financialinclusion/brief/achieving‐universal‐financial‐access‐by‐2020).

9 To say that “women's visibility and mobility” or “women's solidarity and mutual support” are outcomes of a lower order than, for instance, “women's income independence”, would involve a value judgment that we do not intend to make. In Figure 1, they are presented in different levels only to suggest one possible pathway of causation. In the discussion of results, we refrain from any distinction between lower‐ and higher‐order gender outcomes.

10 Most prominently in the *World Development Report 2015: Mind, Society, and Behaviour*.

11 Evans and Popova (2015) produced a review that claimed to find divergent findings in six “systematic reviews” of education programmes. However, the authors did not screen or critically appraise included reviews according to standard definitions. Hence, further analysis indicated only one of the included studies was undertaken using systematic review methods, the other five being literature reviews and meta‐analyses which did not use comprehensive approaches to select, appraise and/or synthesise the evidence (Snilstveit, Vojtkova, & Phillips, 2015).

12 Our review disaggregates the impacts of different services provided by different provider‐types, and examine heterogeneous impacts on different user groups, as much as the data permits.

13 We found no meta‐studies that addressed this type of impact, but hope to see such evidence in the future.

14 In the systematic review context, PICOS—standing for Population‐Intervention‐Comparison‐Outcome‐Study design—are used to determine eligibility for inclusion or exclusion of studies. We adapted these criteria for the systematic review of review process as follows: type of review (S—to capture systematic reviews and meta‐analyses), types of participants (P), types of interventions (I) and types of outcome measures (O), comparison (C) is not applicable.

15 The World Bank definition of lower/middle‐income countries is used: https://datahelpdesk.worldbank.org/knowledgebase/articles/906519‐world‐bank‐country‐and‐lending‐groups

16
http://www.kobotoolbox.org/

17
http://www.3ieimpact.org/media/filer_public/2012/05/07/quality_appraisal_checklist_srdatabase.pdf

18
https://www.cardiff.ac.uk/__data/assets/pdf_file/0005/212756/SURE_SR‐Checklist_2015.pdf

19 See online checklist for details: https://amstar.ca/Amstar_Checklist.php

20 A good overview of all GRADE related papers can be found here: http://bestpractice.bmj.com/info/toolkit/learn‐ebm/what‐is‐grade/

21 We explored the possibility of conducting a network meta‐analysis (Ioannidis, 2009) to synthesise the reviews that performed meta‐analysis but our sample of meta‐analyses was too small and the outcome variables and effect size calculations used in each individual meta‐analysis too heterogeneous to allow a meaningful network meta‐analysis.

22
http://methods.cochrane.org/sites/methods.cochrane.org.equity/files/public/uploads/EquityChecklist2012.pdf, see also O'Neill et al., (2014).

23 As discussed above, PICOS stands for Population‐Intervention‐Comparison‐Outcome‐Study design and determines eligibility for inclusion or exclusion of studies. We adapted these criteria to suit our purposes.

24 See online checklist for details: https://amstar.ca/Amstar_Checklist.php

25
http://www.3ieimpact.org/media/filer_public/2012/05/07/quality_appraisal_checklist_srdatabase.pdf

26 No meta‐study evidence relating to macro‐structural effects of financial inclusion was found.

27 We did not extract data on the total number of participants (i.e., households or individuals participating in financial inclusion interventions) as this information was not provided across all meta‐studies. Rather than presenting an incomplete picture on participants, we chose not to report this information as peripheral to any review of meta‐studies. To obtain this information, it would have been necessary to check each primary study included in each of the meta‐studies.

28 Gopalaswamy et al. (2016) exemplifies this tendency.

29 Steinert et al. (2018) and Vaessen et al. (2014) are fine examples.

30 Rossi (1987), cited by David Roodman (2009): https://www.cgdev.org/blog/rossis‐rules

31 We used an adapted PROGRESS checklist (O'Neill et al., 2014) to identify these factors that seem to drive heterogeneity in the financial inclusion context. However, it was very difficult to further unpack these drivers of heterogeneity as the reviews we included did not provide further disaggregated information, they rather created broad categories lumping together a range of diverse outcomes and intervention types as a way to deal with high levels of heterogeneity.

32 These contain four studies of insurance, two of which we were surprised to have had to exclude following our formal quality assessment criteria.

33 We were surprised that the 3ie critical appraisal checklist and AMSTAR2 tools led us to exclude some of these insurance meta‐studies, whose quality we would subjectively have assessed to be higher.

34 Christopher Whitty was Chief Scientific Adviser at the UK Department for International Development (DFID).
