# Supplementary material for: Impact of financial inclusion in low‐ and middle‐income countries: A systematic review of reviews
Source: Campbell Syst Rev. 2019 Jul 23;15(1-2):e1012. doi: 10.4073/csr.2019.2 (PMC8356488; doi:10.4073/csr.2019.2)
Supplement: Supplementary file 1 — Supporting information [file CL2-15-e1012-s001.docx]

# Appendices

1. Appendix 1 – Search strategies
2. Appendix 2 – MECIR checklist
3. Appendix 3 – List of included meta-studies and their main research question
4. Appendix 4 – Number and proportion of meta-studies by PICOS characteristics
5. Appendix 5 – List of excluded studies
6. Appendix 6 – Correlation matrix of low confidence meta-studies to demonstrate overlap
7. Appendix 7 – AMSTAR2 and 3ie critical appraisal checklist
8. Appendix 8 – Quality assessment of included meta-studies, low confidence
9. Appendix 9 – Overview of quality assessment criteria for low-confidence meta-studies

### Appendix 1 - Search strategies

1. **Academic Search Complete (EBSCO) – Searched 10^th^ November 2017**

S23 S11 AND S16 AND S21 Limiters - Published Date: 20100101-20181231

**366 hits**

S22 S16 AND S21

2,637

S21 S17 OR S18 OR S19 OR S20

216,396

S20 TI ( ( mhealth or "mobile health" or m-health ) ) OR AB ( ( mhealth or "mobile health" or m-health ) ) OR SU ( ( mhealth or "mobile health" or m-health ) )

1,504

S19 SU (micro-finance OR "micro finance" OR microfinance OR micro-loan* OR microloan* OR "micro loan*" OR microleas* OR micro-leas* OR "micro leas*" OR microlending OR micro-lending OR "micro lending" OR microinsurance OR micro-insurance OR "micro insurance" OR "microgroup lending" OR microfranchis* OR micro-franchis* OR "micro franchis*" OR "micro credit*" OR microcredit* OR micro-credit* OR "micro enterprise*" OR microenterprise* OR micro-enterprise* OR "micro entrepreneur*" OR microentrepreneur* OR micro-entrepreneur* OR saving* OR micro-saving* OR microsaving* OR "Smallholder financ*" OR "rural financ*" OR "rural credit" OR ROSCAs OR SHGs OR "group lending" OR "community savings" or "small loan*" or "small lend*" or ((bank or credit*) N3 cooperat*) or ((credit or loan* or lend*) N3 program*) or (community N3 (bank* or saving* or loan* or lend*)) or "income generat*" or grameen OR ROSCA* OR stokvel* OR ((financial OR economic) N2 (literacy OR education OR skills OR training OR knowledge OR capab*)) OR banking OR budgeting OR "money manag*" OR "consumption smoothing" OR rationing OR earmarking OR "bank account*" OR "youth account*" OR "lock box*" OR "piggy bank*" OR "saving box*" OR ((access* OR participat*) N3 ( financ* OR credit OR saving* OR loan* OR lending)) OR "financial inclusion" OR "inclusive finance" OR fintech OR "mobile monies" OR M-PESA OR "mobile banking" OR cashless )

66,273

S18 AB (micro-finance OR "micro finance" OR microfinance OR micro-loan* OR microloan* OR "micro loan*" OR microleas* OR micro-leas* OR "micro leas*" OR microlending OR micro-lending OR "micro lending" OR microinsurance OR micro-insurance OR "micro insurance" OR "microgroup lending" OR microfranchis* OR micro-franchis* OR "micro franchis*" OR "micro credit*" OR microcredit* OR micro-credit* OR "micro enterprise*" OR microenterprise* OR micro-enterprise* OR "micro entrepreneur*" OR microentrepreneur* OR micro-entrepreneur* OR saving* OR micro-saving* OR microsaving* OR "Smallholder financ*" OR "rural financ*" OR "rural credit" OR SHGs OR "group lending" OR "community savings" or "small loan*" or "small lend*" or ((bank or credit*) N3 cooperat*) or ((credit or loan* or lend*) N3 program*) or (community N3 (bank* or saving* or loan* or lend*)) or "income generat*" or grameen OR ROSCA* OR stokvel* OR ((financial OR economic) N2 (literacy OR education OR skills OR training OR knowledge OR capab*)) OR banking OR budgeting OR "money manag*" OR "consumption smoothing" OR rationing OR earmarking OR "bank account*" OR "youth account*" OR "lock box*" OR "piggy bank*" OR "saving box*" OR ((access* OR participat*) N3 ( financ* OR credit OR saving* OR loan* OR lending)) OR "financial inclusion" OR "inclusive finance" OR fintech OR "mobile monies" OR M-PESA OR "mobile banking" OR cashless)

158,815

S17 TI (micro-finance OR "micro finance" OR microfinance OR micro-loan* OR microloan* OR "micro loan*" OR microleas* OR micro-leas* OR "micro leas*" OR microlending OR micro-lending OR "micro lending" OR microinsurance OR micro-insurance OR "micro insurance" OR "microgroup lending" OR microfranchis* OR micro-franchis* OR "micro franchis*" OR "micro credit*" OR microcredit* OR micro-credit* OR "micro enterprise*" OR microenterprise* OR micro-enterprise* OR "micro entrepreneur*" OR microentrepreneur* OR micro-entrepreneur* OR saving* OR micro-saving* OR microsaving* OR "Smallholder financ*" OR "rural financ*" OR "rural credit" OR SHGs OR "group lending" OR "community savings" or "small loan*" or "small lend*" or ((bank or credit*) N3 cooperat*) or ((credit or loan* or lend*) N3 program*) or (community N3 (bank* or saving* or loan* or lend*)) or "income generat*" or grameen OR ROSCA* OR stokvel* OR ((financial OR economic) N2 (literacy OR education OR skills OR training OR knowledge OR capab*)) OR banking OR budgeting OR "money manag*" OR "consumption smoothing" OR rationing OR earmarking OR "bank account*" OR "youth account*" OR "lock box*" OR "piggy bank*" OR "saving box*" OR ((access* OR participat*) N3 ( financ* OR credit OR saving* OR loan* OR lending)) OR "financial inclusion" OR "inclusive finance" OR fintech OR "mobile monies" OR M-PESA OR "mobile banking" OR cashless )

34,309

S16 S12 or S13 or S14 or S15

433,532

S15 TI ( ("literature search" OR "database search" OR "bibliographic* search" OR "comprehensive search" OR "extensive search" OR "exhaustive search" OR "purposive search" OR "representative search" or "systemat* search") ) OR AB ( ("literature search" OR "database search" OR "bibliographic* search" OR "comprehensive search" OR "extensive search" OR "exhaustive search" OR "purposive search" OR "representative search" or "systemat* search") ) OR SU ( ("literature search" OR "database search" OR "bibliographic* search" OR "comprehensive search" OR "extensive search" OR "exhaustive search" OR "purposive search" OR "representative search" or "systemat* search") )

25,968

S14 TI ( (review N3 (effectiveness OR effects OR systemat* OR synth* OR integrat* OR map* OR methodologic* OR quantitative OR evidence OR literature)) ) OR AB ( (review N3 (effectiveness OR effects OR systemat* OR synth* OR integrat* OR map* OR methodologic* OR quantitative OR evidence OR literature)) ) OR SU ( (review N3 (effectiveness OR effects OR systemat* OR synth* OR integrat* OR map* OR methodologic* OR quantitative OR evidence OR literature)) )

223,448

S13 TI ( ("Meta regression" OR "meta synth*" OR "meta-synth*" OR "meta analy*" OR "metaanaly*" OR "meta-analy*" OR "metanaly*" OR "Metaregression" OR "Meta-regression" OR "Methodologic* overview" OR "pool* analys*" OR "pool* data" OR "Quantitative* overview" OR "research integration") ) OR AB ( ("Meta regression" OR "meta synth*" OR "meta-synth*" OR "meta analy*" OR "metaanaly*" OR "meta-analy*" OR "metanaly*" OR "Metaregression" OR "Meta-regression" OR "Methodologic* overview" OR "pool* analys*" OR "pool* data" OR "Quantitative* overview" OR "research integration") ) OR SU ( ("Meta regression" OR "meta synth*" OR "meta-synth*" OR "meta analy*" OR "metaanaly*" OR "meta-analy*" OR "metanaly*" OR "Metaregression" OR "Meta-regression" OR "Methodologic* overview" OR "pool* analys*" OR "pool* data" OR "Quantitative* overview" OR "research integration") )

81,114

S12 TI ( ((Systematic* OR synthes*) N3 (Research OR evaluation* OR finding* OR thematic* OR report OR descriptive OR explanatory OR narrative OR meta* OR review* OR data OR literature OR studies OR evidence OR map OR quantitative OR study OR studies OR paper OR impact OR impacts OR effect* OR compar*)) ) OR AB ( ((Systematic* OR synthes*) N3 (Research OR evaluation* OR finding* OR thematic* OR report OR descriptive OR explanatory OR narrative OR meta* OR review* OR data OR literature OR studies OR evidence OR map OR quantitative OR study OR studies OR paper OR impact OR impacts OR effect* OR compar*)) ) OR SU ( ((Systematic* OR synthes*) N3 (Research OR evaluation* OR finding* OR thematic* OR report OR descriptive OR explanatory OR narrative OR meta* OR review* OR data OR literature OR studies OR evidence OR map OR quantitative OR study OR studies OR paper OR impact OR impacts OR effect* OR compar*)) )

238,573

S11 S1 OR S2 OR S3 OR S4 OR S5 OR S6 OR S7 OR S8 OR S9 OR S10

2,444,336

S10 TI (Africa or Asia or Caribbean or "West Indies" or "South America" or "Latin America" or "Central America" OR "Middle East") OR AB (Africa or Asia or Caribbean or "West Indies" or "South America" or "Latin America" or "Central America" OR "Middle East") OR SU (Africa or Asia or Caribbean or "West Indies" or "South America" or "Latin America" or "Central America" OR "Middle East") OR GE (Africa or Asia or Caribbean or "West Indies" or "South America" or "Latin America" or "Central America" OR "Middle East")

453,330

S9 TI ( ("transitional country" or "transitional countries") ) OR AB ( ("transitional country" or "transitional countries") ) OR SU ( ("transitional country" or "transitional countries") )

227

S8 TI ( (lmic or lmics or "third world" or "lami country" or "lami countries") ) OR AB ( (lmic or lmics or "third world" or "lami country" or "lami countries") ) OR SU ( (lmic or lmics or "third world" or "lami country" or "lami countries") )

10,222

S7 TI (low N3 middle N3 countr*) OR AB (low N3 middle N3 countr*) OR SU (low N3 middle N3 countr*)

6,211

S6 TI ( low* N1 (gdp or gnp or "gross domestic" or "gross national") ) OR AB ( low* N1 (gdp or gnp or "gross domestic" or "gross national") ) OR SU ( low* N1 (gdp or gnp or "gross domestic" or "gross national") )

256

S5 TI ( (developing or less* N1 developed or "under developed" or underdeveloped or "middle income" or low* N1 income) N1 (economy or economies) ) OR AB ( (developing or less* N1 developed or "under developed" or underdeveloped or "middle income" or low* N1 income) N1 (economy or economies) ) OR SU ( (developing or less* N1 developed or "under developed" or underdeveloped or "middle income" or low* N1 income) N1 (economy or economies) )

1,996

S4 TI ( (developing or less* N1 developed or "under developed" or underdeveloped or "middle income" or low* N1 income or underserved or "under served" or deprived or poor*) N1 (countr* or nation* or population* or world) ) OR AB ( (developing or less* N1 developed or "under developed" or underdeveloped or "middle income" or low* N1 income or underserved or "under served" or deprived or poor*) N1 (countr* or nation* or population* or world) ) OR SU ( (developing or less* N1 developed or "under developed" or underdeveloped or "middle income" or low* N1 income or underserved or "under served" or deprived or poor*) N1 (countr* or nation* or population* or world) )

94,574

S3 AB Afghanistan OR Albania OR Algeria OR Angola OR Antigua OR Barbuda OR Argentina OR Armenia OR Armenian OR Aruba OR Azerbaijan OR Bahrain OR Bangladesh OR Barbados OR Benin OR Belize OR Bhutan OR Bolivia OR Botswana OR Brazil OR Brasil OR "Burkina Faso" OR "Burkina Fasso" OR "Upper Volta" OR Burundi OR Urundi OR Cambodia OR "Khmer Republic" OR Kampuchea OR Cameroon OR Cameroons OR Cameron OR Camerons OR "Cape Verde" OR "Central African Republic" OR Chad OR Chile OR China OR Colombia OR Comoros OR "Comoro Islands" OR Comores OR Mayotte OR Congo OR Zaire OR "Costa Rica" OR "Cote d'Ivoire" OR "Ivory Coast" OR Cuba OR "Djibouti" OR "French Somaliland" OR Dominica OR "Dominican Republic" OR "East Timor" OR "East Timur" OR "Timor Leste" OR Ecuador OR Egypt OR "United Arab Republic" OR "El Salvador" OR Eritrea OR Ethiopia OR Fiji OR Gabon OR "Gabonese Republic" OR Gambia OR Gaza OR "Georgia Republic" OR "Georgian Republic" OR Ghana OR "Gold Coast" OR Grenada OR Guatemala OR Guinea OR Guam OR Guiana OR Guyana OR Haiti OR Honduras OR India OR Maldives OR Indonesia OR Iran OR Iraq OR Jamaica OR Jordan OR Kazakhstan OR Kazakh OR Kenya OR Kiribati OR Korea OR Kosovo OR Kyrgyzstan OR Kirghizia OR "Kyrgyz Republic" OR Kirghiz OR Kirgizstan OR "Lao PDR" OR Laos OR Lebanon OR Lesotho OR Basutoland OR Liberia OR Libya OR Madagascar OR "Malagasy Republic" OR Malaysia OR Malaya OR Malay OR Sabah OR Sarawak OR Malawi OR Nyasaland OR Mali OR "Marshall Islands" OR Mauritania OR Mauritius OR "Agalega Islands" OR Mexico OR Micronesia OR "Middle East" OR Moldova OR Moldovia OR Moldovian OR Mongolia OR Montenegro OR Morocco OR Ifni OR Mozambique OR Myanmar OR Myanma OR Burma OR Namibia OR Nepal OR Antilles OR "New Caledonia" OR Nicaragua OR Niger OR Nigeria OR "Mariana Islands" OR Oman OR Muscat OR Pakistan OR Palau OR Palestine OR Panama OR Paraguay OR Peru OR Philippines OR Philipines OR Phillipines OR Phillippines OR "Puerto Rico" OR Rwanda OR Ruanda OR "Saint Kitts" OR "St Kitts" OR Nevis OR "Saint Lucia" OR "St Lucia" OR "Saint Vincent" OR "St Vincent" OR "Grenadines" OR "Samoa" OR "Samoan Islands" OR "Navigator Island" OR "Navigator Islands" OR "Sao Tome" OR "Saudi Arabia" OR Senegal OR Seychelles OR "Sierra Leone" OR "Sri Lanka" OR "Solomon Islands" OR Somalia OR Sudan OR Suriname OR Surinam OR Swaziland OR Syria OR Tajikistan OR Tadzhikistan OR Tadjikistan OR Tadzhik OR Tanzania OR Thailand OR Togo OR "Togolese Republic" OR Tonga OR Trinidad OR Tobago OR Tunisia OR Turkey OR Turkmenistan OR Turkmen OR Uganda OR Ukraine OR Uruguay OR Uzbekistan OR Uzbek OR Vanuatu OR "New Hebrides" OR Venezuela OR Vietnam OR "Viet Nam" OR "West Bank" OR Yemen OR Zambia OR Zimbabwe OR Jamahiriya OR Jamahiryria OR Libia OR Mocambique OR Principe OR Syrian OR "Indian Ocean" OR Melanesia OR "Western Sahara"

1,733,865

S2 TI Afghanistan OR Albania OR Algeria OR Angola OR Antigua OR Barbuda OR Argentina OR Armenia OR Armenian OR Aruba OR Azerbaijan OR Bahrain OR Bangladesh OR Barbados OR Benin OR Belize OR Bhutan OR Bolivia OR Botswana OR Brazil OR Brasil OR "Burkina Faso" OR "Burkina Fasso" OR "Upper Volta" OR Burundi OR Urundi OR Cambodia OR "Khmer Republic" OR Kampuchea OR Cameroon OR Cameroons OR Cameron OR Camerons OR "Cape Verde" OR "Central African Republic" OR Chad OR Chile OR China OR Colombia OR Comoros OR "Comoro Islands" OR Comores OR Mayotte OR Congo OR Zaire OR "Costa Rica" OR "Cote d'Ivoire" OR "Ivory Coast" OR Cuba OR "Djibouti" OR "French Somaliland" OR Dominica OR "Dominican Republic" OR "East Timor" OR "East Timur" OR "Timor Leste" OR Ecuador OR Egypt OR "United Arab Republic" OR "El Salvador" OR Eritrea OR Ethiopia OR Fiji OR Gabon OR "Gabonese Republic" OR Gambia OR Gaza OR "Georgia Republic" OR "Georgian Republic" OR Ghana OR "Gold Coast" OR Grenada OR Guatemala OR Guinea OR Guam OR Guiana OR Guyana OR Haiti OR Honduras OR India OR Maldives OR Indonesia OR Iran OR Iraq OR Jamaica OR Jordan OR Kazakhstan OR Kazakh OR Kenya OR Kiribati OR Korea OR Kosovo OR Kyrgyzstan OR Kirghizia OR "Kyrgyz Republic" OR Kirghiz OR Kirgizstan OR "Lao PDR" OR Laos OR Lebanon OR Lesotho OR Basutoland OR Liberia OR Libya OR Madagascar OR "Malagasy Republic" OR Malaysia OR Malaya OR Malay OR Sabah OR Sarawak OR Malawi OR Nyasaland OR Mali OR "Marshall Islands" OR Mauritania OR Mauritius OR "Agalega Islands" OR Mexico OR Micronesia OR "Middle East" OR Moldova OR Moldovia OR Moldovian OR Mongolia OR Montenegro OR Morocco OR Ifni OR Mozambique OR Myanmar OR Myanma OR Burma OR Namibia OR Nepal OR Antilles OR "New Caledonia" OR Nicaragua OR Niger OR Nigeria OR "Mariana Islands" OR Oman OR Muscat OR Pakistan OR Palau OR Palestine OR Panama OR Paraguay OR Peru OR Philippines OR Philipines OR Phillipines OR Phillippines OR "Puerto Rico" OR Rwanda OR Ruanda OR "Saint Kitts" OR "St Kitts" OR Nevis OR "Saint Lucia" OR "St Lucia" OR "Saint Vincent" OR "St Vincent" OR "Grenadines" OR "Samoa" OR "Samoan Islands" OR "Navigator Island" OR "Navigator Islands" OR "Sao Tome" OR "Saudi Arabia" OR Senegal OR Seychelles OR "Sierra Leone" OR "Sri Lanka" OR "Solomon Islands" OR Somalia OR Sudan OR Suriname OR Surinam OR Swaziland OR Syria OR Tajikistan OR Tadzhikistan OR Tadjikistan OR Tadzhik OR Tanzania OR Thailand OR Togo OR "Togolese Republic" OR Tonga OR Trinidad OR Tobago OR Tunisia OR Turkey OR Turkmenistan OR Turkmen OR Uganda OR Ukraine OR Uruguay OR Uzbekistan OR Uzbek OR Vanuatu OR "New Hebrides" OR Venezuela OR Vietnam OR "Viet Nam" OR "West Bank" OR Yemen OR Zambia OR Zimbabwe OR Jamahiriya OR Jamahiryria OR Libia OR Mocambique OR Principe OR Syrian OR "Indian Ocean" OR Melanesia OR "Western Sahara"

837,520

S1 SU Afghanistan OR Albania OR Algeria OR Angola OR Antigua OR Barbuda OR Argentina OR Armenia OR Armenian OR Aruba OR Azerbaijan OR Bahrain OR Bangladesh OR Barbados OR Benin OR Belize OR Bhutan OR Bolivia OR Botswana OR Brazil OR Brasil OR "Burkina Faso" OR "Burkina Fasso" OR "Upper Volta" OR Burundi OR Urundi OR Cambodia OR "Khmer Republic" OR Kampuchea OR Cameroon OR Cameroons OR Cameron OR Camerons OR "Cape Verde" OR "Central African Republic" OR Chad OR Chile OR China OR Colombia OR Comoros OR "Comoro Islands" OR Comores OR Mayotte OR Congo OR Zaire OR "Costa Rica" OR "Cote d'Ivoire" OR "Ivory Coast" OR Cuba OR "Djibouti" OR "French Somaliland" OR Dominica OR "Dominican Republic" OR "East Timor" OR "East Timur" OR "Timor Leste" OR Ecuador OR Egypt OR "United Arab Republic" OR "El Salvador" OR Eritrea OR Ethiopia OR Fiji OR Gabon OR "Gabonese Republic" OR Gambia OR Gaza OR "Georgia Republic" OR "Georgian Republic" OR Ghana OR "Gold Coast" OR Grenada OR Guatemala OR Guinea OR Guam OR Guiana OR Guyana OR Haiti OR Honduras OR India OR Maldives OR Indonesia OR Iran OR Iraq OR Jamaica OR Jordan OR Kazakhstan OR Kazakh OR Kenya OR Kiribati OR Korea OR Kosovo OR Kyrgyzstan OR Kirghizia OR "Kyrgyz Republic" OR Kirghiz OR Kirgizstan OR "Lao PDR" OR Laos OR Lebanon OR Lesotho OR Basutoland OR Liberia OR Libya OR Madagascar OR "Malagasy Republic" OR Malaysia OR Malaya OR Malay OR Sabah OR Sarawak OR Malawi OR Nyasaland OR Mali OR "Marshall Islands" OR Mauritania OR Mauritius OR "Agalega Islands" OR Mexico OR Micronesia OR "Middle East" OR Moldova OR Moldovia OR Moldovian OR Mongolia OR Montenegro OR Morocco OR Ifni OR Mozambique OR Myanmar OR Myanma OR Burma OR Namibia OR Nepal OR Antilles OR "New Caledonia" OR Nicaragua OR Niger OR Nigeria OR "Mariana Islands" OR Oman OR Muscat OR Pakistan OR Palau OR Palestine OR Panama OR Paraguay OR Peru OR Philippines OR Philipines OR Phillipines OR Phillippines OR "Puerto Rico" OR Rwanda OR Ruanda OR "Saint Kitts" OR "St Kitts" OR Nevis OR "Saint Lucia" OR "St Lucia" OR "Saint Vincent" OR "St Vincent" OR "Grenadines" OR "Samoa" OR "Samoan Islands" OR "Navigator Island" OR "Navigator Islands" OR "Sao Tome" OR "Saudi Arabia" OR Senegal OR Seychelles OR "Sierra Leone" OR "Sri Lanka" OR "Solomon Islands" OR Somalia OR Sudan OR Suriname OR Surinam OR Swaziland OR Syria OR Tajikistan OR Tadzhikistan OR Tadjikistan OR Tadzhik OR Tanzania OR Thailand OR Togo OR "Togolese Republic" OR Tonga OR Trinidad OR Tobago OR Tunisia OR Turkey OR Turkmenistan OR Turkmen OR Uganda OR Ukraine OR Uruguay OR Uzbekistan OR Uzbek OR Vanuatu OR "New Hebrides" OR Venezuela OR Vietnam OR "Viet Nam" OR "West Bank" OR Yemen OR Zambia OR Zimbabwe OR Jamahiriya OR Jamahiryria OR Libia OR Mocambique OR Principe OR Syrian OR "Indian Ocean" OR Melanesia OR "Western Sahara"

1,272,552

**EBSCO Discovery Service - Searched 10^th^ November 2017**

Strategy for Academic Search Complete (above) used – limited to:

1. **EconLit (510 hits)**
2. **RePEc (238 hits)**
3. **World Bank e-Library (40 hits)**

1. **Scopus – Searched 10^th^ November 2017**

( ( ( TITLE-ABS-KEY ( mhealth OR "mobile health" OR "m health" OR m-health ) ) ) OR ( ( TITLE-ABS-KEY ( micro-finance OR "micro finance" OR microfinance OR micro-loan* OR microloan* OR "micro loan*" OR microleas* OR micro-leas* OR "micro leas*" OR microlending OR micro-lending OR "micro lending" OR microinsurance OR micro-insurance OR "micro insurance" OR "microgroup lending" OR microfranchis* OR micro-franchis* OR "micro franchis*" OR "micro credit*" OR microcredit* OR micro-credit* OR "micro enterprise*" OR microenterprise* OR micro-enterprise* OR "micro entrepreneur*" OR microentrepreneur* OR micro-entrepreneur* OR saving* OR micro-saving* OR microsaving* OR "Smallholder financ*" OR "rural financ*" OR "rural credit" OR roscas OR shgs OR "group lending" OR "community savings" OR "small loan*" OR "small lend*" OR ( ( bank* OR credit* ) W/3 cooperat* ) OR ( ( credit OR loan* OR lend* ) W/3 program* ) OR ( community W/3 ( bank* OR saving* OR loan* OR lend* ) ) OR "income generat*" OR grameen OR rosca* OR stokvel* OR ( ( financial OR economic ) W/2 ( literacy OR education OR skills OR training OR knowledge OR capab* ) ) OR banking OR budgeting OR "money manag*" OR "consumption smoothing" OR rationing OR earmarking OR "bank account*" OR "youth account*" OR "lock box*" OR "piggy bank*" OR "saving box*" OR ( ( access* OR participat* ) W/3 ( financ* OR credit OR saving* OR loan* OR lending ) ) OR "financial inclusion" OR "inclusive finance" OR fintech OR "mobile monies" OR m-pesa OR "mobile banking" OR cashless ) ) ) ) AND ( ( ( TITLE-ABS-KEY ( "literature search" OR "database search" OR "bibliographic* search" OR "comprehensive search" OR "extensive search" OR "exhaustive search" OR "purposive search" OR "representative search" OR "systemat* search" ) ) ) OR ( ( TITLE-ABS-KEY ( review W/3 ( effectiveness OR effects OR systemat* OR synth* OR integrat* OR map* OR methodologic* OR quantitative OR evidence OR literature ) ) ) ) OR ( ( TITLE-ABS-KEY ( "Meta regression" OR "meta synth*" OR "meta-synth*" OR "meta analy*" OR "metaanaly*" OR "meta-analy*" OR "metanaly*" OR "Metaregression" OR "Meta-regression" OR "Methodologic* overview" OR "pool* analys*" OR "pool* data" OR "Quantitative* overview" OR "research integration" ) ) ) OR ( ( TITLE-ABS-KEY ( ( systematic* OR synthes* ) W/3 ( research OR evaluation* OR finding* OR thematic* OR report OR descriptive OR explanatory OR narrative OR meta* OR review* OR data OR literature OR studies OR evidence OR map OR quantitative OR study OR studies OR paper OR impact OR impacts OR effect* OR compar* ) ) ) ) ) AND ( ( ( TITLE-ABS-KEY ( low* W/1 ( gdp OR gnp OR "gross domestic" OR "gross national" ) ) ) ) OR ( ( TITLE-ABS-KEY ( ( developing OR less* W/1 developed OR "under developed" OR underdeveloped OR "middle income" OR low* W/1 income ) W/1 ( economy OR economies ) ) ) ) OR ( ( TITLE-ABS-KEY ( ( developing OR ( less* W/1 developed ) OR "under developed" OR underdeveloped OR "middle income" OR ( low* W/1 income ) OR underserved OR "under served" OR deprived OR poor* ) W/1 ( countr* OR nation* OR population* OR world ) ) ) ) OR ( ( TITLE-ABS-KEY ( afghanistan OR albania OR algeria OR angola OR antigua OR barbuda OR argentina OR armenia OR armenian OR aruba OR azerbaijan OR bahrain OR bangladesh OR barbados OR benin OR belize OR bhutan OR bolivia OR botswana OR brazil OR brasil OR "Burkina Faso" OR "Burkina Fasso" OR "Upper Volta" OR burundi OR urundi OR cambodia OR "Khmer Republic" OR kampuchea OR cameroon OR cameroons OR cameron OR camerons OR "Cape Verde" OR "Central African Republic" OR chad OR chile OR china OR colombia OR comoros OR "Comoro Islands" OR comores OR mayotte OR congo OR zaire OR "Costa Rica" OR "Cote d'Ivoire" OR "Ivory Coast" OR cuba OR "Djibouti" OR "French Somaliland" OR dominica OR "Dominican Republic" OR "East Timor" OR "East Timur" OR "Timor Leste" OR ecuador OR egypt OR "United Arab Republic" OR "El Salvador" OR eritrea OR ethiopia OR fiji OR gabon OR "Gabonese Republic" OR gambia OR gaza OR "Georgia Republic" OR "Georgian Republic" OR ghana OR "Gold Coast" OR grenada OR guatemala OR guinea OR guam OR guiana OR guyana OR haiti OR honduras OR india OR maldives OR indonesia OR iran OR iraq OR jamaica OR jordan OR kazakhstan OR kazakh OR kenya OR kiribati OR korea OR kosovo OR kyrgyzstan OR kirghizia OR "Kyrgyz Republic" OR kirghiz OR kirgizstan OR "Lao PDR" OR laos OR lebanon OR lesotho OR basutoland OR liberia OR libya OR madagascar OR "Malagasy Republic" OR malaysia OR malaya OR malay OR sabah OR sarawak OR malawi OR nyasaland OR mali OR "Marshall Islands" OR mauritania OR mauritius OR "Agalega Islands" OR mexico OR micronesia OR "Middle East" OR moldova OR moldovia OR moldovian OR mongolia OR montenegro OR morocco OR ifni OR mozambique OR myanmar OR myanma OR burma OR namibia OR nepal OR antilles OR "New Caledonia" OR nicaragua OR niger OR nigeria OR "Mariana Islands" OR oman OR muscat OR pakistan OR palau OR palestine OR panama OR paraguay OR peru OR philippines OR philipines OR phillipines OR phillippines OR "Puerto Rico" OR rwanda OR ruanda OR "Saint Kitts" OR "St Kitts" OR nevis OR "Saint Lucia" OR "St Lucia" OR "Saint Vincent" OR "St Vincent" OR "Grenadines" OR "Samoa" OR "Samoan Islands" OR "Navigator Island" OR "Navigator Islands" OR "Sao Tome" OR "Saudi Arabia" OR senegal OR seychelles OR "Sierra Leone" OR "Sri Lanka" OR "Solomon Islands" OR somalia OR sudan OR suriname OR surinam OR swaziland OR syria OR tajikistan OR tadzhikistan OR tadjikistan OR tadzhik OR tanzania OR thailand OR togo OR "Togolese Republic" OR tonga OR trinidad OR tobago OR tunisia OR turkey OR turkmenistan OR turkmen OR uganda OR ukraine OR uruguay OR uzbekistan OR uzbek OR vanuatu OR "New Hebrides" OR venezuela OR vietnam OR "Viet Nam" OR "West Bank" OR yemen OR zambia OR zimbabwe OR jamahiriya OR jamahiryria OR libia OR mocambique OR principe OR syrian OR "Indian Ocean" OR melanesia OR "Western Sahara" ) ) ) OR ( ( ( TITLE-ABS-KEY ( africa OR asia OR caribbean OR "West Indies" OR "South America" OR "Latin America" OR "Central America" OR "Middle East" ) ) ) OR ( ( TITLE-ABS-KEY ( "transitional country" OR "transitional countries" ) ) ) OR ( ( TITLE-ABS-KEY ( lmic OR lmics OR "third world" OR "lami country" OR "lami countries" ) ) ) OR ( ( TITLE-ABS-KEY ( low W/3 middle W/3 countr* ) ) ) ) ) AND ( LIMIT-TO ( PUBYEAR , 2018 ) OR LIMIT-TO ( PUBYEAR , 2017 ) OR LIMIT-TO ( PUBYEAR , 2016 ) OR LIMIT-TO ( PUBYEAR , 2015 ) OR LIMIT-TO ( PUBYEAR , 2014 ) OR LIMIT-TO ( PUBYEAR , 2013 ) OR LIMIT-TO ( PUBYEAR , 2012 ) OR LIMIT-TO ( PUBYEAR , 2011 ) OR LIMIT-TO ( PUBYEAR , 2010 ) ) 19 ( ( ( TITLE-ABS-KEY ( mhealth OR "mobile health" OR "m health" OR m-health ) ) ) OR ( ( TITLE-ABS-KEY ( micro-finance OR "micro finance" OR microfinance OR micro-loan* OR microloan* OR "micro loan*" OR microleas* OR micro-leas* OR "micro leas*" OR microlending OR micro-lending OR "micro lending" OR microinsurance OR micro-insurance OR "micro insurance" OR "microgroup lending" OR microfranchis* OR micro-franchis* OR "micro franchis*" OR "micro credit*" OR microcredit* OR micro-credit* OR "micro enterprise*" OR microenterprise* OR micro-enterprise* OR "micro entrepreneur*" OR microentrepreneur* OR micro-entrepreneur* OR saving* OR micro-saving* OR microsaving* OR "Smallholder financ*" OR "rural financ*" OR "rural credit" OR roscas OR shgs OR "group lending" OR "community savings" OR "small loan*" OR "small lend*" OR ( ( bank* OR credit* ) W/3 cooperat* ) OR ( ( credit OR loan* OR lend* ) W/3 program* ) OR ( community W/3 ( bank* OR saving* OR loan* OR lend* ) ) OR "income generat*" OR grameen OR rosca* OR stokvel* OR ( ( financial OR economic ) W/2 ( literacy OR education OR skills OR training OR knowledge OR capab* ) ) OR banking OR budgeting OR "money manag*" OR "consumption smoothing" OR rationing OR earmarking OR "bank account*" OR "youth account*" OR "lock box*" OR "piggy bank*" OR "saving box*" OR ( ( access* OR participat* ) W/3 ( financ* OR credit OR saving* OR loan* OR lending ) ) OR "financial inclusion" OR "inclusive finance" OR fintech OR "mobile monies" OR m-pesa OR "mobile banking" OR cashless ) ) ) ) AND ( ( ( TITLE-ABS-KEY ( "literature search" OR "database search" OR "bibliographic* search" OR comprehensive AND search " OR extensive search" OR "exhaustive search" OR "purposive search" OR "representative search" OR "systemat* search" ) ) ) OR ( ( TITLE-ABS-KEY ( review W/3 ( effectiveness OR effects OR systemat* OR synth* OR integrat* OR map* OR methodologic* OR quantitative OR evidence OR literature ) ) ) ) OR ( ( TITLE-ABS-KEY ( "Meta regression" OR "meta synth*" OR "meta-synth*" OR "meta analy*" OR "metaanaly*" OR "meta-analy*" OR "metanaly*" OR "Metaregression" OR "Meta-regression" OR "Methodologic* overview" OR "pool* analys*" OR "pool* data" OR "Quantitative* overview" OR "research integration" ) ) ) OR ( ( TITLE-ABS-KEY ( ( systematic* OR synthes* ) W/3 ( research OR evaluation* OR finding* OR thematic* OR report OR descriptive OR explanatory OR narrative OR meta* OR review* OR data OR literature OR studies OR evidence OR map OR quantitative OR study OR studies OR paper OR impact OR impacts OR effect* OR compar* ) ) ) ) ) AND ( ( ( TITLE-ABS-KEY ( low* W/1 ( gdp OR gnp OR "gross domestic" OR "gross national" ) ) ) ) OR ( ( TITLE-ABS-KEY ( ( developing OR less* W/1 developed OR "under developed" OR underdeveloped OR "middle income" OR low* W/1 income ) W/1 ( economy OR economies ) ) ) ) OR ( ( TITLE-ABS-KEY ( ( developing OR ( less* W/1 developed ) OR "under developed" OR underdeveloped OR "middle income" OR ( low* W/1 income ) OR underserved OR "under served" OR deprived OR poor* ) W/1 ( countr* OR nation* OR population* OR world ) ) ) ) OR ( ( TITLE-ABS-KEY ( afghanistan OR albania OR algeria OR angola OR antigua OR barbuda OR argentina OR armenia OR armenian OR aruba OR azerbaijan OR bahrain OR bangladesh OR barbados OR benin OR belize OR bhutan OR bolivia OR botswana OR brazil OR brasil OR "Burkina Faso" OR "Burkina Fasso" OR "Upper Volta" OR burundi OR urundi OR cambodia OR "Khmer Republic" OR kampuchea OR cameroon OR cameroons OR cameron OR camerons OR "Cape Verde" OR "Central African Republic" OR chad OR chile OR china OR colombia OR comoros OR "Comoro Islands" OR comores OR mayotte OR congo OR zaire OR "Costa Rica" OR "Cote d'Ivoire" OR "Ivory Coast" OR cuba OR "Djibouti" OR "French Somaliland" OR dominica OR "Dominican Republic" OR "East Timor" OR "East Timur" OR "Timor Leste" OR ecuador OR egypt OR "United Arab Republic" OR "El Salvador" OR eritrea OR ethiopia OR fiji OR gabon OR "Gabonese Republic" OR gambia OR gaza OR "Georgia Republic" OR "Georgian Republic" OR ghana OR "Gold Coast" OR grenada OR guatemala OR guinea OR guam OR guiana OR guyana OR haiti OR honduras OR india OR maldives OR indonesia OR iran OR iraq OR jamaica OR jordan OR kazakhstan OR kazakh OR kenya OR kiribati OR korea OR kosovo OR kyrgyzstan OR kirghizia OR "Kyrgyz Republic" OR kirghiz OR kirgizstan OR "Lao PDR" OR laos OR lebanon OR lesotho OR basutoland OR liberia OR libya OR madagascar OR "Malagasy Republic" OR malaysia OR malaya OR malay OR sabah OR sarawak OR malawi OR nyasaland OR mali OR "Marshall Islands" OR mauritania OR mauritius OR "Agalega Islands" OR mexico OR micronesia OR "Middle East" OR moldova OR moldovia OR moldovian OR mongolia OR montenegro OR morocco OR ifni OR mozambique OR myanmar OR myanma OR burma OR namibia OR nepal OR antilles OR "New Caledonia" OR nicaragua OR niger OR nigeria OR "Mariana Islands" OR oman OR muscat OR pakistan OR palau OR palestine OR panama OR paraguay OR peru OR philippines OR philipines OR phillipines OR phillippines OR "Puerto Rico" OR rwanda OR ruanda OR "Saint Kitts" OR "St Kitts" OR nevis OR "Saint Lucia" OR "St Lucia" OR "Saint Vincent" OR "St Vincent" OR "Grenadines" OR "Samoa" OR "Samoan Islands" OR "Navigator Island" OR "Navigator Islands" OR "Sao Tome" OR "Saudi Arabia" OR senegal OR seychelles OR "Sierra Leone" OR "Sri Lanka" OR "Solomon Islands" OR somalia OR sudan OR suriname OR surinam OR swaziland OR syria OR tajikistan OR tadzhikistan OR tadjikistan OR tadzhik OR tanzania OR thailand OR togo OR "Togolese Republic" OR tonga OR trinidad OR tobago OR tunisia OR turkey OR turkmenistan OR turkmen OR uganda OR ukraine OR uruguay OR uzbekistan OR uzbek OR vanuatu OR "New Hebrides" OR venezuela OR vietnam OR "Viet Nam" OR "West Bank" OR yemen OR zambia OR zimbabwe OR jamahiriya OR jamahiryria OR libia OR mocambique OR principe OR syrian OR "Indian Ocean" OR melanesia OR "Western Sahara" ) ) ) OR ( ( ( TITLE-ABS-KEY ( africa OR asia OR caribbean OR "West Indies" OR "South America" OR "Latin America" OR "Central America" OR "Middle East" ) ) ) OR ( ( TITLE-ABS-KEY ( "transitional country" OR "transitional countries" ) ) ) OR ( ( TITLE-ABS-KEY ( lmic OR lmics OR "third world" OR "lami country" OR "lami countries" ) ) ) OR ( ( TITLE-ABS-KEY ( low W/3 middle W/3 countr* ) ) ) ) ) .

**1035 hits**

1. **Web of Science – Searched 14^th^ November 2017**

# 19 **2,014 hits**

#18 AND #8 AND #3 Indexes=SCI-EXPANDED, SSCI, A&HCI Timespan=2010-2017

# 18 5,049,601

#17 OR #16 OR #15 OR #14 OR #13 OR #12 OR #11 OR #10 OR #9

# 17 4,637,072

CU=(Afghanistan OR Albania OR Algeria OR Angola OR Antigua OR Barbuda OR Argentina OR Armenia OR Armenian OR Aruba OR Azerbaijan OR Bahrain OR Bangladesh OR Barbados OR Benin OR Belize OR Bhutan OR Bolivia OR Botswana OR Brazil OR Brasil OR "Burkina Faso" OR "Burkina Fasso" OR "Upper Volta" OR Burundi OR Urundi OR Cambodia OR "Khmer Republic" OR Kampuchea OR Cameroon OR Cameroons OR Cameron OR Camerons OR "Cape Verde" OR "Central African Republic" OR Chad OR Chile OR China OR Colombia OR Comoros OR "Comoro Islands" OR Comores OR Mayotte OR Congo OR Zaire OR "Costa Rica" OR "Cote d'Ivoire" OR "Ivory Coast" OR Cuba OR "Djibouti" OR "French Somaliland" OR Dominica OR "Dominican Republic" OR "East Timor" OR "East Timur" OR "Timor Leste" OR Ecuador OR Egypt OR "United Arab Republic" OR "El Salvador" OR Eritrea OR Ethiopia OR Fiji OR Gabon OR "Gabonese Republic" OR Gambia OR Gaza OR "Georgia Republic" OR "Georgian Republic" OR Ghana OR "Gold Coast" OR Grenada OR Guatemala OR Guinea OR Guam OR Guiana OR Guyana OR Haiti OR Honduras OR India OR Maldives OR Indonesia OR Iran OR Iraq OR Jamaica OR Jordan OR Kazakhstan OR Kazakh OR Kenya OR Kiribati OR Korea OR Kosovo OR Kyrgyzstan OR Kirghizia OR "Kyrgyz Republic" OR Kirghiz OR Kirgizstan OR "Lao PDR" OR Laos OR Lebanon OR Lesotho OR Basutoland OR Liberia OR Libya OR Madagascar OR "Malagasy Republic" OR Malaysia OR Malaya OR Malay OR Sabah OR Sarawak OR Malawi OR Nyasaland OR Mali OR "Marshall Islands" OR Mauritania OR Mauritius OR "Agalega Islands" OR Mexico OR Micronesia OR "Middle East" OR Moldova OR Moldovia OR Moldovian OR Mongolia OR Montenegro OR Morocco OR Ifni OR Mozambique OR Myanmar OR Myanma OR Burma OR Namibia OR Nepal OR Antilles OR "New Caledonia" OR Nicaragua OR Niger OR Nigeria OR "Mariana Islands" OR Oman OR Muscat OR Pakistan OR Palau OR Palestine OR Panama OR Paraguay OR Peru OR Philippines OR Philipines OR Phillipines OR Phillippines OR "Puerto Rico" OR Rwanda OR Ruanda OR "Saint Kitts" OR "St Kitts" OR Nevis OR "Saint Lucia" OR "St Lucia" OR "Saint Vincent" OR "St Vincent" OR "Grenadines" OR "Samoa" OR "Samoan Islands" OR "Navigator Island" OR "Navigator Islands" OR "Sao Tome" OR "Saudi Arabia" OR Senegal OR Seychelles OR "Sierra Leone" OR "Sri Lanka" OR "Solomon Islands" OR Somalia OR Sudan OR Suriname OR Surinam OR Swaziland OR Syria OR Tajikistan OR Tadzhikistan OR Tadjikistan OR Tadzhik OR Tanzania OR Thailand OR Togo OR "Togolese Republic" OR Tonga OR Trinidad OR Tobago OR Tunisia OR Turkey OR Turkmenistan OR Turkmen OR Uganda OR Ukraine OR Uruguay OR Uzbekistan OR Uzbek OR Vanuatu OR "New Hebrides" OR Venezuela OR Vietnam OR "Viet Nam" OR "West Bank" OR Yemen OR Zambia OR Zimbabwe OR Jamahiriya OR Jamahiryria OR Libia OR Mocambique OR Principe OR Syrian OR "Indian Ocean" OR Melanesia OR "Western Sahara")

# 16 971,268

TS=(Afghanistan OR Albania OR Algeria OR Angola OR Antigua OR Barbuda OR Argentina OR Armenia OR Armenian OR Aruba OR Azerbaijan OR Bahrain OR Bangladesh OR Barbados OR Benin OR Belize OR Bhutan OR Bolivia OR Botswana OR Brazil OR Brasil OR "Burkina Faso" OR "Burkina Fasso" OR "Upper Volta" OR Burundi OR Urundi OR Cambodia OR "Khmer Republic" OR Kampuchea OR Cameroon OR Cameroons OR Cameron OR Camerons OR "Cape Verde" OR "Central African Republic" OR Chad OR Chile OR China OR Colombia OR Comoros OR "Comoro Islands" OR Comores OR Mayotte OR Congo OR Zaire OR "Costa Rica" OR "Cote d'Ivoire" OR "Ivory Coast" OR Cuba OR "Djibouti" OR "French Somaliland" OR Dominica OR "Dominican Republic" OR "East Timor" OR "East Timur" OR "Timor Leste" OR Ecuador OR Egypt OR "United Arab Republic" OR "El Salvador" OR Eritrea OR Ethiopia OR Fiji OR Gabon OR "Gabonese Republic" OR Gambia OR Gaza OR "Georgia Republic" OR "Georgian Republic" OR Ghana OR "Gold Coast" OR Grenada OR Guatemala OR Guinea OR Guam OR Guiana OR Guyana OR Haiti OR Honduras OR India OR Maldives OR Indonesia OR Iran OR Iraq OR Jamaica OR Jordan OR Kazakhstan OR Kazakh OR Kenya OR Kiribati OR Korea OR Kosovo OR Kyrgyzstan OR Kirghizia OR "Kyrgyz Republic" OR Kirghiz OR Kirgizstan OR "Lao PDR" OR Laos OR Lebanon OR Lesotho OR Basutoland OR Liberia OR Libya OR Madagascar OR "Malagasy Republic" OR Malaysia OR Malaya OR Malay OR Sabah OR Sarawak OR Malawi OR Nyasaland OR Mali OR "Marshall Islands" OR Mauritania OR Mauritius OR "Agalega Islands" OR Mexico OR Micronesia OR "Middle East" OR Moldova OR Moldovia OR Moldovian OR Mongolia OR Montenegro OR Morocco OR Ifni OR Mozambique OR Myanmar OR Myanma OR Burma OR Namibia OR Nepal OR Antilles OR "New Caledonia" OR Nicaragua OR Niger OR Nigeria OR "Mariana Islands" OR Oman OR Muscat OR Pakistan OR Palau OR Palestine OR Panama OR Paraguay OR Peru OR Philippines OR Philipines OR Phillipines OR Phillippines OR "Puerto Rico" OR Rwanda OR Ruanda OR "Saint Kitts" OR "St Kitts" OR Nevis OR "Saint Lucia" OR "St Lucia" OR "Saint Vincent" OR "St Vincent" OR "Grenadines" OR "Samoa" OR "Samoan Islands" OR "Navigator Island" OR "Navigator Islands" OR "Sao Tome" OR "Saudi Arabia" OR Senegal OR Seychelles OR "Sierra Leone" OR "Sri Lanka" OR "Solomon Islands" OR Somalia OR Sudan OR Suriname OR Surinam OR Swaziland OR Syria OR Tajikistan OR Tadzhikistan OR Tadjikistan OR Tadzhik OR Tanzania OR Thailand OR Togo OR "Togolese Republic" OR Tonga OR Trinidad OR Tobago OR Tunisia OR Turkey OR Turkmenistan OR Turkmen OR Uganda OR Ukraine OR Uruguay OR Uzbekistan OR Uzbek OR Vanuatu OR "New Hebrides" OR Venezuela OR Vietnam OR "Viet Nam" OR "West Bank" OR Yemen OR Zambia OR Zimbabwe OR Jamahiriya OR Jamahiryria OR Libia OR Mocambique OR Principe OR Syrian OR "Indian Ocean" OR Melanesia OR "Western Sahara")

# 15 122,678

TS=((developing OR (less* NEAR developed) OR "under developed" OR underdeveloped OR "middle income" or (low* NEAR income)) NEAR (countr* or nation* or population* or world))

# 14 5,815

TS=((developing OR (less* NEAR developed) OR "under developed" OR underdeveloped OR "middle income" or (low* NEAR income)) NEAR (economy or economies))

# 13 1,137

TS=( low* NEAR (gdp OR gnp OR "gross domestic" OR "gross national") )

# 12 7,869

TS=(low NEAR/3 middle NEAR/3 countr*)

# 11 2,756

TS=(lmic OR lmics OR "third world" OR "lami country" OR "lami countries")

# 10 169

TS=("transitional country" OR "transitional countries")

# 9 222,677

TS=(Africa OR Asia OR Caribbean OR "West Indies" OR "South America" OR "Latin America" OR "Central America" OR "Middle East")

# 8 555,428

#7 OR #6 OR #5 OR #4

# 7 264,439

TS=(((Systematic* OR synthes*) NEAR/3 (Research OR evaluation* OR finding* OR thematic* OR report OR descriptive OR explanatory OR narrative OR meta* OR review* OR data OR literature OR evidence OR map OR quantitative OR study OR studies OR paper OR impact OR impacts OR effect* OR compar*)))

# 6 213,939

TS=("Meta regression" OR "meta synth*" OR "meta-synth*" OR "meta analy*" OR "metaanaly*" OR "meta-analy*" OR "metanaly*" OR "Metaregression" OR "Meta-regression" OR "Methodologic* overview" OR "pool* analys*" OR "pool* data" OR "Quantitative* overview" OR "research integration")

# 5 223,106

TS=((review NEAR/3 (effectiveness OR effects OR systemat* OR synth* OR integrat* OR map* OR methodologic* OR quantitative OR evidence OR literature)))

# 4 32,845

TS=("literature search" OR "database search" OR "bibliographic* search" OR "comprehensive search" OR "extensive search" OR "exhaustive search" OR "purposive search" OR "representative search" OR "systemat* search")

# 3 160,645

#2 OR #1

# 2 157,779

TS=(micro-finance OR "micro finance" OR microfinance OR micro-loan* OR microloan* OR "micro loan*" OR microleas* OR micro-leas* OR "micro leas*" OR microlending OR micro-lending OR "micro lending" OR microinsurance OR micro-insurance OR "micro insurance" OR "microgroup lending" OR microfranchis* OR micro-franchis* OR "micro franchis*" OR "micro credit*" OR microcredit* OR micro-credit* OR "micro enterprise*" OR microenterprise* OR micro-enterprise* OR "micro entrepreneur*" OR microentrepreneur* OR micro-entrepreneur* OR saving* OR micro-saving* OR microsaving* OR "Smallholder financ*" OR "rural financ*" OR "rural credit" OR SHGs OR "group lending" OR "community savings" OR "small loan*" OR "small lend*" OR ((bank or credit*) NEAR/3 cooperat*) OR ((credit OR loan* OR lend*) NEAR/3 program*) OR (community NEAR/3 (bank* OR saving* OR loan* OR lend*)) OR "income generat*" OR grameen OR ROSCA* OR stokvel* OR ((financial OR economic) NEAR/2 (literacy OR education OR skills OR training OR knowledge OR capab*)) OR banking OR budgeting OR "money manag*" OR "consumption smoothing" OR rationing OR earmarking OR "bank account*" OR "youth account*" OR "lock box*" OR "piggy bank*" OR "saving box*" OR ((access* OR participat*) NEAR/3 ( financ* OR credit OR saving* OR loan* OR lending)) OR "financial inclusion" OR "inclusive finance" OR fintech OR "mobile monies" OR M-PESA OR "mobile banking" OR cashless )

# 1 2,919

TS=(mhealth OR "mobile health" OR m-health)

1. **Business Source Premier (EBSCO) – Searched 18^th^ January 2018**

S23 S11 AND S16 AND S21 Limiters - Published Date: 20100101-20181231

**408 hits**

S22 S16 AND S21

1,142

S21 S17 OR S18 OR S19 OR S20

238,223

S20 TI ( ( mhealth or "mobile health" or m-health ) ) OR AB ( ( mhealth or "mobile health" or m-health ) ) OR SU ( ( mhealth or "mobile health" or m-health ) )

629

S19 SU (micro-finance OR "micro finance" OR microfinance OR micro-loan* OR microloan* OR "micro loan*" OR microleas* OR micro-leas* OR "micro leas*" OR microlending OR micro-lending OR "micro lending" OR microinsurance OR micro-insurance OR "micro insurance" OR "microgroup lending" OR microfranchis* OR micro-franchis* OR "micro franchis*" OR "micro credit*" OR microcredit* OR micro-credit* OR "micro enterprise*" OR microenterprise* OR micro-enterprise* OR "micro entrepreneur*" OR microentrepreneur* OR micro-entrepreneur* OR saving* OR micro-saving* OR microsaving* OR "Smallholder financ*" OR "rural financ*" OR "rural credit" OR ROSCAs OR SHGs OR "group lending" OR "community savings" or "small loan*" or "small lend*" or ((bank or credit*) N3 cooperat*) or ((credit or loan* or lend*) N3 program*) or (community N3 (bank* or saving* or loan* or lend*)) or "income generat*" or grameen OR ROSCA* OR stokvel* OR ((financial OR economic) N2 (literacy OR education OR skills OR training OR knowledge OR capab*)) OR banking OR budgeting OR "money manag*" OR "consumption smoothing" OR rationing OR earmarking OR "bank account*" OR "youth account*" OR "lock box*" OR "piggy bank*" OR "saving box*" OR ((access* OR participat*) N3 ( financ* OR credit OR saving* OR loan* OR lending)) OR "financial inclusion" OR "inclusive finance" OR fintech OR "mobile monies" OR M-PESA OR "mobile banking" OR cashless )

133,480

S18 AB (micro-finance OR "micro finance" OR microfinance OR micro-loan* OR microloan* OR "micro loan*" OR microleas* OR micro-leas* OR "micro leas*" OR microlending OR micro-lending OR "micro lending" OR microinsurance OR micro-insurance OR "micro insurance" OR "microgroup lending" OR microfranchis* OR micro-franchis* OR "micro franchis*" OR "micro credit*" OR microcredit* OR micro-credit* OR "micro enterprise*" OR microenterprise* OR micro-enterprise* OR "micro entrepreneur*" OR microentrepreneur* OR micro-entrepreneur* OR saving* OR micro-saving* OR microsaving* OR "Smallholder financ*" OR "rural financ*" OR "rural credit" OR SHGs OR "group lending" OR "community savings" or "small loan*" or "small lend*" or ((bank or credit*) N3 cooperat*) or ((credit or loan* or lend*) N3 program*) or (community N3 (bank* or saving* or loan* or lend*)) or "income generat*" or grameen OR ROSCA* OR stokvel* OR ((financial OR economic) N2 (literacy OR education OR skills OR training OR knowledge OR capab*)) OR banking OR budgeting OR "money manag*" OR "consumption smoothing" OR rationing OR earmarking OR "bank account*" OR "youth account*" OR "lock box*" OR "piggy bank*" OR "saving box*" OR ((access* OR participat*) N3 ( financ* OR credit OR saving* OR loan* OR lending)) OR "financial inclusion" OR "inclusive finance" OR fintech OR "mobile monies" OR M-PESA OR "mobile banking" OR cashless)

155,180

S17 TI (micro-finance OR "micro finance" OR microfinance OR micro-loan* OR microloan* OR "micro loan*" OR microleas* OR micro-leas* OR "micro leas*" OR microlending OR micro-lending OR "micro lending" OR microinsurance OR micro-insurance OR "micro insurance" OR "microgroup lending" OR microfranchis* OR micro-franchis* OR "micro franchis*" OR "micro credit*" OR microcredit* OR micro-credit* OR "micro enterprise*" OR microenterprise* OR micro-enterprise* OR "micro entrepreneur*" OR microentrepreneur* OR micro-entrepreneur* OR saving* OR micro-saving* OR microsaving* OR "Smallholder financ*" OR "rural financ*" OR "rural credit" OR SHGs OR "group lending" OR "community savings" or "small loan*" or "small lend*" or ((bank or credit*) N3 cooperat*) or ((credit or loan* or lend*) N3 program*) or (community N3 (bank* or saving* or loan* or lend*)) or "income generat*" or grameen OR ROSCA* OR stokvel* OR ((financial OR economic) N2 (literacy OR education OR skills OR training OR knowledge OR capab*)) OR banking OR budgeting OR "money manag*" OR "consumption smoothing" OR rationing OR earmarking OR "bank account*" OR "youth account*" OR "lock box*" OR "piggy bank*" OR "saving box*" OR ((access* OR participat*) N3 ( financ* OR credit OR saving* OR loan* OR lending)) OR "financial inclusion" OR "inclusive finance" OR fintech OR "mobile monies" OR M-PESA OR "mobile banking" OR cashless )

36,831

S16 S12 or S13 or S14 or S15

30,115

S15 TI ( ("literature search" OR "database search" OR "bibliographic* search" OR "comprehensive search" OR "extensive search" OR "exhaustive search" OR "purposive search" OR "representative search" or "systemat* search") ) OR AB ( ("literature search" OR "database search" OR "bibliographic* search" OR "comprehensive search" OR "extensive search" OR "exhaustive search" OR "purposive search" OR "representative search" or "systemat* search") ) OR SU ( ("literature search" OR "database search" OR "bibliographic* search" OR "comprehensive search" OR "extensive search" OR "exhaustive search" OR "purposive search" OR "representative search" or "systemat* search") )

863

S14 TI ( (review N3 (effectiveness OR effects OR systemat* OR synth* OR integrat* OR map* OR methodologic* OR quantitative OR evidence OR literature)) ) OR AB ( (review N3 (effectiveness OR effects OR systemat* OR synth* OR integrat* OR map* OR methodologic* OR quantitative OR evidence OR literature)) ) OR SU ( (review N3 (effectiveness OR effects OR systemat* OR synth* OR integrat* OR map* OR methodologic* OR quantitative OR evidence OR literature)) )

18,688

S13 TI ( ("Meta regression" OR "meta synth*" OR "meta-synth*" OR "meta analy*" OR "metaanaly*" OR "meta-analy*" OR "metanaly*" OR "Metaregression" OR "Meta-regression" OR "Methodologic* overview" OR "pool* analys*" OR "pool* data" OR "Quantitative* overview" OR "research integration") ) OR AB ( ("Meta regression" OR "meta synth*" OR "meta-synth*" OR "meta analy*" OR "metaanaly*" OR "meta-analy*" OR "metanaly*" OR "Metaregression" OR "Meta-regression" OR "Methodologic* overview" OR "pool* analys*" OR "pool* data" OR "Quantitative* overview" OR "research integration") ) OR SU ( ("Meta regression" OR "meta synth*" OR "meta-synth*" OR "meta analy*" OR "metaanaly*" OR "meta-analy*" OR "metanaly*" OR "Metaregression" OR "Meta-regression" OR "Methodologic* overview" OR "pool* analys*" OR "pool* data" OR "Quantitative* overview" OR "research integration") )

4,335

S12 TI ( ((Systematic* OR synthes*) N3 (Research OR evaluation* OR finding* OR thematic* OR report OR descriptive OR explanatory OR narrative OR meta* OR review* OR data OR literature OR studies OR evidence OR map OR quantitative OR study OR studies OR paper OR impact OR impacts OR effect* OR compar*)) ) OR AB ( ((Systematic* OR synthes*) N3 (Research OR evaluation* OR finding* OR thematic* OR report OR descriptive OR explanatory OR narrative OR meta* OR review* OR data OR literature OR studies OR evidence OR map OR quantitative OR study OR studies OR paper OR impact OR impacts OR effect* OR compar*)) ) OR SU ( ((Systematic* OR synthes*) N3 (Research OR evaluation* OR finding* OR thematic* OR report OR descriptive OR explanatory OR narrative OR meta* OR review* OR data OR literature OR studies OR evidence OR map OR quantitative OR study OR studies OR paper OR impact OR impacts OR effect* OR compar*)) )

11,688

S11 S1 OR S2 OR S3 OR S4 OR S5 OR S6 OR S7 OR S8 OR S9 OR S10

1,066,885

S10 TI (Africa or Asia or Caribbean or "West Indies" or "South America" or "Latin America" or "Central America" OR "Middle East") OR AB (Africa or Asia or Caribbean or "West Indies" or "South America" or "Latin America" or "Central America" OR "Middle East") OR SU (Africa or Asia or Caribbean or "West Indies" or "South America" or "Latin America" or "Central America" OR "Middle East") OR GE (Africa or Asia or Caribbean or "West Indies" or "South America" or "Latin America" or "Central America" OR "Middle East")

156,848

S9 TI ( ("transitional country" or "transitional countries") ) OR AB ( ("transitional country" or "transitional countries") ) OR SU ( ("transitional country" or "transitional countries") )

88

S8 TI ( (lmic or lmics or "third world" or "lami country" or "lami countries") ) OR AB ( (lmic or lmics or "third world" or "lami country" or "lami countries") ) OR SU ( (lmic or lmics or "third world" or "lami country" or "lami countries") )

570

S7 TI (low N3 middle N3 countr*) OR AB (low N3 middle N3 countr*) OR SU (low N3 middle N3 countr*)

758

S6 TI ( low* N1 (gdp or gnp or "gross domestic" or "gross national") ) OR AB ( low* N1 (gdp or gnp or "gross domestic" or "gross national") ) OR SU ( low* N1 (gdp or gnp or "gross domestic" or "gross national") )

237

S5 TI ( (developing or less* N1 developed or "under developed" or underdeveloped or "middle income" or low* N1 income) N1 (economy or economies) ) OR AB ( (developing or less* N1 developed or "under developed" or underdeveloped or "middle income" or low* N1 income) N1 (economy or economies) ) OR SU ( (developing or less* N1 developed or "under developed" or underdeveloped or "middle income" or low* N1 income) N1 (economy or economies) )

2,346

S4 TI ( (developing or less* N1 developed or "under developed" or underdeveloped or "middle income" or low* N1 income or underserved or "under served" or deprived or poor*) N1 (countr* or nation* or population* or world) ) OR AB ( (developing or less* N1 developed or "under developed" or underdeveloped or "middle income" or low* N1 income or underserved or "under served" or deprived or poor*) N1 (countr* or nation* or population* or world) ) OR SU ( (developing or less* N1 developed or "under developed" or underdeveloped or "middle income" or low* N1 income or underserved or "under served" or deprived or poor*) N1 (countr* or nation* or population* or world) )

22,624

S3 AB Afghanistan OR Albania OR Algeria OR Angola OR Antigua OR Barbuda OR Argentina OR Armenia OR Armenian OR Aruba OR Azerbaijan OR Bahrain OR Bangladesh OR Barbados OR Benin OR Belize OR Bhutan OR Bolivia OR Botswana OR Brazil OR Brasil OR "Burkina Faso" OR "Burkina Fasso" OR "Upper Volta" OR Burundi OR Urundi OR Cambodia OR "Khmer Republic" OR Kampuchea OR Cameroon OR Cameroons OR Cameron OR Camerons OR "Cape Verde" OR "Central African Republic" OR Chad OR Chile OR China OR Colombia OR Comoros OR "Comoro Islands" OR Comores OR Mayotte OR Congo OR Zaire OR "Costa Rica" OR "Cote d'Ivoire" OR "Ivory Coast" OR Cuba OR "Djibouti" OR "French Somaliland" OR Dominica OR "Dominican Republic" OR "East Timor" OR "East Timur" OR "Timor Leste" OR Ecuador OR Egypt OR "United Arab Republic" OR "El Salvador" OR Eritrea OR Ethiopia OR Fiji OR Gabon OR "Gabonese Republic" OR Gambia OR Gaza OR "Georgia Republic" OR "Georgian Republic" OR Ghana OR "Gold Coast" OR Grenada OR Guatemala OR Guinea OR Guam OR Guiana OR Guyana OR Haiti OR Honduras OR India OR Maldives OR Indonesia OR Iran OR Iraq OR Jamaica OR Jordan OR Kazakhstan OR Kazakh OR Kenya OR Kiribati OR Korea OR Kosovo OR Kyrgyzstan OR Kirghizia OR "Kyrgyz Republic" OR Kirghiz OR Kirgizstan OR "Lao PDR" OR Laos OR Lebanon OR Lesotho OR Basutoland OR Liberia OR Libya OR Madagascar OR "Malagasy Republic" OR Malaysia OR Malaya OR Malay OR Sabah OR Sarawak OR Malawi OR Nyasaland OR Mali OR "Marshall Islands" OR Mauritania OR Mauritius OR "Agalega Islands" OR Mexico OR Micronesia OR "Middle East" OR Moldova OR Moldovia OR Moldovian OR Mongolia OR Montenegro OR Morocco OR Ifni OR Mozambique OR Myanmar OR Myanma OR Burma OR Namibia OR Nepal OR Antilles OR "New Caledonia" OR Nicaragua OR Niger OR Nigeria OR "Mariana Islands" OR Oman OR Muscat OR Pakistan OR Palau OR Palestine OR Panama OR Paraguay OR Peru OR Philippines OR Philipines OR Phillipines OR Phillippines OR "Puerto Rico" OR Rwanda OR Ruanda OR "Saint Kitts" OR "St Kitts" OR Nevis OR "Saint Lucia" OR "St Lucia" OR "Saint Vincent" OR "St Vincent" OR "Grenadines" OR "Samoa" OR "Samoan Islands" OR "Navigator Island" OR "Navigator Islands" OR "Sao Tome" OR "Saudi Arabia" OR Senegal OR Seychelles OR "Sierra Leone" OR "Sri Lanka" OR "Solomon Islands" OR Somalia OR Sudan OR Suriname OR Surinam OR Swaziland OR Syria OR Tajikistan OR Tadzhikistan OR Tadjikistan OR Tadzhik OR Tanzania OR Thailand OR Togo OR "Togolese Republic" OR Tonga OR Trinidad OR Tobago OR Tunisia OR Turkey OR Turkmenistan OR Turkmen OR Uganda OR Ukraine OR Uruguay OR Uzbekistan OR Uzbek OR Vanuatu OR "New Hebrides" OR Venezuela OR Vietnam OR "Viet Nam" OR "West Bank" OR Yemen OR Zambia OR Zimbabwe OR Jamahiriya OR Jamahiryria OR Libia OR Mocambique OR Principe OR Syrian OR "Indian Ocean" OR Melanesia OR "Western Sahara"

973,710

S2 TI Afghanistan OR Albania OR Algeria OR Angola OR Antigua OR Barbuda OR Argentina OR Armenia OR Armenian OR Aruba OR Azerbaijan OR Bahrain OR Bangladesh OR Barbados OR Benin OR Belize OR Bhutan OR Bolivia OR Botswana OR Brazil OR Brasil OR "Burkina Faso" OR "Burkina Fasso" OR "Upper Volta" OR Burundi OR Urundi OR Cambodia OR "Khmer Republic" OR Kampuchea OR Cameroon OR Cameroons OR Cameron OR Camerons OR "Cape Verde" OR "Central African Republic" OR Chad OR Chile OR China OR Colombia OR Comoros OR "Comoro Islands" OR Comores OR Mayotte OR Congo OR Zaire OR "Costa Rica" OR "Cote d'Ivoire" OR "Ivory Coast" OR Cuba OR "Djibouti" OR "French Somaliland" OR Dominica OR "Dominican Republic" OR "East Timor" OR "East Timur" OR "Timor Leste" OR Ecuador OR Egypt OR "United Arab Republic" OR "El Salvador" OR Eritrea OR Ethiopia OR Fiji OR Gabon OR "Gabonese Republic" OR Gambia OR Gaza OR "Georgia Republic" OR "Georgian Republic" OR Ghana OR "Gold Coast" OR Grenada OR Guatemala OR Guinea OR Guam OR Guiana OR Guyana OR Haiti OR Honduras OR India OR Maldives OR Indonesia OR Iran OR Iraq OR Jamaica OR Jordan OR Kazakhstan OR Kazakh OR Kenya OR Kiribati OR Korea OR Kosovo OR Kyrgyzstan OR Kirghizia OR "Kyrgyz Republic" OR Kirghiz OR Kirgizstan OR "Lao PDR" OR Laos OR Lebanon OR Lesotho OR Basutoland OR Liberia OR Libya OR Madagascar OR "Malagasy Republic" OR Malaysia OR Malaya OR Malay OR Sabah OR Sarawak OR Malawi OR Nyasaland OR Mali OR "Marshall Islands" OR Mauritania OR Mauritius OR "Agalega Islands" OR Mexico OR Micronesia OR "Middle East" OR Moldova OR Moldovia OR Moldovian OR Mongolia OR Montenegro OR Morocco OR Ifni OR Mozambique OR Myanmar OR Myanma OR Burma OR Namibia OR Nepal OR Antilles OR "New Caledonia" OR Nicaragua OR Niger OR Nigeria OR "Mariana Islands" OR Oman OR Muscat OR Pakistan OR Palau OR Palestine OR Panama OR Paraguay OR Peru OR Philippines OR Philipines OR Phillipines OR Phillippines OR "Puerto Rico" OR Rwanda OR Ruanda OR "Saint Kitts" OR "St Kitts" OR Nevis OR "Saint Lucia" OR "St Lucia" OR "Saint Vincent" OR "St Vincent" OR "Grenadines" OR "Samoa" OR "Samoan Islands" OR "Navigator Island" OR "Navigator Islands" OR "Sao Tome" OR "Saudi Arabia" OR Senegal OR Seychelles OR "Sierra Leone" OR "Sri Lanka" OR "Solomon Islands" OR Somalia OR Sudan OR Suriname OR Surinam OR Swaziland OR Syria OR Tajikistan OR Tadzhikistan OR Tadjikistan OR Tadzhik OR Tanzania OR Thailand OR Togo OR "Togolese Republic" OR Tonga OR Trinidad OR Tobago OR Tunisia OR Turkey OR Turkmenistan OR Turkmen OR Uganda OR Ukraine OR Uruguay OR Uzbekistan OR Uzbek OR Vanuatu OR "New Hebrides" OR Venezuela OR Vietnam OR "Viet Nam" OR "West Bank" OR Yemen OR Zambia OR Zimbabwe OR Jamahiriya OR Jamahiryria OR Libia OR Mocambique OR Principe OR Syrian OR "Indian Ocean" OR Melanesia OR "Western Sahara"

971,403

S1 SU Afghanistan OR Albania OR Algeria OR Angola OR Antigua OR Barbuda OR Argentina OR Armenia OR Armenian OR Aruba OR Azerbaijan OR Bahrain OR Bangladesh OR Barbados OR Benin OR Belize OR Bhutan OR Bolivia OR Botswana OR Brazil OR Brasil OR "Burkina Faso" OR "Burkina Fasso" OR "Upper Volta" OR Burundi OR Urundi OR Cambodia OR "Khmer Republic" OR Kampuchea OR Cameroon OR Cameroons OR Cameron OR Camerons OR "Cape Verde" OR "Central African Republic" OR Chad OR Chile OR China OR Colombia OR Comoros OR "Comoro Islands" OR Comores OR Mayotte OR Congo OR Zaire OR "Costa Rica" OR "Cote d'Ivoire" OR "Ivory Coast" OR Cuba OR "Djibouti" OR "French Somaliland" OR Dominica OR "Dominican Republic" OR "East Timor" OR "East Timur" OR "Timor Leste" OR Ecuador OR Egypt OR "United Arab Republic" OR "El Salvador" OR Eritrea OR Ethiopia OR Fiji OR Gabon OR "Gabonese Republic" OR Gambia OR Gaza OR "Georgia Republic" OR "Georgian Republic" OR Ghana OR "Gold Coast" OR Grenada OR Guatemala OR Guinea OR Guam OR Guiana OR Guyana OR Haiti OR Honduras OR India OR Maldives OR Indonesia OR Iran OR Iraq OR Jamaica OR Jordan OR Kazakhstan OR Kazakh OR Kenya OR Kiribati OR Korea OR Kosovo OR Kyrgyzstan OR Kirghizia OR "Kyrgyz Republic" OR Kirghiz OR Kirgizstan OR "Lao PDR" OR Laos OR Lebanon OR Lesotho OR Basutoland OR Liberia OR Libya OR Madagascar OR "Malagasy Republic" OR Malaysia OR Malaya OR Malay OR Sabah OR Sarawak OR Malawi OR Nyasaland OR Mali OR "Marshall Islands" OR Mauritania OR Mauritius OR "Agalega Islands" OR Mexico OR Micronesia OR "Middle East" OR Moldova OR Moldovia OR Moldovian OR Mongolia OR Montenegro OR Morocco OR Ifni OR Mozambique OR Myanmar OR Myanma OR Burma OR Namibia OR Nepal OR Antilles OR "New Caledonia" OR Nicaragua OR Niger OR Nigeria OR "Mariana Islands" OR Oman OR Muscat OR Pakistan OR Palau OR Palestine OR Panama OR Paraguay OR Peru OR Philippines OR Philipines OR Phillipines OR Phillippines OR "Puerto Rico" OR Rwanda OR Ruanda OR "Saint Kitts" OR "St Kitts" OR Nevis OR "Saint Lucia" OR "St Lucia" OR "Saint Vincent" OR "St Vincent" OR "Grenadines" OR "Samoa" OR "Samoan Islands" OR "Navigator Island" OR "Navigator Islands" OR "Sao Tome" OR "Saudi Arabia" OR Senegal OR Seychelles OR "Sierra Leone" OR "Sri Lanka" OR "Solomon Islands" OR Somalia OR Sudan OR Suriname OR Surinam OR Swaziland OR Syria OR Tajikistan OR Tadzhikistan OR Tadjikistan OR Tadzhik OR Tanzania OR Thailand OR Togo OR "Togolese Republic" OR Tonga OR Trinidad OR Tobago OR Tunisia OR Turkey OR Turkmenistan OR Turkmen OR Uganda OR Ukraine OR Uruguay OR Uzbekistan OR Uzbek OR Vanuatu OR "New Hebrides" OR Venezuela OR Vietnam OR "Viet Nam" OR "West Bank" OR Yemen OR Zambia OR Zimbabwe OR Jamahiriya OR Jamahiryria OR Libia OR Mocambique OR Principe OR Syrian OR "Indian Ocean" OR Melanesia OR "Western Sahara"

972,854

**8. Grey literature search strategy – Searched 24 January – 10 February 2018**

The following simplified series of search strings were developed for searching through the grey literature, wherein the search engines are not as sophisticated as the academic databases, and thus cannot handle the same search strategy. The conversion of the academic search strategy was undertaken through the following steps:

1. First, the most relevant terms were extracted from the intervention and methodology sections of the complete search strategy applied to the academic databases. Population terms were not included because the advanced search options within the search engines was not sophisticated enough to allow for an “or” limiter for each LMIC.
2. These were then crosschecked with the titles of the pre-identified reviews for consideration to identify the most common terms in each section. Since each intervention term had to be searched individually against each methodology term, these were limited to the seven and four most common terms, respectively. Where possible, the methodology terms were limited to the abstract. Intervention terms were not limited, so as to increase the likelihood of identification of relevant studies that were focused on similar terms but mentioned the common term only in the main body.
3. For specialist microfinance sites, wherein all papers were assumed to be topically relevant, only methodology searches were run, thus, four per site. For the remaining grey literature sites, each intervention term was run against each methodology term on each site for a total of 32 searches per site, with a few exceptions as noted below. In total, 348 searches were run overall.
4. Where possible, display settings were set to show 50 results per page, and the first page of results was printed to PDF. Each set of PDFs was saved in a folder with the site name and date of search.
5. For each set of results, the titles were screened for potential inclusion, and if clearly irrelevant based on content, methodology or population, were ignored. If the title indicated possible relevance, the abstract was screened for the same.
6. Any result that appeared of potential relevance was then downloaded and saved in the folder for that site.
7. The documents collected from the grey literature site were then added to the master EndNote for the Review of Reviews.

*Simplified search strings:*

Intervention terms:

- microfinance
- “financial inclusion”
- “inclusive finance”
- “access to finance”
- micro-finance
- micro-credit
- microcredit
- mHealth

Methodology terms

- “systematic review”
- meta-analysis
- evidence synthesis *(no quotation marks if restricted to abstract)*
- “effectiveness review”

**Grey literature sites searched:**

NB: Searches were conducted directly from the links noted, with cumulative hits from each of the search strings noted in brackets.

*Microfinance specific institutions and web portals:*

- CGAP: [www.cgap.org](http://www.cgap.org) (6)
- Microbanking Bulletin: [www.themix.org](http://www.themix.org) (0)
- Microfinance Gateway: [www.microfinancegateway.org](http://www.microfinancegateway.org) (10)
- SEEP: <http://www.seepnetwork.org> (1)
  - This site’s search doesn’t differentiate between a search for *meta analysis* and *meta-analysis*, and thus quotation marks were applied to ensure relevance.
- Grameen Foundation: <https://www.grameenfoundation.org/resources/publications> (0)
- BRAC Research and Evaluation Division: <http://research.brac.net/new/publications> (0)
- Alliance for Financial Inclusion: <https://www.afi-global.org/publications/> (0)
- Accion Center for Financial Inclusion: <http://www.centerforfinancialinclusion.org/index.php> (0)

*Multilateral and bilateral and non-governmental donor organizations:*

- World Bank (WB e-library was searched within EBSCO’s Discovery Service but will also be searched and screened online via the World Bank’s website): <https://elibrary.worldbank.org/action/doSearch> (102)
- African Development Bank: <https://www.afdb.org/en/search/> (145)
  - **Search note:** Search results limited to “Document” type.
  - **Search note:** The AfDB search function does not distinguish between *meta-analysis* and *meta analysis*, thus *“meta-analysis”* was searched to ensure relevant results.
  - **Exclusion note:** AfDB carries out annual “effectiveness reviews” of their programming, but these are not eligible for inclusion because they are based on internal Results Measurement Framework data, and do not constitute syntheses of impact evaluations.
- Asian Development Bank: <http://www.adb.org> (938)
  - **Search note:** Search results limited to types “Evaluation Document;” “Institutional Document;” and “Publication.”
  - **Exclusion note:** ADB carries out annual “effectiveness reviews” of their programming, but these are not eligible for inclusion because they do not constitute syntheses of impact evaluations.
- Inter-American Development Bank: <https://publications.iadb.org/facet-view?field=type_view> (4)
  - **Search note:** additional searches in Spanish for “revisión de literatura" were conducted. This is because though the technical terms appeared to be translated and the translations included in search results (“financial inclusion” returned the same results as “inclusión financiera”), the same was not true for the study design terms.
- DFID – R4D website: <https://www.gov.uk/dfid-research-outputs> (40)
  - **Search note:** The R4D site includes detailed document type delimiters, including “systematic review.” This delimiter was selected, and the eight thematic search terms run, without including any additional study design terms.
    - **Rationale:** Selecting “systematic review” under “Document Type” was a stronger identifier than including “systematic review” in the search; *microfinance “systematic review”* returned over 1,000 results, while there are only 117 total documents classified as “systematic reviews” in the database using the “document type” identifier. The search function appeared to consider all search terms as “either/or” type searches, since, with “systematic review” document type selected, *microfinance meta-analysis* returned 31 results while *microfinance* returned only six; the additional results returned with the inclusion of the meta-analysis term were not related to microfinance. Thus, “systematic review” was selected as “document type” and only thematic searches were carried out.
- USAID Development Experience Clearinghouse: <https://dec.usaid.gov/dec/content/AdvancedSearch.aspx?ctID=ODVhZjk4NWQtM2YyMi00YjRmLTkxNjktZTcxMjM2NDBmY2Uy> (407)
  - **Search note:** Search results limited to “Document” type.

*Research institutions and research networks:*

- Center for Global Development: <https://www.cgdev.org/section/publications> (19)
  - **Search note:** Searches of publications on the CGD site are limited to title searches, thus, only thematic terms were searched, and results then screened for study design type.
- J-PAL: <https://www.povertyactionlab.org/evaluations> (258)
  - **Search note:** Searching all evaluations tagged as “Finance” with the built-in “Sector” identifier was more efficient than carrying out the 32 individual searches, each of which was returning documents that included the “Finance” identifier many times over. Thus, a single search of the “Finance” evaluations was undertaken, and results screened for thematic and study design relevance.
- 3ie: <http://www.3ieimpact.org/en/evidence/systematic-reviews/> (22)
  - **Search note:** The 3ie database has a detailed list of sectors within the Advanced Search option that includes “Microfinance.” Relevant studies were tagged with the “Microfinance” identifier even when the title used terms such as *microcredit*, and similarly, were included in the “Systematic Review” database even when the title referred to a *meta-analysis* or an *effectiveness review*. Thus, a single search that filtered all systematic reviews in the database to only show those with the “Microfinance” tag was undertaken, and results were screened for potential relevance.
- ELDIS: <https://www.eldis.org/search?sort=date_desc> (1,823)
  - **Search note:** Search limited to “document” type and published 2010-2018.
- SSRN: <https://papers.ssrn.com/sol3/DisplayAbstractSearch.cfm> (14)
  - **Search note:** Searches limited to Title; Abstract; or Keywords.
- ResearchGate: <https://www.researchgate.net/> (271)
- Academia.edu: [www.academia.edu](http://www.academia.edu) (505,136)
  - **Search note:** Due to the high numbers of results returned, which prohibited regular screening, results were screened up until all results viewable per page were irrelevant.

### Appendix 2 - MECIR checklist

Campbell Standards for reviews and their applicability to overviews of reviews. Note: this table is directly adapted from the Campbell MEC2IER standards and Table 1, Appendix S1 from Hartling, L., Chisholm, A., Thomson, D., & Dryden, D. (2012). A descriptive analysis of overviews of reviews published between 2000 and 2011. *PLOS One*, 7(11), e49667.

| Item No.* † | Item name | Standard | Applicability to overviews of reviews |
| --- | --- | --- | --- |
| Setting the research question(s) to inform the scope of the review | | | |
| 1 | Formulating review questions | Ensure that the review question and particularly the outcomes of interest, address issues that are important to stakeholders such as consumers, health professionals and policy makers. | Directly applicable |
| 2 | Pre-defining objectives | Define in advance the objectives of the review, including participants, interventions, comparators and outcomes. | Directly applicable |
| 3 | Considering potential adverse effects | Consider any important potential adverse effects of the intervention(s) and ensure that they are addressed. | Applicable. Overview authors should identify important outcomes including adverse effects and comment if any are not addressed or reported in the included SRs. If not addressed or reported in the SRs, overview authors need to decide whether to examine the primary studies to see if relevant outcomes were reported at the primary study level but not extracted at the SR level. |
| Setting eligibility criteria for including studies in the review | | | |
| 5 | Pre-defining unambiguous criteria for participants | Define in advance the eligibility criteria for participants in the studies. | Directly applicable |
| 7 | Pre-defining unambiguous criteria for interventions and comparators | Define in advance the eligible interventions and the interventions against which these can be compared in the included studies. | Directly applicable |
| 8 | Clarifying role of outcomes | Clarify in advance whether outcomes listed under ‘Criteria for considering studies for this review’ are used as criteria for including studies (rather than as a list of the outcomes of interest within whichever studies are included) | Directly applicable |
| 9 | Pre-defining study designs | Define in advance the eligibility criteria for study designs in a clear and unambiguous way, with a focus on features of a study’s design rather than design labels. | Directly applicable; need to define what is considered a SR. |
| 12 | Excluding studies based on publication status | Include studies irrespective of their publication status, unless explicitly justified. | Directly applicable |
| 13 | Changing eligibility criteria | Justify any changes to eligibility criteria or outcomes studied. In particular, post hoc decisions about inclusion or exclusion of studies should keep faith with the objectives of the review rather than with arbitrary rules. | Directly applicable |
| 14 | Pre-defining outcomes | Define in advance which outcomes are primary outcomes and which are secondary outcomes. | Directly applicable |
| Planning the review methods at protocol stage | | | |
| 19 | Planning the search | Plan in advance the methods to be used for identifying studies. Design searches to capture as many studies as possible meeting the eligibility criteria, ensuring that relevant time periods and sources are covered and not restricting by language or publication status. | Directly applicable |
| 20 | Planning the assessment of risk of bias in included studies | Plan in advance the methods to be used for assessing risk of bias in included studies, including the tool(s) to be used, how the tool(s) will be implemented, and the criteria used to assign studies, for example, to judgements of low risk, high risk and unclear risk of bias. | Applicable. Overview authors should determine whether they will extract risk of bias assessments from the included SRs or conduct risk of bias assessments on the primary studies themselves. Overview authors should determine how they will handle discrepancies in approaches to risk of bias assessments across SRs. Overview authors should determine whether and how they will assess methodological quality of the included SRs. |
| 21 | Planning the synthesis of results | Plan in advance the methods to be used to synthesize the results of the included studies, including whether a quantitative synthesis is planned, how heterogeneity will be assessed, choice of effect measure (e.g. odds ratio, risk ratio, risk difference or other for dichotomous outcomes), and methods for meta-analysis (e.g. inverse variance or Mantel Haenszel, fixed-effect or random effects model). | Applicable. Overview authors should determine how they will present the data from included SRs and whether they will re-analyze data to provide consistency (e.g., choice of effect measure, method of analysis). |
| 22 | Planning subgroup analyses | Pre-define potential effect modifiers (e.g. for subgroup analyses) at the protocol stage; restrict these in number; and provide rationale for each. | Applicable. Overview authors should specify subgroups of interest and determine whether they will conduct additional analyses if subgroups of interest are not examined or reported in the included SRs. |
| Searching for studies | | | |
| 24 | Searching key databases | Search the Cochrane Review Group's Specialized Register (internally, e.g. via the Cochrane Register of Studies, or externally via CENTRAL). Ensure that CENTRAL and MEDLINE (e.g. via PubMed) have been searched (either for the review or for the Review Group’s Specialized Register). | Applicable. Overview authors should search The Cochrane Library (i.e., Cochrane Database of Systematic Reviews and Database of Abstracts of Reviews of Effectiveness) and may wish to consult relevant Cochrane Review Groups for a listing of reviews. |
| 32 | Structuring search strategies for bibliographic databases | Inform the structure of search strategies in bibliographic databases around the main concepts of the review, using appropriate elements from PICO and study design. In structuring the search, maximize sensitivity whilst striving for reasonable precision. Ensure correct use of the AND and OR operators. | Directly applicable |
| 33 | Developing search strategies for bibliographic databases | Identify appropriate controlled vocabulary (e.g. MeSH, Emtree, including 'exploded' terms) and free-text terms (considering, for example, spelling variants, synonyms, acronyms, truncation and proximity operators). | Directly applicable |
| 35 | Restricting database searches | Justify the use of any restrictions in the search strategy on publication date, publication format or language. | Directly applicable |
| 36 | Documenting the search process | Document the search process in enough detail to ensure that it can be reported correctly in the review. | Directly applicable |
| 37 | Rerunning searches | Rerun or update searches for all relevant databases within 12 months before publication of the review or review update, and screen the results for potentially eligible studies. | Directly applicable |
| Selecting studies into the review | | | |
| 39 | Making inclusion decisions | Use (at least) two people working independently to determine whether each study meets the eligibility criteria, and define in advance the process for resolving disagreements. | Directly applicable |
| 40 | Excluding studies without useable data | Include studies in the review irrespective of whether measured outcome data are reported in a ‘usable’ way. | Directly applicable |
| 41 | Documenting decisions about records identified | Document the selection process in sufficient detail to complete a PRISMA flow chart and a table of ‘Characteristics of excluded studies’. | Directly applicable |
| 42 | Collating multiple reports | Collate multiple reports of the same study, so that each study rather than each report is the unit of interest in the review. | Directly applicable (e.g., SR published in Cochrane Library and peer-reviewed journal; published and unpublished version of he same SR). |
| Collecting data from included studies | | | |
| 43 | Using data collection forms | Use a data collection form, which has been piloted. | Directly applicable |
| 44 | Describing studies | Collect characteristics of the included studies in sufficient detail to populate a table of ‘Characteristics of included studies’. | Directly applicable |
| 46 | Extracting outcome data in duplicate | Use (at least) two people working independently to extract outcome data from reports of each study, and define in advance the process for resolving disagreements. | Directly applicable |
| 47 | Making maximal use of data | Collect and utilize the most detailed numerical data that might facilitate similar analyses of included studies. | Applicable. Overview authors should extract detailed data from meta-analyses when available that will facilitate comparisons across SRs. |
| 50 | Choosing intervention groups in multiarm studies | If a study is included with more than two intervention arms, include in the review only intervention and control groups that meet the eligibility criteria. | Overview authors should be aware of how SR authors have handled such studies. |
| 51 | Checking accuracy of numeric data in the review | Compare magnitude and direction of effects reported by studies with how they are presented in the review, taking account of legitimate differences. | Applicable. Caution is needed when comparing interventions that have not been formally compared in either direct or indirect analyses. |
| Assessing risk of bias in included studies | | | |
| 52 | Assessing risk of bias | Assess the risk of bias for each included study. | Determine a priori whether overview authors will assess the methodological quality of included SRs and what tool will be used. |
| 53 | Assessing risk of bias in duplicate | Use (at least) two people working independently to apply the risk of bias tool to each included study, and define in advance the process for resolving disagreements. | Applicable based on assessing methodological quality of SRs. |
| 54 | Supporting  judgements of risk of bias | Justify judgements of risk of bias (high, low and unclear) and provide this information in the ‘Risk of bias’ tables (as ‘Support for judgement’). | Applicable based on assessing methodological quality of SRs. |
| 61 | Incorporating assessments of risk of bias | If randomized trials have been assessed using one or more tools in addition to the Cochrane ‘Risk of bias’ tool, use the Cochrane tool as the primary assessment of bias for interpreting results, choosing the primary analysis, and drawing conclusions. | Applicable to extracting and reporting risk of bias assessments for individual studies that were included in the included SRs. |
| Summarizing the findings | | | |
| 76 | Assessing the quality of the body of evidence | Use the five GRADE considerations (study limitations, consistency of effect, imprecision, indirectness and publication bias) to assess the quality of the body of evidence for each outcome, and to draw conclusions about the quality of evidence within the text of the review. | Extract quality of evidence assessments from the included SRs. Decide a priori what to do if quality of evidence assessments have not been performed or performed inconsistently across SRs. |
| 77 | Justifying assessments of the quality of the body of evidence | Justify and document all assessments of the quality of the body of evidence (for example downgrading or upgrading if using the GRADE tool). | Extract relevant information from the SRs. |
| Reaching conclusions | | | |
| 78 | Formulating implications for practice | Base conclusions only on findings from the synthesis (quantitative or narrative) of studies included in the review. | Directly applicable |
| 79 | Avoiding recommendations | Avoid providing recommendations for practice. | Directly applicable |

* The items listed are among those considered mandatory for Cochrane Intervention Reviews. The item numbers, names, and standards are from: Chandler J, Churchill R, Higgins J, Lasserson T, Tovey D. Methodological standards for the conduct of new Cochrane Intervention Reviews. Version 2.1, 8 December 2011.

† The section from the above citation on ‘Synthesizing the results of included studies’ has been omitted from this table as it relates to the quantitative synthesis of individual studies in a meta-analysis. For the most part, overviews of reviews have been descriptive in nature. Guidance on performing indirect analyses or mixed treatment comparisons is beyond the scope of this paper.

### Appendix 3 – List of included meta-studies and their main research question

**11 medium- and high-confidence meta-studies:**

| **Authors** | **Year** | **Main research question** |
| --- | --- | --- |
|  |  |  |
| Steinert et al. | 2018 | What is the evidence on the effectiveness of saving promotion in Sub-Saharan Africa? |
| Vaessen et al. | 2014 | What does the impact evaluative evidence say about the causal relationship between microcredit and specific dimensions of women’s empowerment (women’s control over household spending)? |
| Brody et al. | 2015 | What is the impact of women’s economic self-help groups on women’s individual empowerment in low and middle-income countries? |
| Stewart et al. | 2012 | Do micro-credit, micro-savings and micro-leasing serve as effective financial inclusion interventions enabling poor people, and especially women, to engage in meaningful economic opportunities in LMICs? |
| Duvendack et al. | 2011 | What is the evidence of the impact of microfinance on the well-being of poor people? |
| Orton et al. | 2016 | What impact do group-based microfinance schemes based on collective empowerment have on health? What is the role of empowerment? Do the impacts differ based on the ethnicity, sex and/or socioeconomic status of the members? |
| Gopalaswamy et al. | 2016 | What is the impact of microfinance on the well-being of the poor and what are the conditions for making microfinance work for the poor in South Asia? |
| Peters et al. | 2016 | What are the perceived or apparent benefits/negative consequences of participating in a microfinance programme? |
| Stewart et al. | 2010 | What studies have been done in SSA on the impact of microfinance on poor people? |
| Chliova et al. | 2015 | How does micro-credit affect entrepreneurial and other key development outcomes at the individual level of the client? |
| Kennedy et al. | 2014 | How effective are income generation interventions in improving HIV outcomes? |

**21 low-confidence meta-studies:**

| **Authors** | **Year** | **Main research question** |
| --- | --- | --- |
|  |  |  |
| Habib et al. | 2016 | What is the extent to which MHI has contributed to providing financial risk protection to low-income households in developing countries? |
| Lorenzetti et al. | 2017 | What is the effect of integrated microfinance and health programs? |
| Cole et al. | 2012 | What is the effectiveness of index-based insurance in helping the developing country poor manage weather-related risk? |
| Maîtrot & Niño-Zarazúa | 2017 | Does access to credit leads to poverty reduction and improved wellbeing? |
| Pande et al. | 2012 | Can formal banking services raise the incomes of the poor? |
| Apostolakis et al. | 2015 | What, how, where and for whom is microinsurance performance measured? |
| Arrivillaga & Salcedo | 2014 | What is the scope of microfinance-based interventions for HIV/AIDS prevention? |
| Bhageerathy et al. | 2017 | What are the factors affecting the take up of voluntary and community-based health insurance programs? |
| Awaworyi Churchill et al. | 2016 | Whether or not the impact of microcredit on poverty in Bangladesh is truly positive. |
| Awaworyi Churchill | 2015 | What is the impact of microfinance on five measures of female empowerment used in the empirical literature, namely mobility, decision-making power, control over finance, awareness and women's assets? |
| Madhani et al. | 2015 | What is the association between participation in a micro-finance programme and women’s mental health outcomes, specifically (a) psychosocial functioning; (b) emotional stress; and (c) prevalence of IPV in South Asia? |
| Marr et al. | 2016 | What are the determinants of demand for index-insurance, the impact of index-insurance on smallholder livelihoods, and the existing links between index-insurance and credit? |
| O'Malley & Burke | 2017 | Is microfinance an effective approach for improved women’s health? |
| Awaworyi Churchill | 2014 | What is the impact of microcredit and access to microcredit on poverty and on microenterprises? |
| Gammage et al. | 2017 | Where and how does gender influence financial inclusion and digital financial inclusion? |
| Gash | 2017 | What do we now know about the impact of SGs? |
| Hidalgo | 2009 | Why is there different results in the evidence of micro-credit? |
| Isangula | 2012 | How can rural women, children and family’s health be improved through integrating income generation and health education & promotion activities for women? |
| O'Grady | 2016 | Can microfinance alleviate poverty? |
| Palmkvist & Lin | 2015 | What is the evidence on the effects of microfinance self-help groups on women’s empowerment? What are the mechanisms that influence the process of empowerment? |
| Yang & Stanley | 2013 | Whether or not there have been any positive effects on income from micro-credit and business education classes. |

### Appendix 4 – Number and proportion of meta-studies by PICOS characteristics

**11 medium- and high-confidence meta-studies:**

**21 low-confidence meta-studies:**

### Appendix 5 – List of excluded studies

| No | Author | Year | Title | Full text screening | Reasons for exclusion |
| --- | --- | --- | --- | --- | --- |
| 1 | van Rooyen, C., Stewart, R. & de Wet, T. | 2012 | The Impact of Microfinance in Sub-Saharan Africa: A Systematic Review of the Evidence | No | Duplicate identified after subsequent title/abstract or full text screening |
| 2 | Habib, S. S., Perveen, S. & Khuwaja, H. M. A. | 2016 | The role of micro health insurance in providing financial risk protection in developing countries- a systematic review | Yes | Duplicate identified after subsequent title/abstract or full text screening |
| 3 | Korth, M., Stewart, R., Van Rooyen, C. & De Wet, T. | 2012 | Microfinance: Development Intervention or Just Another Bank? | Yes | Duplicate identified after subsequent title/abstract or full text screening |
| 4 | Marr, A., Winkel, A., van Asseldonk, M., Lensink, R. & Bulte, E. | 2016 | Adoption and impact of index-insurance and credit for smallholder farmers in developing countries | Yes | Duplicate identified after subsequent title/abstract or full text screening |
| 5 | Korth, M., Stewart, R., Van Rooyen, C. & De Wet, T. | 2012 | Microfinance: Development Intervention or Just Another Bank? | Yes | Duplicate identified after subsequent title/abstract or full text screening |
| 6 | Marr, A., Winkel, A., van Asseldonk, M., Lensink, R. & Bulte, E. | 2016 | Adoption and impact of index-insurance and credit for smallholder farmers in developing countries | Yes | Duplicate identified after subsequent title/abstract or full text screening |
| 7 | Madhani, F. I., Tompkins, C., Jack, S. M. & Fisher, A. | 2015 | Participation in Micro-Finance Programmes and Women's Mental Health in South Asia: A Modified Systematic Review | No | Duplicate identified after subsequent title/abstract or full text screening |
| 8 | Arrivillaga, M. & Salcedo, J. P. | 2014 | A SYSTEMATIC REVIEW OF MICROFINANCE-BASED INTERVENTIONS FOR HIV/AIDS PREVENTION | No | Duplicate identified after subsequent title/abstract or full text screening |
| 9 | Brody, C., de Hoop, T., Vojtkova, M., Warnock, R., Dunbar, M., Murthy, P. & Dworkin, S. L. | 2015 | Economic Self-Help Group Programs for Improving Women’s Empowerment: A Systematic Review | No | Duplicate identified after subsequent title/abstract or full text screening |
| 10 | Brody, C., de Hoop, T., Vojtkova, M., Warnock, R., Dunbar, M., Murthy, P. & Dworkin, S. L. | 2015 | Economic self-help group programmes for improving women’s empowerment: A systematic review, 3ie Systematic Review 23 | No | Duplicate identified after subsequent title/abstract or full text screening |
| 11 | Vaessen, J., Rivas, A., Duvendack, M., Palmer-Jones, R., Leeuw, F., van Gils, G., Lukach, R., Holvoet, N., Bastiaensen, J., Hombrados, J. G. & Waddington, H. | 2014 | The Effects of Microcredit on Women’s Control over Household Spending in Developing Countries: A Systematic Review and Meta-analysis | No | Duplicate identified after subsequent title/abstract or full text screening |
| 12 | van Rooyen, C., Stewart, R. & de Wet, T. | 2012 | The Impact of Microfinance in Sub-Saharan Africa: A Systematic Review of the Evidence | No | Duplicate identified after subsequent title/abstract or full text screening |
| 13 | Awaworyi Churchill, S., Korankye Danso, J. & Appau, S. | 2015 | Microcredit and Poverty Reduction in Bangladesh: Beyond Publication Bias, Does Genuine Effect Exist? | Yes | Duplicate identified after subsequent title/abstract or full text screening |
| 14 | Duvendack, M., Palmer-Jones, R. & Vaessen, J. | 2014 | Meta-analysis of the impact of microcredit on women's control over household decisions: methodological issues and substantive findings | No | Duplicate identified after subsequent title/abstract or full text screening |
| 15 | Chliova, M., Brinckmann, J. & Rosenbusch, N. | 2013 | IS MICROCREDIT A BLESSING FOR THE POOR? A METAANALYSIS | Yes | Duplicate identified after subsequent title/abstract or full text screening |
| 16 | Chliova, M., Brinckmann, J. & Rosenbusch, N. | 2014 | Is microcredit a blessing for the poor? A meta-analysis examining development outcomes and contextual considerations | Yes | Duplicate identified after subsequent title/abstract or full text screening |
| 17 | Cho, Y. & Honorati, M. | 2013 | Entrepreneurship programs in developing countries: a meta regression analysis | Yes | Excluded because of intervention inclusion/exclusion criterion |
| 18 | Gibbs, A., Willan, S., Misselhorn, A. & Mangoma, J. | 2012 | Combined structural interventions for gender equality and livelihood security: a critical review of the evidence from southern and eastern Africa and the implications for young people | Yes | Excluded because of intervention inclusion/exclusion criterion |
| 19 | Giedion, U. & Díaz, B. Y. | 2010 | A review of the evidence | Yes | Excluded because of intervention inclusion/exclusion criterion |
| 20 | Korth, M., Stewart, R., Van Rooyen, C. & De Wet, T. | 2012 | Microfinance: Development Intervention or Just Another Bank? | Yes | Excluded because of intervention inclusion/exclusion criterion |
| 21 | Bouillon, C. P. & Tejerina, L. | 2006 | DO WE KNOW WHAT WORKS? A Systematic Review of Impact Evaluations of Social Programs in Latin America and the Caribbean | Yes | Excluded because of intervention inclusion/exclusion criterion |
| 22 | Magnoni, B. & Zimmerman, E. | 2011 | Do clients get value from microinsurance? A systematic review of recent and current research | Yes | Excluded because of intervention inclusion/exclusion criterion |
| 23 | Sulaiman, M. | 2016a | Making Sustainable Reductions in Extreme Poverty: A Comparative Meta-Analysis of Livelihood, Cash Transfer and Graduation Approaches | Yes | Excluded because of intervention inclusion/exclusion criterion |
| 24 | Atan, N. A. B. & Johari, F. B. | 2017 | A review on literature of Waqf for poverty alleviation between 2006-2016 | No | Excluded because of intervention inclusion/exclusion criterion |
| 25 | Bassani, D. G., Arora, P., Wazny, K., Gaffey, M. F., Lenters, L. & Bhutta, Z. A. | 2013 | Financial incentives and coverage of child health interventions: A systematic review and meta-analysis | No | Excluded because of intervention inclusion/exclusion criterion |
| 26 | Bateganya, M. H., Dong, M. X., Oguntomilade, J. & Suraratdecha, C. | 2015 | The Impact of Social Services Interventions in Developing Countries: A Review of the Evidence of Impact on Clinical Outcomes in People Living With HIV | No | Excluded because of intervention inclusion/exclusion criterion |
| 27 | Boehe, D. M. & Cruz, L. B. | 2013 | Gender and Microfinance Performance: Why Does the Institutional Context Matter? | No | Excluded because of intervention inclusion/exclusion criterion |
| 28 | Cooney, K. & Shanks, T. R. W. | 2010 | New Approaches to Old Problems: Market-Based Strategies for Poverty Alleviation | No | Excluded because of intervention inclusion/exclusion criterion |
| 29 | Ellis, C. M. & Chaffin, J. | 2015 | Evaluations of outcomes for children and youth from NGO-supported microeconomic interventions: A research synthesis | No | Excluded because of intervention inclusion/exclusion criterion |
| 30 | Engelken, M., Romer, B., Drescher, M., Welpe, I. M. & Picot, A. | 2016 | Comparing drivers, barriers, and opportunities of business models for renewable energies: A review | No | Excluded because of intervention inclusion/exclusion criterion |
| 31 | Halim, N., Spielman, K. & Larson, B. | 2015 | The economic consequences of selected maternal and early childhood nutrition interventions in low- and middle-income countries: a review of the literature, 2000--2013 | No | Excluded because of intervention inclusion/exclusion criterion |
| 32 | Jacinta, N. | 2014 | Interest Rates, Target Markets and Sustainability in Microfinance | No | Excluded because of intervention inclusion/exclusion criterion |
| 33 | Kabeer, N. & Waddington, W. | 2015 | Economic impacts of conditional cash transfer programmes: a systematic review and meta-analysis | No | Excluded because of intervention inclusion/exclusion criterion |
| 34 | Kysucky, V. & Norden, L. | 2016 | The Benefits of Relationship Lending in a Cross-Country Context: A Meta-analysis | No | Excluded because of intervention inclusion/exclusion criterion |
| 35 | Nwolise, C. H., Hussein, J., Kanguru, L., Bell, J. & Patel, P. | 2015 | The Effectiveness of Community-Based Loan Funds for Transport during Obstetric Emergencies in Developing Countries: A Systematic Review | No | Excluded because of intervention inclusion/exclusion criterion |
| 36 | Rathore, B. S. | 2015 | Social capital: Does it matter in a microfinance contract? | No | Excluded because of intervention inclusion/exclusion criterion |
| 37 | Sondergaard, L., Murthi, M., Abu-Ghaida, D., Bodewig, C. &. Rutkowski, J. | 2011 | Overview | No | Excluded because of intervention inclusion/exclusion criterion |
| 38 | Thow, A. M., Fanzo, J. & Negin, J. | 2016 | A Systematic Review of the Effect of Remittances on Diet and Nutrition | No | Excluded because of intervention inclusion/exclusion criterion |
| 39 | Caton, C., Chaffin, J., Marsh, M. & Read-Hamilton, S. | 2014 | Empowered and Safe: Economic Strengthening for Girls in Emergencies | No | Excluded because of intervention inclusion/exclusion criterion |
| 40 | Reichert, P. | 2016 | A meta-analysis examining the nature of trade-offs in microfinance | No | Excluded because of intervention inclusion/exclusion criterion |
| 41 | Tripney, J., Roulstone, A., Vigurs, C., Hogrebe, N., Schmidt, E. & Stewart, R. | 2015 | Interventions to Improve the Labour Market Situation of Adults with Physical and/or Sensory Disabilities in Low- and Middle-Income Countries: A Systematic Review | No | Excluded because of intervention inclusion/exclusion criterion |
| 42 | Wulandaria, P. & Kassim, S. H. | 2015 | Can Islamic Microfinance Provide Solutions to Financial Constraint Issues in Reaching the Millennium Development Goals? | No | Excluded because of intervention inclusion/exclusion criterion |
| 43 | Yoong, J., Rabinovich, L. & Diepeveen, S. | 2012 | The impact of economic resource transfers to women versus men: A systematic review | No | Excluded because of intervention inclusion/exclusion criterion |
| 44 | Cho, Y. & Honorati, M. | 2014 | Entrepreneurship programs in developing countries: A meta regression analysis | Yes | Excluded because of intervention inclusion/exclusion criterion, duplicate |
| 45 | Cho, Y. & Honorati, M. | 2013 | Entrepreneurship Programs in Developing Countries: A Meta Regression Analysis | Yes | Excluded because of intervention inclusion/exclusion criterion, duplicate |
| 46 | Cho, Y. & Honorati, M. | 2013 | Entrepreneurship Programs in Developing Countries: A Meta Regression Analysis | Yes | Excluded because of intervention inclusion/exclusion criterion, duplicate |
| 47 | Sulaiman, M., Goldberg, N., Karlan, D. & de Montesquiou, A. | 2016b | Eliminating Extreme Poverty: Comparing the Cost-Effectiveness of Livelihood, Cash Transfer, and Graduation Approaches | Yes | Excluded because of intervention inclusion/exclusion criterion, duplicate |
| 48 | Bassani, D. G., Paul, A., Wazny, K., Gaffey, M. F., Lenters, L. & Zulfiqar, A. B. | 2013 | Financial incentives and coverage of child health interventions: a systematic review and meta-analysis | No | Excluded because of intervention inclusion/exclusion criterion, duplicate |
| 49 | Engelken, M., Römer, B., Drescher, M., Welpe, I. M. & Picot, A. | 2016 | Comparing drivers, barriers, and opportunities of business models for renewable energies: A review | No | Excluded because of intervention inclusion/exclusion criterion, duplicate |
| 50 | Halim, N., Spielman, K. & Larson, B. | 2015 | The economic consequences of selected maternal and early childhood nutrition interventions in low- and middle-income countries: A review of the literature, 2000-2013 | No | Excluded because of intervention inclusion/exclusion criterion, duplicate |
| 51 | Rathore, B. S. | 2015 | Social capital: does it matter in a microfinance contract? | No | Excluded because of intervention inclusion/exclusion criterion, duplicate |
| 52 | Jennings, L. | 2014 | Do Men Need Empowering Too? A Systematic Review of Entrepreneurial Education and Microenterprise Development on Health Disparities among Inner-City Black Male Youth | No | Excluded because of population inclusion/exclusion criterion |
| 53 | Ahamad, S., Bakar, R. & Lubis, Z. | 2016 | Islamic Microfinance and Its Impacts on Borrowers: A Systematic Review From 1995-2015 | Yes | Excluded because of study design inclusion/exclusion criterion |
| 54 | Akter, S. | 2012 | The Role of Microinsurance as a Safety Net against Environmental Risks in Bangladesh | No | Excluded because of study design inclusion/exclusion criterion |
| 55 | Assensoh-Kodua, A., Migiro, S. & Mutambara, E. | 2016 | Mobile Banking in South Africa: A Systematic Review of the Literature | No | Excluded because of study design inclusion/exclusion criterion |
| 56 | Azimi, H. | 2013 | Role of bank credits in development of agriculture sector | No | Excluded because of study design inclusion/exclusion criterion |
| 57 | Boccia, D., Hargreaves, J., Lönnroth, K., Jaramillo, E., Weiss, J., Uplekar, M., Porter, J. D. H. & Evans, C. A. | 2011 | Cash transfer and microfinance interventions for tuberculosis control: Review of the impact evidence and policy implications | No | Excluded because of study design inclusion/exclusion criterion |
| 58 | Dhanalakshmi, U. & Rajini, K. | 2013 | A Review of the Literature: Women Empowerment through Self Help Groups (SHGs) | No | Excluded because of study design inclusion/exclusion criterion |
| 59 | Garcia-Perez, I., Munoz-Torres, M. J. & Fernandez-Izquierdo, M. A. | 2017 | Microfinance literature: A sustainability level perspective survey | No | Excluded because of study design inclusion/exclusion criterion |
| 60 | Lahimer, N., Dash, S. & Zaiter, M. | 2013 | Does microfinance promote entrepreneurship and innovation? A macro analysis | No | Excluded because of study design inclusion/exclusion criterion |
| 61 | Casselman, R. M., Cocozzelli, F. P. & Sama, L. M. | 2014 | The Role of Microfinance Institutions in Post-conflict Settings | No | Excluded because of study design inclusion/exclusion criterion |
| 62 | Dass, R. & Pal, S. | 2011 | A Meta Analysis on Adoption of Mobile Financial Services | No | Excluded because of study design inclusion/exclusion criterion |
| 63 | Meager, R. | 2015 | Understanding the Impact of Microcredit Expansions: A Bayesian Hierarchical Analysis of 7 Randomised Experiments | No | Excluded because of study design inclusion/exclusion criterion |
| 64 | Nwachukwu, J. | 2014 | Interest Rates, Target Markets and Sustainability in Microfinance | No | Excluded because of study design inclusion/exclusion criterion, duplicate |

### Appendix 6 – Correlation matrix of low confidence meta-studies to demonstrate overlap

|  | **Pande et al. 2012** | **Yang et al. 2013** | **Awaworyi et al. 2015** | **Arrivillaga et al. 2014** | **Awaworyi et al. 2016** | **Habib et al. 2016** | **Marr et al. 2016** | **O'Grady, 2015** | **Bhageerathy et al. 2017** | **Gash, 2017** | **O’Malley et al. 2017** | **Cole et al. 2012** | **Hidalgo, 2009** | **Apostolakis et al. 2015** | **Maîtrot et al. 2017** | **Madhani et al. 2015** | **Palmkvist et al. 2015** | **Isangula, 2012** | **Lorenzetti et al. 2017** | **Awaworyi et al. 2014** |
| --- | --- | --- | --- | --- | --- | --- | --- | --- | --- | --- | --- | --- | --- | --- | --- | --- | --- | --- | --- | --- |
| **Pande et al. 2012** | 100% | 0% | 0% | 0% | 0% | 0% | 0% | 6% | 0% | 0% | 0% | 0% | 0% | 0% | 0% | 0% | 0% | 0% | 0% | 0% |
| **Yang et al. 2013** | 0% | 100% | 0% | 0% | 0% | 0% | 0% | 0% | 0% | 0% | 0% | 0% | 0% | 0% | 10% | 0% | 0% | 0% | 0% | 21% |
| **Awaworyi et al. 2015** | 0% | 0% | 100% | 0% | 33% | 0% | 0% | 0% | 0% | 0% | 0% | 0% | 7% | 0% | 6% | 0% | 8% | 0% | 0% | 21% |
| **Arrivillaga et al. 2014** | 0% | 0% | 0% | 100% | 0% | 0% | 0% | 0% | 0% | 0% | 10% | 0% | 0% | 0% | 0% | 0% | 8% | 6% | 17% | 0% |
| **Awaworyi et al. 2016** | 0% | 0% | 29% | 0% | 100% | 0% | 0% | 0% | 0% | 0% | 0% | 0% | 3% | 0% | 8% | 0% | 0% | 0% | 0% | 21% |
| **Habib et al. 2016** | 0% | 0% | 0% | 0% | 0% | 100% | 0% | 0% | 8% | 0% | 0% | 0% | 0% | 1% | 0% | 0% | 0% | 0% | 0% | 0% |
| **Marr et al. 2016** | 0% | 0% | 0% | 0% | 0% | 0% | 100% | 0% | 0% | 0% | 0% | 15% | 0% | 0% | 0% | 0% | 0% | 0% | 0% | 0% |
| **O'Grady, 2015** | 6% | 0% | 0% | 0% | 0% | 0% | 0% | 100% | 0% | 0% | 0% | 8% | 0% | 0% | 6% | 0% | 0% | 0% | 6% | 4% |
| **Bhageerathy et al. 2017** | 0% | 0% | 0% | 0% | 0% | 5% | 0% | 0% | 100% | 0% | 2% | 0% | 0% | 1% | 0% | 0% | 0% | 0% | 0% | 0% |
| **Gash, 2017** | 0% | 0% | 0% | 0% | 0% | 0% | 0% | 0% | 0% | 100% | 0% | 0% | 0% | 0% | 0% | 0% | 0% | 0% | 0% | 0% |
| **O’Malley et al. 2017** | 0% | 0% | 0% | 29% | 0% | 0% | 0% | 0% | 8% | 0% | 100% | 0% | 3% | 0% | 2% | 8% | 8% | 8% | 40% | 0% |
| **Cole et al. 2012** | 0% | 0% | 0% | 0% | 0% | 0% | 6% | 6% | 0% | 0% | 0% | 100% | 0% | 0% | 0% | 0% | 0% | 0% | 0% | 0% |
| **Hidalgo, 2009** | 0% | 0% | 29% | 0% | 17% | 0% | 0% | 0% | 0% | 0% | 2% | 0% | 100% | 0% | 8% | 0% | 0% | 0% | 0% | 17% |
| **Apostolakis et al. 2015** | 0% | 0% | 0% | 0% | 0% | 5% | 0% | 0% | 8% | 0% | 0% | 0% | 0% | 100% | 2% | 0% | 0% | 2% | 0% | 0% |
| **Maîtrot et al. 2017** | 0% | 83% | 43% | 0% | 67% | 0% | 0% | 19% | 0% | 0% | 2% | 0% | 14% | 1% | 100% | 0% | 0% | 2% | 9% | 33% |
| **Madhani et al. 2015** | 0% | 0% | 0% | 0% | 0% | 0% | 0% | 0% | 0% | 0% | 2% | 0% | 0% | 0% | 0% | 100% | 17% | 2% | 0% | 0% |
| **Palmkvist et al. 2015** | 0% | 0% | 14% | 7% | 0% | 0% | 0% | 0% | 0% | 0% | 2% | 0% | 0% | 0% | 0% | 17% | 100% | 2% | 0% | 4% |
| **Isangula, 2012** | 0% | 0% | 0% | 21% | 0% | 0% | 0% | 0% | 0% | 0% | 10% | 0% | 0% | 1% | 2% | 8% | 8% | 100% | 9% | 4% |
| **Lorenzetti et al. 2017** | 0% | 0% | 0% | 43% | 0% | 0% | 0% | 13% | 0% | 0% | 34% | 0% | 0% | 0% | 6% | 0% | 0% | 6% | 100% | 0% |
| **Awaworyi et al. 2014** | 0% | 83% | 71% | 0% | 83% | 0% | 0% | 6% | 0% | 0% | 0% | 0% | 14% | 0% | 16% | 0% | 8% | 2% | 0% | 100% |

Note: Gammage et al. (2017) is excluded from this table of low confidence meta-studies as it claims to have synthesized 594 studies but the reference list does not provide a full list of all 594 studies and hence we could not assess overlap of Gammage et al. (2017) with all other included meta-studies.

### Appendix 7 - AMSTAR2 and 3ie critical appraisal checklist

***Table A 7.1: AMSTAR2***

| **1.** | **Did the research questions and inclusion criteria for the review include the components of PICO?** | | | | | | |
| --- | --- | --- | --- | --- | --- | --- | --- |
|  |  |  |  |  |  |  |  |
| For Yes: |  |  |  |  | Optional (recommended) |  |  |
|  | Population | | | | Timeframe for follow-up |  | Yes |
|  |  |  |  |  |  |  |  |
|  | Intervention | | | |  |  | No |
|  |  |  |  |  |  |  |  |
|  | Comparator group | | | |  |  |  |
|  |  | |  |  |  |  |  |
|  | Outcome | | | |  |  |  |
|  |  |  |  |  |  |  |  |

**2. Did the report of the review contain an explicit statement that the review methods were established prior to the conduct of the review and did the report justify any significant deviations from the protocol?**

| For Partial Yes: | | For Yes: |  |  |  |  |  |
| --- | --- | --- | --- | --- | --- | --- | --- |
| The authors state that they had a written | | As for partial yes, plus the protocol | | | |  |  |
| protocol or guide that included ALL the | | should be registered and should also | | | |  |  |
| following: | | have specified: | | | |  |  |
|  |  |  |  |  |  |  | Yes |
|  | review question(s) |  | a meta-analysis/synthesis plan, | | |  | Partial Yes |
|  | a search strategy |  | if appropriate, *and* | | |  | No |
|  | inclusion/exclusion criteria | a plan for investigating causes | | | |  |  |
|  | a risk of bias assessment |  | of heterogeneity | | |  |  |
|  |  | justification for any deviations | | | |  |  |
|  |  |  |  |  |  |  |  |
|  |  |  | from the protocol | | |  |  |
| **3.** | **Did the review authors explain their selection of the study designs for inclusion in the review?** | | | | | | |
|  | | | |  |  |  |  |
| For Yes, the review should satisfy ONE of the following: | | | | | |  |  |
|  | *Explanation for* including only RCTs | |  |  |  |  | Yes |
|  | OR *Explanation for* including only NRSI | |  |  |  |  | No |
|  | OR *Explanation for* including both RCTs and NRSI | | | | |  |  |
| **4.** | **Did the review authors use a comprehensive literature search strategy?** | | | | |  |  |
|  | |  | |  |  |  |  |
| For Partial Yes (all the following): | | For Yes, should also have (all the | | | |  |  |
|  |  | following): | | | |  |  |
|  | searched at least 2 databases |  | searched the reference lists / | | |  | Yes |
|  | (relevant to research question) |  | bibliographies of included | | |  | Partial Yes |
|  | provided key word and/or |  | studies | | |  | No |
|  | search strategy |  | searched trial/study registries | | |  |  |
|  | justified publication restrictions |  | included/consulted content | | |  |  |
|  | (e.g. language) |  | experts in the field | | |  |  |
|  |  | where relevant, searched for | | | |  |  |
|  |  |  | grey literature | | |  |  |
|  |  | conducted search within 24 | | | |  |  |
|  |  |  | months of completion of the | | |  |  |
|  |  |  | review | | |  |  |
| **5.** | **Did the review authors perform study selection in duplicate?** | | | | |  |  |
|  | |  |  |  |  |  |  |
| For Yes, either ONE of the following: | |  |  |  |  |  |  |
|  | at least two reviewers independently agreed on selection of eligible studies | | | | |  | Yes |
|  | and achieved consensus on which studies to include | | | | |  | No |
|  | OR two reviewers selected a sample of eligible studies and achieved good | | | | |  |  |
|  |  |  |  |  |  |  |  |

agreement (at least 80 percent), with the remainder selected by one reviewer.

1. **Did the review authors perform data extraction in duplicate?**

| For Yes, either ONE of the following: | |  |  |  |  |  |  |
| --- | --- | --- | --- | --- | --- | --- | --- |
|  | at least two reviewers achieved consensus on which data to extract from | | | | |  | Yes |
|  | included studies |  |  |  |  |  | No |
|  | OR two reviewers extracted data from a sample of eligible studies and | | | | |  |  |
|  | achieved good agreement (at least 80 percent), with the remainder | | |  |  |  |  |
|  | extracted by one reviewer. |  |  |  |  |  |  |
| **7.** | **Did the review authors provide a list of excluded studies and justify the exclusions?** | | | | | | |
|  | |  | | | |  |  |
| For Partial Yes: | | For Yes, must also have: | | | |  |  |
|  | provided a list of all potentially | Justified the exclusion from | | | |  | Yes |
|  | relevant studies that were read |  | the review of each potentially | | |  | Partial Yes |
|  | in full-text form but excluded |  | relevant study | | |  | No |
|  | from the review |  |  |  |  |  |  |
| **8.** | **Did the review authors describe the included studies in adequate detail?** | | | | |  |  |
|  | |  | | | |  |  |
| For Partial Yes (ALL the following): | | For Yes, should also have ALL the | | | |  |  |
|  |  | following: | | | |  |  |
|  | described populations | described population in detail | | | |  | Yes |
|  | described interventions |  | described intervention in | | |  | Partial Yes |
|  | described comparators |  | detail (including doses where | | |  | No |
|  | described outcomes |  | relevant) | | |  |  |
|  |  | described comparator in detail | | | |  |  |
|  | described research designs |  |  |  |  |  |  |
|  |  |  | (including doses where | | |  |  |
|  |  |  |  |  |  |  |  |
|  |  |  | relevant) | | |  |  |
|  |  |  | described study’s setting | | |  |  |
|  |  | timeframe for follow-up | | | |  |  |

1. **Did the review authors use a satisfactory technique for assessing the risk of bias (RoB) in individual studies that were included in the review?**

| **RCTs** |  |  |  |  |  |
| --- | --- | --- | --- | --- | --- |
| For Partial Yes, must have assessed RoB | | For Yes, must also have assessed RoB | |  |  |
| From |  | from: |  |  |  |
|  | unconcealed allocation, *and* |  | allocation sequence that was |  | Yes |
| lack of blinding of patients and | |  | not truly random, *and* |  | Partial Yes |
|  | assessors when assessing |  | selection of the reported result |  | No |
|  | outcomes (unnecessary for |  | from among multiple |  | Includes only |
|  | objective outcomes such as all- |  | measurements or analyses of a |  | NRSI |
|  | cause mortality) |  | specified outcome |  |  |
| **NRSI** |  |  |  |  |  |
| For Partial Yes, must have assessed | | For Yes, must also have assessed RoB: | |  |  |
| RoB: |  |  | methods used to ascertain |  | Yes |
|  | from confounding, *and* |  | exposures and outcomes, *and* |  | Partial Yes |
|  | from selection bias |  | selection of the reported result |  | No |
|  |  |  | from among multiple |  | Includes only |
|  |  |  | measurements or analyses of a |  | RCTs |
|  |  |  | specified outcome |  |  |

1. **Did the review authors report on the sources of funding for the studies included in the review?**

**
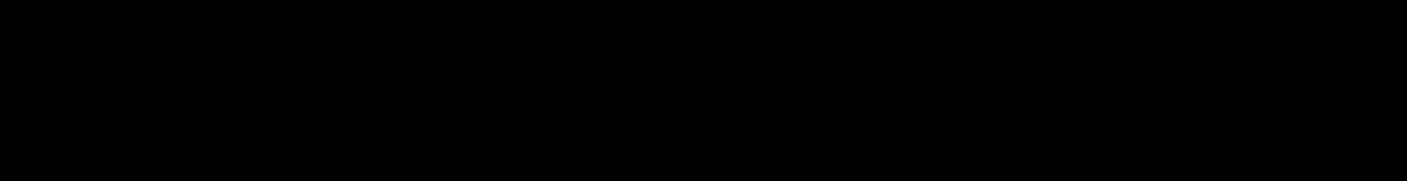

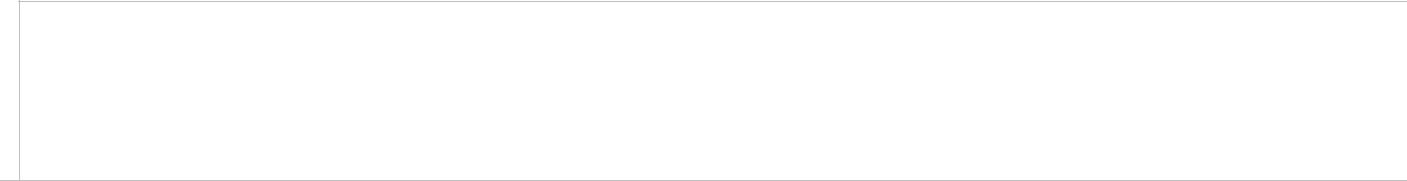
**

For Yes

Must have reported on the sources of funding for individual studies included in the review. Note: Reporting that the reviewers looked for this information but it was not reported by study authors also qualifies

Yes No

1. **If meta-analysis was performed did the review authors use appropriate methods for statistical combination of results?**

| **RCTs** |  |  |
| --- | --- | --- |
| For Yes: |  |  |
| The authors justified combining the data in a meta-analysis |  | Yes |
| AND they used an appropriate weighted technique to combine |  | No |
| study results and adjusted for heterogeneity if present. |  | No meta-analysis |
| AND investigated the causes of any heterogeneity |  | conducted |
| **For NRSI** |  |  |
| For Yes: |  |  |
| The authors justified combining the data in a meta-analysis |  | Yes |
| AND they used an appropriate weighted technique to combine |  | No |
| study results, adjusting for heterogeneity if present |  | No meta-analysis |
| AND they statistically combined effect estimates from NRSI that |  | conducted |
| were adjusted for confounding, rather than combining raw data, |  |  |
| or justified combining raw data when adjusted effect estimates |  |  |
| were not available |  |  |
| AND they reported separate summary estimates for RCTs and |  |  |
| NRSI separately when both were included in the review |  |  |

1. **If meta-analysis was performed, did the review authors assess the potential impact of RoB in individual studies on the results of the meta-analysis or other evidence synthesis?**

| For Yes: |  |  |
| --- | --- | --- |
| included only low risk of bias RCTs |  | Yes |
| OR, if the pooled estimate was based on RCTs and/or NRSI at variable |  | No |
| RoB, the authors performed analyses to investigate possible impact of |  | No meta-analysis |
| RoB on summary estimates of effect. |  | conducted |
|  | | |
| **13. Did the review authors account for RoB in individual studies when interpreting/ discussing the** | | |
| **results of the review?** |  |  |
|  |  |  |
| For Yes: |  |  |
| included only low risk of bias RCTs |  | Yes |
| OR, if RCTs with moderate or high RoB, or NRSI were included the |  | No |
| review provided a discussion of the likely impact of RoB on the results |  |  |
|  | | |
| **14. Did the review authors provide a satisfactory explanation for, and discussion of, any** | | |
| **heterogeneity observed in the results of the review?** |  |  |
|  |  |  |
| For Yes: |  |  |
| There was no significant heterogeneity in the results |  |  |
| OR if heterogeneity was present the authors performed an investigation of |  | Yes |
| sources of any heterogeneity in the results and discussed the impact of this |  | No |

on the results of the review

**15. If they performed quantitative synthesis did the review authors carry out an adequate investigation of publication bias (small study bias) and discuss its likely impact on the results of the review?**

For Yes:

performed graphical or statistical tests for publication bias and discussed Yes

the likelihood and magnitude of impact of publication bias No

No meta-analysis

conducted

1. **Did the review authors report any potential sources of conflict of interest, including any funding they received for conducting the review?**

| For Yes: | |  |  |
| --- | --- | --- | --- |
|  | The authors reported no competing interests OR |  | Yes |
|  | The authors described their funding sources and how they managed |  | No |
|  | potential conflicts of interest |  |  |

Note: Based on Shea et al. (2017).

***Table A 7.2: 3ie critical appraisal checklist***

| **Section A:** Methods used to identify, include and critically appraise studies | | | | | | | | | | | |  | | | | | |  | | | |
| --- | --- | --- | --- | --- | --- | --- | --- | --- | --- | --- | --- | --- | --- | --- | --- | --- | --- | --- | --- | --- | --- |
| **A1 Were the criteria used for deciding which studies to include in the review reported?** | | | **A2 Was the search for evidence reasonably comprehensive?** | | | | **A3 Does the review cover an appropriate time period?** | | | **A4 Was bias in the selection of articles avoided?** | | | | **A5 Did the authors use appropriate criteria to assess the quality and risk of bias in analysing the studies that are included?** | | | | | **A6 Overall – how much confidence do you have in the methods used to identify, include and critically appraise studies?** | | |
| Yes | | | Yes | | | | Yes | | | Yes | | | | Yes | | | | | Low Confidence | | |
| Partially | | | Partially | | | | Can't tell | | | Partially | | | | Partially | | | | | Medium Confidence | | |
| No | | | No | | | | No | | | No | | | | No | | | | | High Confidence | | |
|  | | | Can't tell | | | | Unsure | | |  | | | |  | | | | |  | | |
| *YES: All four should be yes NO: All four should be no PARTIALLY: Any other* | | | *YES: All five should be yes PARTIALLY: Relevant databases and reference lists are both reported NO: Any other* | | | | *YES: Generally this means searching the literature at least back to 1990 NO: Generally if the search does not go back to 1990 CAN’T TELL: No information about time period for search Note: With reference to the above – there may be important reasons for adopting different dates for the search, e.g. depending on the intervention. If you think there are limitations with the timeframe adopted for the search which have not been noted and justified by the authors, you should code this item as a NO and specify your reason for doing so in the comment box below. Older reviews should not be downgraded, but the fact that the search was conducted some time ago should be noted in the quality assessment. Always report the time period for the search in the comment box.* | | | *YES: All three should be yes, although reviews published in journals are unlikely to have a list of excluded studies (due to limits on word count) and the review should not be penalised for this. PARTIALLY: Independent screening and list of included studies provided are both reported NO: All other. If list of included studies provided, but the authors do not report whether or not the screening has been done by 2 reviewers review is downgraded to NO.* | | | | *YES: All three should be yes  PARTIALLY: The first and third criteria should be reported. If the authors report the criteria for assessing risk of bias and report a summary of this assessment for each criterion, but the criteria may be only partially sensible (e.g. do not address all possible risks of bias, but do address some), we downgrade to PARTIALLY.  NO: Any other* | | | | | *High confidence applicable when the answers to the questions in section A are all assessed as ‘yes’  Low confidence applicable when any of the following are assessed as ‘NO’ above: not reporting explicit selection criteria (A1), not conducting reasonably comprehensive search (A2), not avoiding bias in selection of articles (A4 , not assessing the risk of bias in included studies (A5)   Medium confidence applicable for any other – i.e. section A3 is assessed as ‘NO’ or can’t tell and remaining sections are assessed as ‘partially’ or ‘can’t tell’* | | |
| **Section B:** Methods used to analyse the findings | | | | | | |  | | |  | | | |  | | | | |  | | |
| **B1 Were the characteristics and results of the included studies reliably reported?** | | **B2 Are the methods used by the review authors to analyse the findings of the included studies clear, including methods for calculating effect sizes if applicable?** | | | | **B3 Did the review describe the extent of heterogeneity?** | | | **B4 Were the findings of the relevant studies combined (or not combined) appropriately relative to the primary question the review addresses and the available data?** | | **B5 Does the review report evidence appropriately?** | | | | **B6 Did the review examine the extent to which specific factors might explain differences in the results of the included studies?** | | | | | **B7 Overall - how much confidence do you have in the methods used to analyse the findings relative to the primary question addressed in the review?** | |
| Yes | | Yes | | | | Yes | | | Yes | | Yes | | | | Yes | | | | | Low Confidence | |
| Partially | | Partially | | | | Partially | | | Partially | | Partially | | | | Partially | | | | | Medium Confidence | |
| No | | No | | | | No | | | No | | No | | | | No | | | | | High Confidence | |
| Not applicable | | Not applicable | | | | Not applicable | | | Not applicable | | Not applicable | | | | Not applicable | | | | |  | |
|  | |  | | | |  | | | Can't tell | |  | | | |  | | | | |  | |
| *YES: All three should be yes PARTIALLY: Criteria one and three are yes, but some information is lacking on second criteria. No: None of these are reported. If the review does not report whether data was independently extracted by 2 reviewers (possibly a reporting error), we downgrade to NO. NOT APPLICABLE: if no studies/no data* | | *YES: Methods used clearly reported. If it is clear that the authors use narrative synthesis, they don't need to say this explicitly. PARTIALLY: Some reporting on methods but lack of clarity NO: Nothing reported on methods NOT APPLICABLE: if no studies/no data* | | | | *YES: First two should be yes, and third category should be yes if applicable should be yes PARTIALLY: The first category is yes NO: Any other NOT APPLICABLE: if no studies/no data* | | | *YES: If appropriate table, graph or meta-analysis AND appropriate weights AND unit of analysis errors addressed (if appropriate). PARTIALLY: If appropriate table, graph or meta-analysis AND appropriate weights AND unit of analysis errors not addressed (and should have been). NO: If narrative OR vote counting (where quantitative analyses would have been possible) OR inappropriate reporting of table, graph or meta-analyses. NOT APPLICABLE: if no studies/no data CAN’T TELL: if unsure (note reasons in comments below)* | | *YES: Both criteria should be fulfilled (where applicable) NO: Criteria not fulfilled PARTIALLY: Only one criteria fulfilled, or when there is limited reporting of quality appraisal (the latter applies only when inclusion criteria for study design are appropriate) NOT APPLICABLE: No included studies Note on reporting evidence and risk of bias: For reviews of effects of ‘large n’ interventions, experimental and quasi-experimental designs should be included (if available). For reviews of effects of ‘small n’ interventions, designs appropriate to attribute changes to the intervention should be included (e.g. pre-post with assessment of confounders)* | | | | *YES: Explanatory factors clearly described and appropriate methods used to explore heterogeneity PARTIALLY: Explanatory factors described but for meta-analyses, sub-group analysis or meta-regression not reported (when they should have been) NO: No description or analysis of likely explanatory factors NOT APPLICABLE: e.g. too few studies, no important differences in the results of the included studies, or the included studies were so dissimilar that it would not make sense to explore heterogeneity of the results* | | | | | *High confidence applicable when all the answers to the questions in section B are assessed as ‘yes’.   Low confidence applicable when any of the following are assessed as ‘NO’ above: critical characteristics of the included studies not reported (B1), not describing the extent of heterogeneity (B3), combining results inappropriately (B4), reporting evidence inappropriately (B5).  Medium confidence applicable for any other: i.e. the “Partial” option is used for any of the 6 preceding questions or questions and/or B.2 and/ or B.6 are assessed as ‘no’.* | |
| **Section C:** Overall assessment of the reliability of the review | | | | | | | | | | | | | |  | | | | | | | |
| **C1 Are there any other aspects of the review not mentioned before which lead you to question the results?** | | | | **C2 Are there any mitigating factors which should be taken into account in determining the reviews reliability?** | | | | | | **Comments** | | | | | | **C3 Based on the above assessments of the methods please provide a summary of the quality of the review/How would you rate the reliability of the review?** | | | | | |
| Additional methodological concerns – only one person reviewing | | | | Limitations acknowledged | | | | | |  | | | | | | Low | | | | | |
| Robustness | | | | | No strong policy conclusions drawn (including in abstract/ summary) | | | | | | | | | | | | Medium | | | | |
| Interpretation | | | | Any other factors | | | | | |  | | | | | | High | | | | | |
| Conflicts of interest (of the review authors or for included studies) | | | | |  | | | | | | | | |  | | | | | | | |
| Other | | | |  | | | | | |  | | | | | |  | | | | | |
| No other quality issues identified | | | |  | | | | | |  | | | | | | *High confidence in conclusions about effects: high confidence noted overall for sections A and B, unless moderated by answer to C1. Medium confidence in conclusions about effects: medium confidence noted overall for sections A or B, unless moderated by answer to C1 or C2. Low confidence in conclusions about effects: low confidence noted overall for sections A or B, unless moderated by answer to C1 or C2.  Limitations should be summarised above, based on what was noted in Sections A, B and C.* | | | | | |
| **Section D:** Methods used to analyse the causal chain and reach conclusions | | | | | | | | | | | | | |  | | | | |  | | |
| **D1 Does the review use a programme theory?** | **D2 Does the review incorporate qualitative evidence in the design?** | | | **D3 Did the review conduct analysis of intermediate and endpoint outcomes along causal chain?** | | | | **D4 Does the review incorporate qualitative evidence in the analysis?** | | **D5 Does the review incorporate qualitative evidence in other aspects of the analysis?** | | | **D6 Does the review integrate the findings from quantitative and qualitative evidence?** | | | **D7 Is quantitative and qualitative evidence integrated to form conclusions and implications?** | | | | | **D8 Overall - how much confidence do you have in the causal chain used in the review to analyse studies and the type of evidence incorporated to inform the analysis and reporting?** |
| Yes | Yes | | | Yes | | | | Yes | | Yes | | | Yes | | | Yes | | | | | Low Confidence |
| Partially | Partially | | | Partially | | | | Partially | | Partially | | | Partially | | | Partially | | | | | Medium Confidence |
| No | No | | | No | | | | No | | No | | | No | | | No | | | | | High Confidence |
| Not applicable | Not applicable | | | Not applicable | | | | Not applicable | | Not applicable | | | Not applicable | | | Not applicable | | | | |  |
|  |  | | | Can't tell | | | |  | |  | | |  | | |  | | | | |  |
| *YES: Some theory is used, whether an intervention level logic model or causal chain, or formal theory, and underlying assumptions are explicitly described. NO: None are reported. PARTIALLY: A theory is used but underlying assumptions are not reported.* | *YES: At least 1 and 2 or 3 are reported. NO: None are reported. PARTIALLY: 1 or 4 are reported.* | | | *YES: Boxes 1 and 2 are ticked PARTIALLY: Boxes 1 and 4 or 2 and 3 are ticked. NO: analysis of outcomes along causal chain is not undertaken and only endpoint outcomes are analysed (and outcomes at different stages of the causal chain were excluded). NOT APPLICABLE: if no studies/no data CAN’T TELL: if unsure (note reasons in comments below)* | | | | *YES: 1, 2, or 3 plus 4 or 5 are reported. NO: None are reported. PARTIALLY: Any other combination.* | | *YES: 1 or 2 are reported. NO: None are reported. PARTIALLY: 3 is reported.* | | | *YES: 1 and 2 or 3 are reported. NO: None are reported. PARTIALLY: 1 is reported only.* | | | *YES: All are reported. NO: None are reported. PARTIALLY: Only 1, 2 or 3 are reported.* | | | | | *High confidence applicable when the answers to the questions in section D are all assessed as ‘yes’  Low confidence applicable when any of the following are assessed as ‘NO’ above: not conducting analysis of intermediate and endpoint outcomes along the causal chain (D3), not incorporating qualitative evidence in the analysis (D5), not integrating the findings from quantitative and qualitative evidence (D6).*  *Medium confidence applicable for any other – i.e. section D3 is assessed as ‘NO’ or can’t tell and remaining sections are assessed as ‘partially’ or ‘can’t tell’* |

Notes: We adapted the 3ie tool and newly added section D to assess how well the studies addressed causal mechanisms in their analysis and subsequent discussions. Section D is based on an unpublished paper by Jimenez et al. (forthcoming).

### Appendix 8 – Quality assessment of included meta-studies, low confidence

| **No** | **Study** | **Synthesis approach** | **AMSTAR 2** | **3ie tool** | **Final decision** |
| --- | --- | --- | --- | --- | --- |
| 1 | Habib et al. 2016 | Systematic review | Critically low confidence | Low confidence | out (low) |
| 2 | Lorenzetti et al. 2017 | Systematic review | Low confidence | Low confidence | out (low) |
| 3 | Cole et al. 2012 | Systematic review | Low confidence | Low confidence | out (low) |
| 4 | Maîtrot & Niño-Zarazúa, 2017 | Systematic review | Critically low confidence | Low confidence | out (low) |
| 5 | Pande et al. 2012 | Systematic review | Critically low confidence | Low confidence | out (low) |
| 6 | Apostolakis et al. 2015 | Systematic review | Critically low confidence | Low confidence | out (low) |
| 7 | Arrivillaga & Salcedo, 2014 | Systematic review | Critically low confidence | Low confidence | out (low) |
| 8 | Bhageerathy et al. 2017 | Systematic review | Critically low confidence | Low confidence | out (low) |
| 9 | Awaworyi Churchill et al. 2016 | Meta-analysis | Critically low confidence | Low confidence | out (low) |
| 10 | Awaworyi Churchill, 2015 | Meta-analysis | Critically low confidence | Low confidence | out (low) |
| 11 | Madhani et al. 2015 | Systematic review | Critically low confidence | Low confidence | out (low) |
| 12 | Marr et al. 2016 | Systematic review | Critically low confidence | Low confidence | out (low) |
| 13 | O'Malley & Burke, 2017 | Systematic review | Critically low confidence | Low confidence | out (low) |
| 14 | Awaworyi Churchill, 2014 | Meta-analysis | Critically low confidence | Low confidence | out (low) |
| 15 | Gammage et al. 2017 | Systematic review | Critically low confidence | Low confidence | out (low) |
| 16 | Gash, 2017 | Systematic review | Critically low confidence | Low confidence | out (low) |
| 17 | Hidalgo, 2009 | Meta-analysis | Critically low confidence | Low confidence | out (low) |
| 18 | Isangula, 2012 | Systematic review | Critically low confidence | Low confidence | out (low) |
| 19 | O'Grady, 2016 | Systematic review | Critically low confidence | Low confidence | out (low) |
| 20 | Palmkvist & Lin, 2015 | Systematic review | Critically low confidence | Low confidence | out (low) |
| 21 | Yang & Stanley, 2013 | Meta-analysis | Critically low confidence | Low confidence | out (low) |

### Appendix 9 – Overview of quality assessment criteria for low-confidence meta-studies

| **3ie critical appraisal checklist** | **Yes** | **No** | **Partially/ can't tell** |
| --- | --- | --- | --- |
| Were the criteria used for deciding which studies to include in the review reported? (PICOS) | 11 | 1 | 9 |
| Was the search for evidence reasonably comprehensive? | 0 | 10 | 11 |
| Does the review cover an appropriate time period? | 12 | 3 | 6 |
| Was bias in the selection of articles avoided? | 3 | 17 | 1 |
| The criteria used for assessing the quality/ risk of bias were reported. | 0 | 0 | 21 |
| A table or summary of the assessment of each included study for each criterion was reported. | 7 | 14 | 0 |
| Did the authors use appropriate criteria to assess the quality and risk of bias in analysing the studies that are included? | 2 | 15 | 4 |
| Independent data extraction by at least 2 reviewers | 2 | 4 | 14 |
| Were the characteristics and results of the included studies reliably reported? | 1 | 17 | 3 |
| Are the methods used by the review authors to analyse the findings of the included studies clear, including methods for calculating effect sizes if applicable? | 0 | 0 | 21 |
| Did the review describe the extent of heterogeneity? | 2 | 18 | 1 |
| Were the findings of the relevant studies combined (or not combined) appropriately relative to the primary question the review addresses and the available data? | 1 | 4 | 16 |
| Does the review report evidence appropriately? | 2 | 19 | 0 |
| Limitations Acknowledged | 12 | 8 | 1 |
| Policy conclusions drawn (including in abstract/ summary) | 14 | 6 | 1 |
| Does the review use a programme theory? | 2 | 17 | 2 |
| Did the review conduct analysis of intermediate and endpoint outcomes along causal chain? | 1 | 17 | 3 |
| Does the review incorporate qualitative evidence in the analysis? | 1 | 10 | 10 |
| Does the review incorporate qualitative evidence in other aspects of the analysis? | 0 | 21 | 0 |
| Does the review integrate the findings from quantitative and qualitative evidence? | mostly N/A | mostly N/A | mostly N/A |
| Is quantitative and qualitative evidence integrated to form conclusions and implications? | mostly N/A | mostly N/A | mostly N/A |
|  |  |  |  |
|  |  |  |  |

| **A MeaSurement Tool to Assess systematic Reviews (AMSTAR 2)** | **Yes** | **No** | **Partial yes** |
| --- | --- | --- | --- |
| Did the research questions and inclusion criteria for the review include the components of PICO? | 2 | 19 | 0 |
| Did the report of the review contain an explicit statement that the review methods were established prior to the conduct of the review and did the report justify any significant deviations from the protocol? | 0 | 20 | 1 |
| Did the review authors explain their selection of the study designs for inclusion in the review? | 7 | 14 | 0 |
| Did the review authors use a comprehensive literature search strategy? | 2 | 11 | 8 |
| Did the review authors perform study selection in duplicate? | 3 | 18 | 0 |
| Did the review authors perform data extraction in duplicate? | 0 | 21 | 0 |
| Did the review authors provide a list of excluded studies and justify the exclusions? | 3 | 18 | 0 |
| Did the review authors describe the included studies in adequate detail? | 2 | 14 | 5 |
| Did the review authors use a satisfactory technique for assessing the risk of bias (RoB) in individual studies that were included in the review? RCTs | 1 | 20 | 0 |
| Did the review authors use a satisfactory technique for assessing the risk of bias (RoB) in individual studies that were included in the review? NRSI | 2 | 18 | 1 |
| Did the review authors report on the sources of funding for the studies included in the review? | 1 | 20 | 0 |
| If meta-analysis was performed did the review authors use appropriate methods for statistical combination of results? RCTs | 1 | 5 | 15x not MA |
| If meta-analysis was performed did the review authors use appropriate methods for statistical combination of results? NRSI | 0 | 6 | 15x not MA |
| If meta-analysis was performed, did the review authors assess the potential impact of RoB in individual studies on the results of the meta-analysis or other evidence synthesis? | 1 | 5 | 15x not MA |
| Did the review authors account for RoB in individual studies when interpreting/ discussing the results of the review? | 3 | 18 | 0 |
| Did the review authors provide a satisfactory explanation for, and discussion of, any heterogeneity observed in the results of the review? | 1 | 20 | 0 |
| If they performed quantitative synthesis did the review authors carry out an adequate investigation of publication bias (small study bias) and discuss its likely impact on the results of the review? | 5 | 3 | 13 |
| Did the review authors report any potential sources of conflict of interest, including any funding they received for conducting the review? | 8 | 13 | 0 |
